# Pleistocene–Holocene vicariance, not Anthropocene landscape change, explains the genetic structure of American black bear (*Ursus americanus*) populations in the American Southwest and northern Mexico

**DOI:** 10.1002/ece3.9406

**Published:** 2022-10-10

**Authors:** Matthew J. Gould, James W. Cain, Todd C. Atwood, Larisa E. Harding, Heather E. Johnson, Dave P. Onorato, Frederic S. Winslow, Gary W. Roemer

**Affiliations:** ^1^ Department of Fish, Wildlife and Conservation Ecology New Mexico State University Las Cruces New Mexico USA; ^2^ Department of Biology New Mexico State University Las Cruces New Mexico USA; ^3^ U.S. Geological Survey, Northern Rocky Mountain Science Center Bozeman Montana USA; ^4^ U.S. Geological Survey New Mexico Cooperative Fish and Wildlife Research Unit New Mexico State University Las Cruces New Mexico USA; ^5^ U.S. Geological Survey Alaska Science Center Anchorage Alaska USA; ^6^ Arizona Game and Fish Department Phoenix Arizona USA; ^7^ Fish and Wildlife Research Institute Florida Fish and Wildlife Conservation Commission Naples Florida USA; ^8^ New Mexico Department of Game and Fish Santa Fe New Mexico USA

**Keywords:** American black bear, American Southwest, landscape genetics, northern Mexico, Pleistocene, population genetic structure, *Ursus americanus*

## Abstract

The phylogeography of the American black bear (*Ursus americanus*) is characterized by isolation into glacial refugia, followed by population expansion and genetic admixture. Anthropogenic activities, including overharvest, habitat loss, and transportation infrastructure, have also influenced their landscape genetic structure. We describe the genetic structure of the American black bear in the American Southwest and northern Mexico and investigate how prehistoric and contemporary forces shaped genetic structure and influenced gene flow. Using a suite of microsatellites and a sample of 550 bears, we identified 14 subpopulations organized hierarchically following the distribution of ecoregions and mountain ranges containing black bear habitat. The pattern of subdivision we observed is more likely a product of postglacial habitat fragmentation during the Pleistocene and Holocene, rather than a consequence of contemporary anthropogenic barriers to movement during the Anthropocene. We used linear mixed‐effects models to quantify the relationship between landscape resistance and genetic distance among individuals, which indicated that both isolation by resistance and geographic distance govern gene flow. Gene flow was highest among subpopulations occupying large tracts of contiguous habitat, was reduced among subpopulations in the Madrean Sky Island Archipelago, where montane habitat exists within a lowland matrix of arid lands, and was essentially nonexistent between two isolated subpopulations. We found significant asymmetric gene flow supporting the hypothesis that bears expanded northward from a Pleistocene refugium located in the American Southwest and northern Mexico and that major highways were not yet affecting gene flow. The potential vulnerability of the species to climate change, transportation infrastructure, and the US–Mexico border wall highlights conservation challenges and opportunities for binational collaboration.

## Graphical abstract

The pattern of subdivision among black bears in the Southwest is more likely a product of postglacial habitat fragmentation during the Pleistocene and Holocene rather than a consequence of contemporary anthropogenic barriers to movement during the Anthropocene. We found significant asymmetric gene flow supporting the hypothesis that bears expanded northward from a Pleistocene refugium located in the American Southwest and northern Mexico and that major highways were not yet affecting gene flow. The potential vulnerability of the species to climate change, transportation infrastructure, and the U.S.‐Mexico border wall highlights conservation challenges and opportunities for binational collaboration.

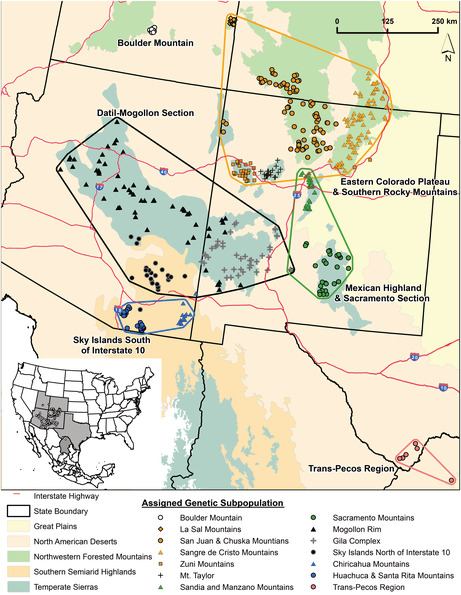

## 1 INTRODUCTION

The Pleistocene epoch (2.6–0.012 mya) represents a geologic period characterized by massive climatic fluctuations that drove dynamic glacial–interglacial cycles with profound effects on the global distribution and genetic structure of flora and fauna (Hofreiter & Stewart, 2009). Glacial advance contracted species' ranges into refugial pockets of habitat where isolation, selection, and genetic drift resulted in genetic differentiation among populations. Upon glacial recession, species expanded out of their respective refugia into their current distribution resulting in latitudinal patterns of species assemblages, genetic structure, and areas of admixture between formerly isolated populations (Lomolino et al., 1989; Puckett et al., 2015; Shafer et al., 2010). The phylogeographic influence of these glacial–interglacial dynamics has been observed for vagile species like the gray wolf (*Canis lupus*; Weckworth et al., 2010) and red fox (*Vulpes vulpes*; Aubry et al., 2009) and for more habitat‐restricted species like the woodland caribou (*Rangifer tarandus caribou*; Klütsch et al., 2012) and American marten (*Martes americana*; Stone et al., 2002 *)*. The location of refugia is highly dependent on the life history of the organism.

The American black bear (*Ursus americanus*; hereafter, black bear) is a large omnivore endemic to the forests of North America. Its distribution and genetic structure have been an ebb and flow of isolation and admixture events dictated by glacial tides of the Pleistocene (Puckett et al., 2015). Mitochondrial and nuclear data indicate that black bears were last isolated during the Last Glacial Maximum (LGM) ~26.5 kya and had contracted into three glacial refugia located in Beringia, the Pacific Northwest, and the American Southeast, and a fourth hypothesized refugium in the southwestern United States and northern Mexico (hereafter, the Southwest; Puckett et al., 2015; Varas‐Nelson, 2010). As glaciers receded (~20 kya), black bears expanded out of their respective refugia resulting in admixture among populations in west‐central and east‐central North America and the formation of region‐specific subpopulations (Pelletier et al., 2011; Puckett et al., 2015). Black bears across their northern range are genetically diverse and inhabit a large, contiguous landscape with a genetic structure that is consistent with isolation by distance due to female‐biased philopatry (Pelletier et al., 2011; Pelletier et al., 2017). Despite support for a fourth glacial refugium in the Southwest (Puckett et al., 2015; Varas‐Nelson, 2010), there remains some uncertainty as these inferences are based on limited geographic sampling, particularly in the southeast portion of New Mexico (Onorato et al., 2007; Onorato, Hellgren, Van Den Bussche, & Doan‐Crider, 2004). Furthermore, conflicting inferences regarding the genetic structure of black bears have been made in Arizona and New Mexico, where bear populations have been reported as both genetically structured and admixed (Atwood et al., 2011; Varas‐Nelson, 2010; Winslow, 2012).

The potential existence of a southwestern refugium for black bears is independently supported by paleoecological reconstruction of woodrat (*Neotoma* spp.) paleomiddens (Betancourt et al., 1990). Woodrats collect plant material and pollen blows in or adheres to plants and the macrofossils and pollen become encased in crystallized woodrat urine, or amberat, that form an indurated paleomidden (Spaulding et al., 1990). Investigations of these paleomiddens reveal information about the relative abundance, distribution, and species composition of prehistoric plant communities, which in turn enable assessments of climatic and plant community change (Betancourt et al., 1990). These investigations indicate that during the late Pleistocene and early Holocene (12 kya–present), areas within what are now the Chihuahuan and Sonoran deserts of the southwestern United States and northern Mexico, contained large areas dominated by pygmy conifer forest, a plant community comprising important food plants of black bears, including those that produce hard mast such as piñon pine (*Pinus* spp.), juniper (*Juniperus* spp.), and oak (*Quercus* spp.; Betancourt et al., 1990, Chp. 21; Holmgren et al., 2006; McAuliffe & Van Devender, 1998; Onorato et al., 2003). Conversely, areas farther north, in northern Arizona and New Mexico and southern Colorado and Utah, contained less hospitable habitat including montane glaciers, tundra, and taiga as well as forests dominated by yellow pine (*Pinus* spp.), limber pine (*P. flexilis*), and lodgepole pine (*P. contorta*). These regions currently harbor more contiguous black bear habitat. As the Holocene aridified, habitats preferred by bears either shifted up in elevation, such as in the Madrean Sky Islands along the US.–Mexico border, or farther north in latitude and the bears likely followed suit. Thus, vicariant events, namely climatic change that drove the distribution of important food plants for this forest‐adapted species, may have influenced the distribution of black bears. This pattern of vicariance and migration may be visible in the genetic structure of contemporary black bear populations.

Anthropogenic activities, in particular, overharvest, urbanization, and transportation infrastructure such as highways with high traffic volume have also influenced the abundance, movement patterns, and genetic structure of black bears. In the western and eastern portions of their range, overhunting and persecution during European settlement severely reduced the abundance of black bears with some populations recovering and recolonizing portions of their former range (Evans et al., 2017; Malaney et al., 2018), while others have been rendered into small, isolated populations more susceptible to genetic drift, eroding genetic diversity (Hooker et al., 2015; Murphy et al., 2017; Murphy et al., 2018). In Florida, major roads have heightened mortality (McCown et al., 2009) and acted as semipermeable barriers, that when coupled with urbanization, fragmented bear habitat, decreased connectivity, and caused appreciable genetic structure among subpopulations (Dixon et al., 2007; Karelus et al., 2017). In the Lower Mississippi Alluvial Valley of Louisiana, human‐caused mortality combined with extensive habitat loss and fragmentation forced black bears into a patchwork of small populations isolated by anthropogenic activities; active translocations are underway to help restore bear populations there (Murphy et al., 2018). In several states, roads have been shown to influence movements and patterns of habitat selection, as bears either avoid roads or select areas farther from roads, and in some regions, roads created genetic substructure by acting as filters to bear movement (Cushman & Lewis, 2010; Dixon et al., 2007; Gould et al., 2019; Hiller et al., 2015; Short Bull et al., 2011).

In the Southwest, limited geographic and genetic sampling has obscured the influence of prehistoric and contemporary ecological processes that shape genetic structure and govern gene flow of black bear populations. Our aim was to fill a crucial gap regarding the large‐scale population genetic structure of the American black bear by using a suite of microsatellite loci to characterize the genetic profile of 550 individual bears sampled across the Southwest. We focused on two hypotheses. First, we hypothesized that the current genetic structure could be a consequence of Pleistocene–Holocene vicariance whereby bears occupied forest refugia during the LGM, but then followed changes in the distribution of forests as the Holocene dried and warmed (Pleistocene–Holocene Vicariance Hypothesis). If true, we predicted that bears occupying contiguous forests should be relatively closely related and exhibit little genetic substructure. Bears in the Madrean Sky Islands should be more genetically structured (Atwood et al., 2011; Varas‐Nelson, 2010) and should show evidence of gene flow characterized by isolation by resistance. Finally, there should be a pronounced asymmetric pattern of gene flow from south to north. Second, we hypothesized that the genetic substructure of bears could be dominated by anthropogenic activities typifying the Anthropocene (i.e., the concept that we are living in a time when human activities have significant effecs on the global environment). If true, we predicted that the influence of major highways would be manifested by bears being more closely related on the same side of a highway and more distantly related on opposite sides. This should occur irrespective of the intervening habitat matrix. Although highways may not be barriers, they should act as semipermeable filters that influence gene flow (Anthropocene Filter Hypothesis). Expectations from these hypotheses may not be mutually exclusive, but we envision that the strength of evidence gathered through our analysis will expose their relative importance and that the insight gained will illuminate the processes that helped shape the phylogeography and present‐day genetic structure of black bears in the Southwest. Our findings will also aid in the conservation and management of black bears by identifying genetically isolated populations and the landscape features promoting or inhibiting genetic connectivity among black bear populations.

## 2 MATERIALS AND METHODS

### 2.1 Study area

**FIGURE 1:**
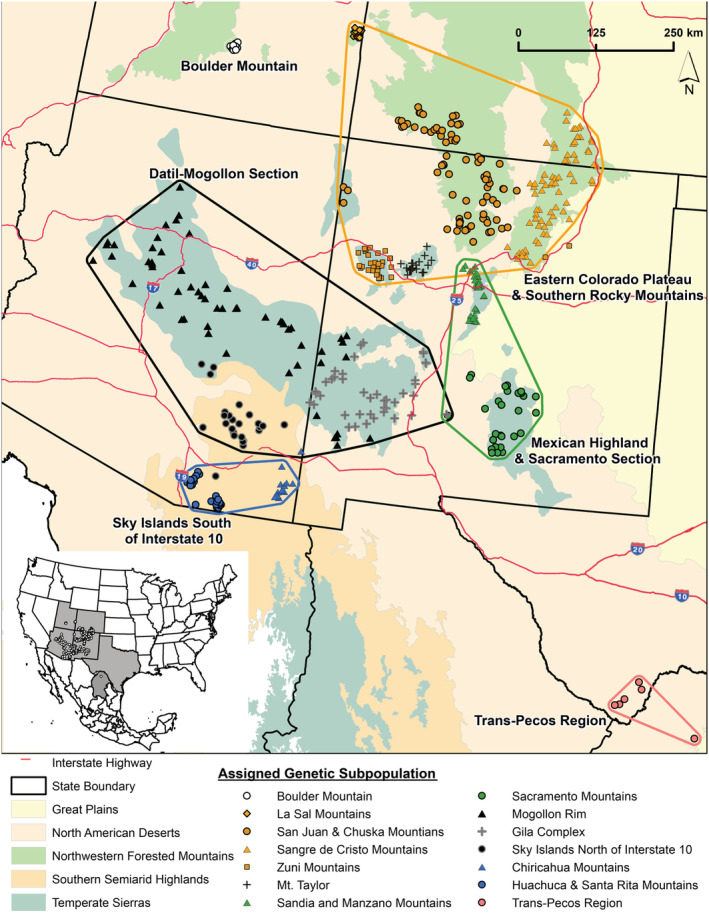
Distribution of genetic samples and subpopulations of American black bears (*Ursus americanus*) in the American Southwest and northern Mexico. geneland identified 6 and 14 subpopulations using the uncorrelated (polygons) and correlated (symbols) allele frequency models, respectively. The 6 larger subpopulations are named clusters.

We conducted our study in the southwestern United States (Arizona, Colorado, New Mexico, Texas, and Utah) and northern Coahuila de Zaragoza, Mexico (Figure 1). Orography and climate vary drastically throughout the Southwest with elevation ranging from 21 m at the southwest corner of Arizona to 4155 m‐high peaks in southern Colorado. The desert and grassland communities receive the majority of the ~100–300 mm of annual precipitation during the July to October monsoon season with mean monthly maximum temperatures for July from 1961 to 1990 ranging from ~16 to 40°C (Davey et al., 2006, 2007a, 2007b). The forest communities receive the majority of the ~300 to 1250 mm of annual precipitation during the winter with mean monthly minimum temperature for January from 1961 to 1990 ranging from ~ −20 to −4°C (Davey et al., 2006, 2007a, 2007b).

Black bears in the Southwest inhabit a mosaic of habitat distributed throughout three ecoregions: Northwestern Forested Mountains, Temperate Sierras, and Southern Semiarid Highlands which themselves are often separated by the North American Deserts ecoregion (Omernik & Griffith, 2014). Biotic communities at higher elevations and latitudes consist of Petran Subalpine and Petran Montane conifer forests transitioning to mid‐elevation Great Basin Conifer and Madrean Evergreen woodlands (Brown, 1994). Large expanses of low‐elevation valleys are composed of biotic communities such as Plains and Great Basin Grassland, Semidesert Grassland, and the Great Basin, Chihuahuan, and Sonoran desert scrub. These areas comprise a low elevation “sea” that is not typically used by black bears, isolating them on montane ‘sky islands’ (Brown, 1994; Hellgren et al., 2005; Olson et al., 2001).

### 2.2 Sample and marker selection

We collected genetic samples from individual black bears through hunter harvest, live‐capture, noninvasive genetic sampling, and vehicle collisions. We attempted to sample ≥ 25 individuals from each mountain range to obtain an adequate representation of allele frequency and diversity within each assumed subpopulation (Hale et al., 2012). Despite there being evidence that bears from west Texas and northern Coahuila de Zaragoza, Mexico, have genetic signatures more similar to bears in the southeast United States (Onorato et al., 2007; Onorato, Hellgren, Van Den Bussche, & Doan‐Crider, 2004; Pedersen et al., 2021, Van Den Bussche et al., 2009), we included individuals from this region in our analysis because limited geographic sampling of black bears in New Mexico creates the possibility of a link between west Texas and northern Coahuila de Zaragoza and New Mexico. At present, black bears from the Trans‐Pecos region have been documented in the Davis Mountains, Texas, ~150 km south of black bears found in the Guadalupe Mountains on the New Mexico and Texas border. We genotyped all individuals using the ZFX‐ZFY sex marker and 15 microsatellite loci (CXX20, G1A, G1D, G10B, G10C, G10H, G10J, G10L, G10M, G10O, G10P, G10U, G10X, MU50, and MU59; Durnin et al., 2007; Ostrander et al., 1993; Paetkau et al., 1998; Paetkau & Strobeck, 1995; Taberlet et al., 1997). Wildlife Genetics International in Nelson, British Columbia, Canada, generated all genotypes. Detailed laboratory protocols for microsatellite amplification and assignment error can be found in Paetkau (2003) and Gould et al. (2018). We obtained permits under the Convention on International Trade in Endangered Species (Export Permits 12US86418A/9, 13US19950B/9, 13US199551B/9, 14US43944B/9, 15US61420B/9, 15US69493B/9, 15US69502B/9) to export samples to Canada for analysis. Our research was authorized by the New Mexico Department of Game and Fish (Taking Protected Wildlife for Scientific and or Education Purposes Permit 3504) and approved by the New Mexico State University Institutional Animal Care and Use Committee (Protocol number 2011‐027).

### 2.3 Describing genetic structure and estimating gene flow

We conducted all genetic analyses in program r version 3.5.2 unless otherwise specified (R Core Team, 2018). We tested for linkage disequilibrium (LD) using the r package genepop and null alleles and deviations from Hardy–Weinberg equilibrium (HWE) using the r package popgenreport (version 3.0.0; Adamack & Gruber, 2014; version 1.0.5; Rousset et al., 2017). We conducted a significance test for null alleles by assessing if a bootstrapped 95% confidence intervals (CI) for each locus overlapped zero, whereby overlap would indicate that the frequency of null alleles does not differ significantly from zero. We applied a Bonferroni correction (*α* = 0.05 divided by the number of pairwise comparisons for each test) of *α* ≤ 0.0005 (LD) and *α* ≤ 0.003 (HWE) to reduce the likelihood of a false‐positive significance test, and we generated allele‐frequency statistics for each locus in popgenreport. For each identified subpopulation, we quantified genetic diversity using unadjusted private alleles (*A*_P_) and private alleles using rarefaction (*A*_PR_), which accounts for differences in sample size among subpopulations, using hp‐rare v1.0 (Kalinowski, 2004, 2005). We also quantified genetic diversity by estimating expected (*H*_E_) and observed (*H*_O_) heterozygosity and allelic richness using rarefaction (*A*_R_) with the r package diversity (version 1.9.9; Keenan et al., 2013). We used diversity to calculate deviations from random mating (*F*_IS_) and genetic differentiation among subpopulations (*F*_ST_) along with their 95% CI based on 1000 bootstrap iterations. We classified values of *F*_ST_ from 0.05–0.14, 0.15–0.24, and ≥0.25 as moderate, high, and very‐high differentiation, respectively, and considered differentiation to be biologically meaningful if the lower CI was ≥0.05 (Hartl & Clark, 1997).

We used two Bayesian clustering programs to characterize population structure, geneland and structure (Guillot et al., 2005; Pritchard, 2000). Both programs use multi‐locus genotypes to infer the number of genetic subpopulations (K) maintaining both Hardy–Weinberg and linkage equilibrium. geneland, however, uses spatial data to infer the spatial boundaries that separate the K subpopulations (Guillot et al., 2005). Because geneland has been shown to outperform other Bayesian clustering methods in detecting barriers to dispersal in fewer generations for species with higher dispersal abilities (Blair et al., 2012; Safner et al., 2011), we based our inferences on geneland. Results from the structure analysis were similar and are available, along with the methods, in Appendix S1. We performed 10 independent runs using the uncorrelated and correlated allele frequency models. We used both models because the former is less sensitive to departures from model assumptions while the latter is more apt to detect subtle genetic differentiation (Guillot et al., 2008; The Geneland Development Group, 2018). We varied K from 1 to 31 (the maximum number of sampling locations +1) and then used the model with the highest mean posterior probability to select K and assigned individuals to the population in which their estimated proportion of ancestry (i.e., the *Q*‐value) was the greatest. We optimized all models using 500,000 Markov Chain Monte Carlo iterations, 1000 burn in, a 100‐iteration thinning interval, an uncertainty of 2 km for GPS coordinates, and a maximum rate of 1650 nuclei for the Poisson‐Voronoi tessellation (three times the number of individuals). We implemented our analysis in the r package geneland using program r (version 3.4.4; R Core Team, 2018; The Geneland Development Group, 2018). After assessing population structure, we again assessed for LD, null alleles, and deviations from HWE, and if these tests failed then we reassessed for population structure until tests were not significant.

#### 2.3.1 Environmental variables

Available food resources should influence habitat selection, as black bears must accumulate large‐fat stores for both hibernation and reproduction (Costello et al., 2003) and food for bears in arid environments is tied to precipitation (precip; Zlotin & Parmenter, 2008). Black bears are forest obligates and have evolved morphological and behavioral adaptations associated with exploiting forest stands (Herrero, 1972) and they require thermal refugia (Lara‐Díaz et al., 2018) because they are susceptible to hyperthermia (Sawaya et al., 2017). We modeled these features using canopy height (canopy), percent canopy (percan), and water bodies (water) as canopy provides thermal cover and water is necessary for thermoregulation, especially if bears crossed more inhospitable land cover such as desert. Male black bears have been shown to use less rugged areas (Costello, 2010; Johnson et al., 2015; Onorato, Hellgren, Van Den Bussche, & Skiles Jr., 2004), so we used a Terrain Ruggedness Index (TRI) to represent potential movement corridors. Linear‐water features (streams) contain food, escape, and thermal cover, and are travel corridors (Atwood et al., 2011; Johnson et al., 2015). Roads can elicit negative behavioral and genetic effects and can influence bear distribution (Dixon et al., 2007; Gould et al., 2019), so we assessed their effect by estimating road density (rd. density). Interstates and highways (rd. major), which if not acting as barriers, may inhibit gene flow by heightening mortality rates (Little et al., 2017). We did not explore the influence of other anthropogenic activities such as agriculture or human settlements because the former is uncommon and sparsely distributed across New Mexico while the latter is correlated with road density.

We determined the spatial extent of the environmental variables, and subsequent resistance surfaces, by buffering all sample locations by 61 km based on the maximum‐dispersal distance for black bears in the Sangre de Cristo and Mogollon mountains, New Mexico (Costello, 2010). We calculated mean‐summer precipitation (Apr–Sep; continuous covariate), using WorldClim2 monthly precipitation levels from 1970 to 2000 (http://worldclim.org/version2; Fick & Hijmans, 2017). We obtained percent canopy (continuous covariate) at a 30 m resolution using the U.S Geological Survey Global Tree Canopy Cover dataset (https://www.landcover.usgs.gov; Hansen et al., 2013). We obtained canopy height (continuous covariate) at a 1 km resolution using the National Aeronautics and Space Administration EARTHDATA Spatial Data Access Tool (https://daac.ornl.gov). We obtained location data for streams (binary covariate) and water bodies (binary covariate) from the National Hydrography Dataset (https://www.usgs.gov/core‐science‐systems/ngp/national‐hydrography). We derived TRI (continuous covariate) using a National Elevation Dataset 30 m digital elevation model (www.nationalmap.gov) and the Benthic Terrain Modeler in arcmap. We calculated road density (km/25 km^2^; continuous covariate) and major roads (categorical covariate) using data from Open Street Map (www.openstreetmap.org). For major roads, we used three classifications: interstate highways, state highways, and county roads. We resampled each resistance layer to a 5 km resolution using bilinear interpolation to reduce the computational intensity of the optimization process given the large extent of the study area without sacrificing an accurate characterization of the landscape (McRae et al., 2008). We created and manipulated all resistance surfaces using arcmap v10.4.1 (Environmental Systems Research Institute, Redlands, CA, USA). We assessed correlation among covariates using a Pearson's correlation coefficient of *r* ≥ |0.60|. We found a correlation between canopy height and percent canopy (*r* = 0.72) and removed the latter from our analysis.

#### 2.3.2 Generating the resistance surface

We used the r package resistancega to optimize resistance surfaces, assess the effect of pairwise‐effective distance on pairwise‐genetic distance, and conduct model selection while accounting for non‐independence among the pairwise data (version 4.1‐0.2.1; Peterman, 2018; Peterman et al., 2014). resistancega optimizes resistance surfaces using a genetic algorithm (a process based on the theory of natural selection) that eliminates the subjective assignment of resistance values by expert opinion and the limited exploration of the optimized parameter space (Peterman, 2018; Peterman et al., 2014). The optimization process begins with the selection of parameter values that control the transformation, shape of the transformation, and resistance value for a continuous surface, or if a categorical surface, the assignment of values to each resistance level. After each iteration, pairwise effective distances among all individuals are calculated and a linear mixed‐effects model is then fit to the data where effective distance is used to predict genetic distance among individuals (Clarke et al., 2002; Peterman, 2018; Peterman et al., 2014). The relative support for the combination of parameter values at each iteration is assessed using an objective function from the mixed‐effects model, and once the objective function can no longer be improved, surface optimization is completed.

We quantified effective landscape distance using random‐walk commute times in the r package gdistance (version 1.2–2; Van Etten, 2017). We quantified pairwise‐genetic distance using the individual‐based metric proportion of shared alleles (*D*_ps_) in the r package adegenet (version 2.1.1; Jombart, 2008). We applied a monomolecular and Ricker transformation along with their inverse, reverse, and inverse‐reverse forms to each continuous‐resistance covariate to explore the functional relationship between each covariate and resistance to movement. We constructed our model using a maximum of three covariates due to computational intensity and assessed the relative support among resistance surface models using Akaike's Information Criterion adjusted for small sample size (AICc) with models >2 AICc units from the top model being discounted (Burnham & Anderson, 2002; Hurvich & Tsai, 1989) and by calculating the model weight (*w*_*i*_). We conducted the optimization process in program r (version 3.4.4; R Core Team, 2017) using the Bridges high‐performance computing system at the Pittsburgh Supercomputing Center (Nystrom et al., 2015; Towns et al., 2014).

To explore how geographic distance vs. landscape distance affects pairwise‐genetic distance, we used AIC_C_ to rank the fit of linear mixed‐effects models using Euclidean distance, the top‐ranked resistance surface, and a model that combined both.

#### 2.3.3 Relative degree and direction of gene flow

We investigated asymmetric gene flow by estimating relative migration among the estimated subpopulations using the divMigrate function in the r package diversity where maximum relative gene flow is set at 1 and minimum at 0 (Keenan et al., 2013; Sundqvist et al., 2016). We calculated *G*_ST_, a measure of population differentiation and an analog of *F*_ST_, for network plots, conducted 1000 bootstrap iterations to generate 95% CIs to evaluate if asymmetric gene flow was significant and chose to display connections ≥0.50.

## 3 RESULTS

### 3.1 Describing genetic structure

We genotyped 550 (285M:265F) individuals from 28 localities (Appendix S2: Table S1). We found a moderate percentage of null alleles (4–12%) across all loci in this total sample (Appendix S2: Table S2). We found 82% of the pairwise comparisons among loci (*n* = 105) for LD to be significant (*P* < 0.0005 after Bonferroni correction) and all loci were out of HWE (*p* < .003 after Bonferroni correction; Appendix S2: Tables S3–S4). These metrics suggest that genetic structuring may occur among black bears across the Southwest.

the uncorrelated allele frequency model in geneland identified 6‐regional genetic clusters: Boulder Mountain, Utah (BM), the eastern Colorado Plateau and Southern Rocky Mountains (ECPSRM), the Datil‐Mogollon Section (DMS), the Mexican Highland and Sacramento sections (MHSS), the Sky Islands south of Interstate 10 (SIS), and the Trans‐Pecos region (TP; Figure 1; Appendix S2: Figure S1). The presence of null alleles was suggested at loci CXX20 (BM), G10H (ECPSRM), G10J (SIS and TP), G10O (MHSS and SIS), and MU50 (BM and DMS; Appendix S3: Table S1). We found the CXX20 locus to be non‐randomly associated with G10B and G10H in the BM subpopulation (Appendix S3: Tables S2–S4). The G10U and G10L loci were out of HW proportions in the ECPSRM and DMS, respectively (Appendix S3: Table S5). The presence of null alleles and linkage disequilibrium suggests these loci may not accurately represent genetic structure and diversity while deviations from HW proportions suggest there could be additional genetic structure that was not detected under the uncorrelated allele frequency model.

Allelic richness was lowest in the SIS (*A*_R_ = 3.91) and highest in the DMS (*A*_R_ = 5.43); the TP had the second highest allelic richness despite small sample size (Table 1). The number of private alleles using rarefaction was lowest in DMS (*A*_PR_ = 0.09) and highest in the TP (*A*_PR_ = 1.79; Table 1; Appendix S3: Table S6). Observed heterozygosity ranged from 0.42 to 0.64 was slightly lower than *H*_E_ (0.44–0.62) for all regional subpopulations except for the TP (Table 1). The *F*_IS_ estimates suggested deviations from random mating within the ECPSRM and DMS subpopulations, but along with *H*_O_ being lower than *H*_E_ for both subpopulations, it is more likely that a Wahlund effect, rather than nonrandom mating, is occurring, which indicates greater substructure within these two regions (Wahlund, 1928). Genetic differentiation was the lowest between the ECPSRM and DMS (*F*_ST_ = 0.03) and highest between the TP and SIS (*F*_ST_ = 0.44). Overall, the two most isolated subpopulations, BM and the TP, displayed the highest levels of genetic differentiation compared to all other subpopulations (Table 2).

**TABLE 1:** Number of individuals (*N*), private alleles (*A*_P_), private alleles using rarefaction (*A*_PR_), allelic richness using rarefaction (*A*_R_), observed (*H*_O_) and expected (*H*_E_) heterozygosity, and a measure of deviations from random mating (*F*_IS_) and its 95% confidence interval (LCI and UCI) based on 1000 bootstrap iterations for American black bear (*Ursus americanus*) subpopulations in the American Southwest and northern Mexico.

Subpopulation	Acronym	State	*N*	*A*_P_	*A*_PR_	*A*_R_	*H*_O_	*H*_E_	*F*_IS_	LCI	UCI
Regional											
Boulder Mountain	BM	UT	21	3	0.41	4.25	0.56	0.58	0.02	−0.08	0.08
Eastern Colorado Plateau and Southern Rocky Mountains	ECPSRM	CO/NM/UT	142	8	0.26	4.61	0.48	0.50	0.04	0.01	0.06
Datil‐Mogollon Section	DMS	AZ/NM	247	2	0.09	5.43	0.57	0.59	0.04	0.01	0.05
Mexican Highland and Sacramento sections	MHSS	NM	65	2	0.19	4.66	0.56	0.58	0.02	−0.03	0.06
Sky Islands South of Interstate 10	SIS	AZ	55	0	0.21	3.91	0.42	0.44	0.04	−0.02	0.08
Trans‐Pecos region	TP	TX	20	21	1.79	5.07	0.64	0.62	−0.03	−0.14	0.02
Mountain Range											
Boulder Mountain	BM	UT	21	3	0.25	4.14	0.56	0.58	0.02	−0.09	0.07
La Sal Mountains	LSM	UT	28	1	0.07	4.63	0.56	0.58	0.01	−0.07	0.05
San Juan and Chuska mountains	SJC	CO/NM	82	1	0.06	4.89	0.56	0.57	0.01	−0.03	0.04
Sangre de Cristo Mountains	SCM	CO/NM	81	1	0.09	4.73	0.61	0.59	−0.02	−0.06	0.00
Zuni Mountains	ZM	NM	33	0	0.04	5.01	0.54	0.53	0.00	−0.07	0.03
Mt. Taylor	MT	NM	23	1	0.03	4.36	0.55	0.54	0.00	−0.11	0.06
Sandia and Manzano mountains	SMM	NM	34	1	0.02	4.34	0.57	0.57	0.00	−0.07	0.03
Mogollon Rim	MR	AZ	63	0	0.01	4.26	0.50	0.52	0.03	−0.02	0.07
Gila complex	GC	NM	44	1	0.04	3.95	0.46	0.47	0.02	−0.05	0.05
Sacramento Mountains	SM	NM	31	1	0.04	4.05	0.55	0.55	−0.02	−0.09	0.02
Sky Islands North of Interstate 10	SIN	AZ	35	0	0.02	4.25	0.48	0.48	−0.01	−0.07	0.03
Huachuca and Santa Rita mountains	HSRM	AZ	39	0	0.02	3.45	0.39	0.40	0.03	−0.04	0.08
Chiricahua complex	CHC	AZ	16	0	0.04	3.87	0.48	0.45	−0.06	−0.18	0.01
Trans‐Pecos region	TP	TX	20	21	1.39	4.89	0.64	0.62	−0.03	−0.13	0.02

**TABLE 2:** Estimated pairwise genetic differentiation (*F*_ST_) and their 95% confidence intervals based on 1000 bootstrap iterations for regional subpopulations identified by geneland using the uncorrelated allele frequency model for American black bears (*Ursus americanus*) in the American Southwest and northern Mexico.

	BM	ECPSRM	DMS	MHSS	SIS	TP
BM	**–**					
ECPSRM	**0.14 (0.10–0.17)**	**–**				
DMS	**0.21 (0.17–0.25)**	0.03 (0.03–0.04)	**–**			
MHSS	**0.16 (0.12–0.19)**	0.04 (0.03–0.05)	**0.06 (0.05–0.08)**	**–**		
SIS	**0.25 (0.21–0.29)**	**0.09 (0.08–0.11)**	**0.09 (0.07–0.11)**	**0.11 (0.10–0.13)**	**–**	
TP	**0.30 (0.27–0.34)**	**0.33 (0.31–0.35)**	**0.40 (0.38–0.43)**	**0.33 (0.31–0.36)**	**0.44 (0.42–0.47)**	**–**

*Notes*: Bolded values signify statistically significant differentiation.

Abbreviations: BM, Boulder Mountain; DMS, Datil‐Mogollon Section; ECPSRM, Eastern Colorado Plateau and Southern Rocky Mountains; MHSS, Mexican Highland and Sacramento sections; SIS, Sky Islands South of Interstate 10; TP, Trans‐Pecos.

the correlated allele frequency model identified 14‐genetic clusters that closely tracked the sampled mountain ranges (Appendix S2: Figure S1). The BM and TP subpopulations from the regional results remained while the larger clusters were broken down into 12 subpopulations (Figure 1). We did not find evidence of null alleles (Appendix S4: Table S1–S8). All loci within each respective subpopulation were in HWE (Appendix S4: Table S9). Because there was no discernable pattern of null alleles, LD, or HW disequilibrium for ≥1 locus at ≥1 subpopulation we retained all loci in our analyses (Morin et al., 2010).

The TP subpopulation retained the highest number of private alleles (*A*_PR_ = 1.39) while the Mogollon Rim (MR) was estimated to have the least number of private alleles (*A*_PR_ = 0.01; Table 1). The Chiricahua complex (CHC) and the Huachuca‐Santa Rita mountains (HSRM) subpopulations both exhibited a fixed allele (122 bp) at the MU50 locus (Appendix S4: Table S10), these two subpopulations are south of Interstate 10. Heterozygosity was lowest in the HRSM and highest in the TP (Table 1). The *F*_IS_ estimates did not suggest deviations from random mating (Table 1). Pairwise differentiation was high or very‐high when subpopulations were compared to the BM, HSRM, and TP subpopulations (*F*_ST_ ≥ 0.15; Table 3), this was not unexpected as both the BM and TP populations are isolated from the other populations. The La Sal Mountains (LSM), another somewhat isolated subpopulation along the Utah–Colorado border, was moderately differentiated from all other subpopulations except for three subpopulations to the south: the Sangre de Cristo Mountains (SCM), the San Juan and Chuska mountains (SJC), and Zuni Mountains (ZM; Table 3), all relatively close geographically. Genetic differentiation was low to moderate among the remaining subpopulations (Table 3).

**TABLE 3:** Estimated pairwise genetic differentiation (*F*_ST_) and their 95% confidence intervals based on 1000 bootstrap iterations for mountain range subpopulations identified by geneland using the correlated allele frequency model for American black bears (*Ursus americanus*) in the American Southwest and northern Mexico.

	BM	LSM	SJC	SCM	ZM	MT	SMM
BM	**–**						
LSM	**0.15 (0.11–0.19)**	**–**					
SJC	**0.15 (0.11–0.19)**	0.04 (0.02–0.06)	**–**				
SCM	**0.14 (0.11–0.18)**	0.05 (0.03–0.07)	0.03 (0.02–0.04)	**–**			
ZM	**0.18 (0.13–0.22)**	0.06 (0.04–0.09)	0.03 (0.01–0.04)	0.04 (0.03–0.06)	**–**		
MT	**0.18 (0.14–0.24)**	**0.08 (0.05–0.11)**	0.03 (0.01–0.05)	**0.06 (0.05–0.09)**	0.04 (0.02–0.08)	**–**	
SMM	**0.17 (0.13–0.21)**	**0.08 (0.06–0.10)**	0.04 (0.03–0.06)	0.04 (0.03–0.06)	0.04 (0.02–0.07)	0.04 (0.02–0.06)	**–**
MR	**0.20 (0.16–0.24)**	**0.09 (0.07–0.11)**	0.05 (0.04–0.06)	**0.06 (0.05–0.08)**	0.02 (0.01–0.03)	0.05 (0.03–0.09)	**0.06 (0.05–0.08)**
GC	**0.23 (0.19–0.27)**	**0.09 (0.07–0.12)**	0.05 (0.04–0.06)	**0.07 (0.06–0.09)**	0.02 (0.01–0.04)	0.06 (0.04–0.09)	0.06 (0.04–0.09)
SM	**0.16 (0.13–0.21)**	**0.10 (0.08–0.12)**	**0.07 (0.06–0.09)**	**0.08 (0.07–0.10)**	**0.09 (0.06–0.12)**	**0.07 (0.05–0.09)**	0.04 (0.02–0.07)
SIN	**0.20 (0.16–0.24)**	**0.09 (0.07–0.11)**	0.06 (0.04–0.07)	**0.07 (0.05–0.08)**	0.03 (0.01–0.04)	0.07 (0.04–0.10)	**0.07 (0.05–0.09)**
HSRM	**0.27 (0.23–0.32)**	**0.15 (0.13–0.18)**	**0.14 (0.13–0.16)**	**0.13 (0.12–0.15)**	**0.12 (0.10–0.15)**	**0.16 (0.13–0.19)**	**0.13 (0.11–0.16)**
CHC	**0.22 (0.18–0.27)**	**0.11 (0.09–0.14)**	**0.10 (0.07–0.12)**	**0.09 (0.07–0.12)**	**0.07 (0.05–0.11)**	**0.13 (0.09–0.18)**	**0.11 (0.08–0.14)**
TP	**0.30 (0.27–0.34)**	**0.33 (0.30–0.37)**	**0.34 (0.32–0.37)**	**0.32 (0.30–0.35)**	**0.37 (0.33–0.40)**	**0.36 (0.33–0.40)**	**0.34 (0.32–0.37)**

	MR	GC	SM	SIN	HSRM	CHC	TP
BM							
LSM							
SJC							
SCM							
ZM							
MT							
SMM							
MR	**–**						
GC	0.01 (0.00–0.03)	**–**					
SM	**0.09 (0.07–0.11)**	**0.11 (0.08–0.13)**	**–**				
SIN	0.02 (0.00–0.04)	0.03 (0.02–0.06)	**0.09 (0.07–0.12)**	**–**			
HSRM	**0.12 (0.10–0.15)**	**0.15 (0.12–0.18)**	**0.17 (0.14–0.20)**	**0.10 (0.08–0.13**)	**–**		
CHC	**0.08 (0.05–0.12)**	**0.09 (0.05–0.13)**	**0.13 (0.10–0.16)**	0.06 (0.03–0.10)	**0.08 (0.05–0.12)**	**–**	
TP	**0.38 (0.36–0.41)**	**0.41 (0.38–0.45)**	**0.34 (0.31–0.37)**	**0.40 (0.38–0.44)**	**0.46 (0.43–0.49)**	**0.40 (0.37–0.43)**	**–**

*Notes*: Bolded values signify statistically significant differentiation.

Abbreviations: BM, Boulder Mountain; CHC, Chiricahua complex; GC, Gila complex; HSRM, Huachuca and Santa Rita mountains; LSM, La Sal Mountains; MR, Mogollon Rim; MT, Mt. Taylor; SM, Sacramento Mountains; SJC, San Juan and Chuska mountains; SMM, Sandia and Manzano mountains; SCM, Sangre de Cristo Mountains; SIN, Sky Islands north of Interstate 10; TP, Trans‐Pecos region; ZM, Zuni Mountains.

### 3.2 Landscape features regulating gene flow

The top‐ranked resistance‐surface model was well supported (*w*_*i*_ = 1.00), substantially outperformed the second‐ranked model (ΔAIC_c_ = 47.18), and included canopy, precipitation, and TRI (Appendix S5: Tables S1 and S2). The transformations that best represented the relationship between canopy, precipitation, and TRI with resistance to movement were the inverse monomolecular, inverse Ricker, and monomolecular, respectively, indicating that resistance decreased as canopy increased, decreased as precipitation increased until the covariate reached moderate levels at which point resistance started to increase, and increased as TRI increased (Table 4). More simply, resistance was lowest in areas with higher forest canopy, higher levels of precipitation, and less rugged landscapes. Precipitation contributed the most to the top‐ranked resistance surface (58%) followed by canopy (40%) with a small contribution from TRI (2%). This top‐ranked model received considerable support when compared to Euclidean distance alone, suggesting isolation by resistance better explained the observed‐genetic pattern than isolation by distance (Table 4). A model composed of both effective and Euclidean distance, however, outperformed (*w*_*i*_ = 1.00) the top‐ranked resistance model suggesting isolation by distance is still an important component explaining genetic distance (Table 4). Our analysis did not show support for any resistance‐based models that included road density or major roads (Appendix S5: Tables S1 and S2).

**TABLE 4:** Model selection results for two optimization runs derived using Akaike's Information Criterion corrected for small sample size (AIC_c_) comparing the top‐ranked resistance surface optimized using linear mixed‐effects models with maximum likelihood population effects parameterization to models composed of Euclidean distance (Distance Only) and Euclidean plus the top‐ranked resistance surface (Top resistance surface + Distance).

Model	AIC_c_	ΔAIC_c_	*w*_*i*_	Contribution	Transformation	Shape	Magnitude
Optimization run 1							
Top resistance surface + Distance	−388853.00	0.00	1.00	–	–	–	–
Top resistance surface	−387083.80	1769.20	0.00	–	–	–	–
Canopy height	–	–		40	Inverse Monomolecular	0.51	1272.21
Precipitation	–	–		58	Inverse Ricker	3.33	1585.36
Terrain ruggedness index	–	–		02	Monomolecular	2.87	318.72
Distance Only	−378750.20	10102.80	0.00	–	–	–	–
Optimization run 2							
Top resistance surface + Distance	−388853.00	0.00	1.00	–	–	–	–
Top resistance surface	−387096.20	1756.80	0.00	–	–	–	–
Canopy height	–	–		40	Inverse Monomolecular	0.51	1272.21
Precipitation	–	–		58	Inverse Ricker	3.33	1585.36
Terrain ruggedness index	–	–		02	Monomolecular	2.87	318.72
Distance Only	−378750.20	10102.80	0.00	–	–	–	–

*Notes*: We ranked models by the difference in AIC_c_ (ΔAIC_c_) between the top model and competing models and evaluated model support using model weights (*w*_*i*_). Optimization results are also reported including the percent contribution of each covariate to the total surface resistance (Contribution), transformation applied to each covariate (Transformation) along with the shape and magnitude of each transformed covariate.

### 3.3 Relative degree and direction of genetic connectivity

**FIGURE 2:**
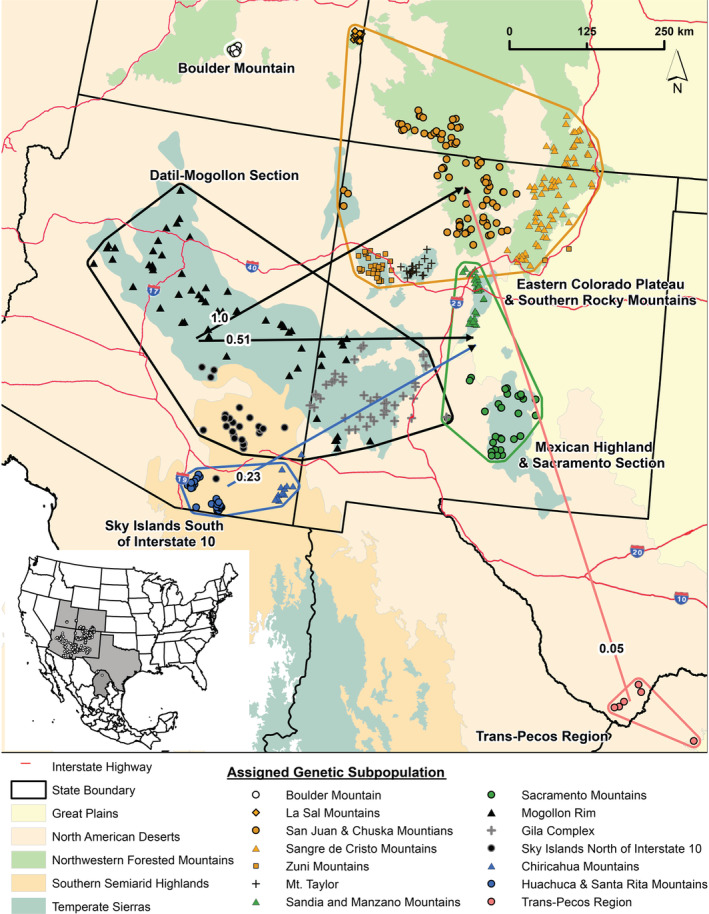
Directional relative migration network based on *G*_ST_ values for American black bear (*Ursus americanus*) subpopulations in the American Southwest and northern Mexico. The network visualized shows significant asymmetrical migration values for subpopulations identified using the uncorrelated allele frequency model in program geneland.

**FIGURE 3:**
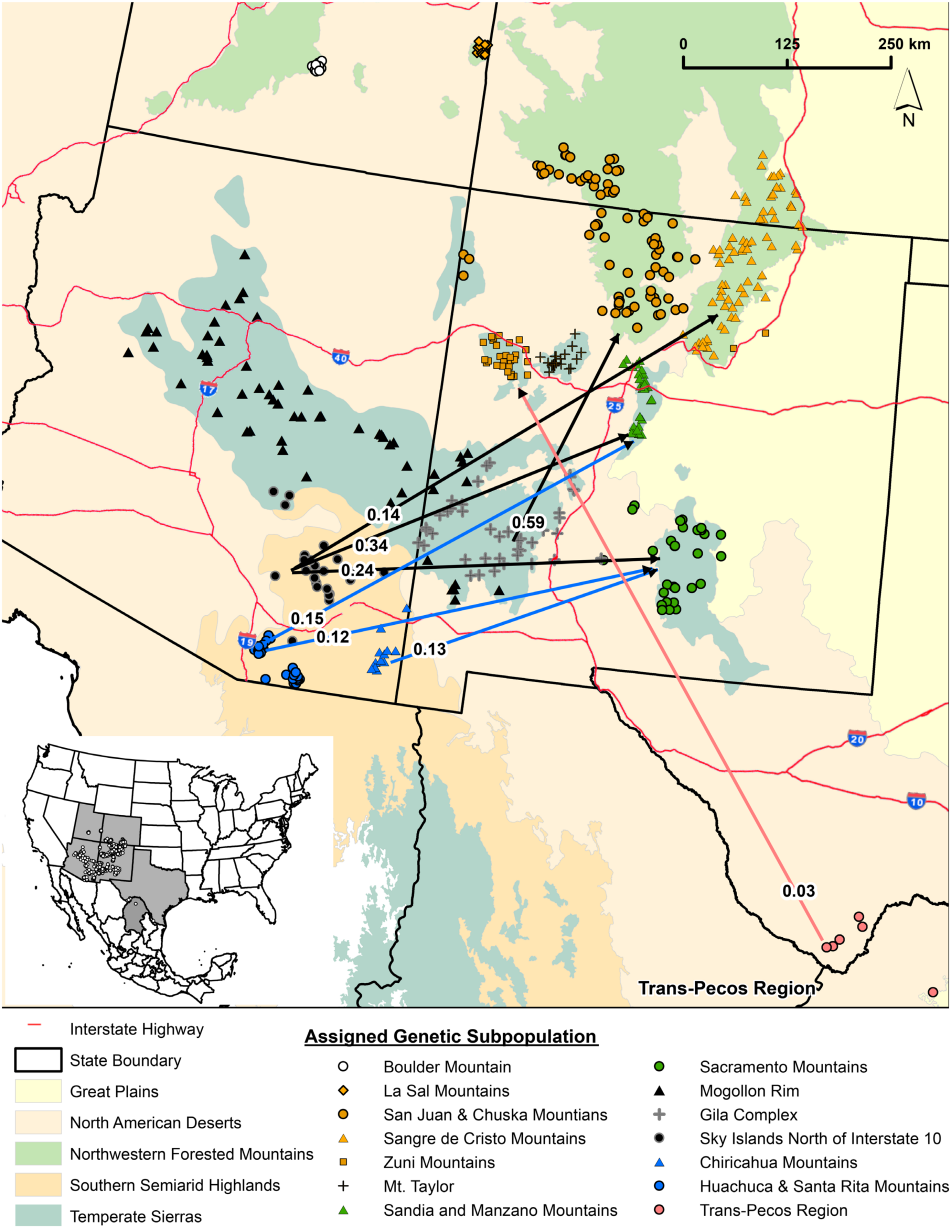
Directional relative migration network based on *G*_ST_ values for American black bear (*Ursus americanus*) subpopulations in the American Southwest and northern Mexico. The network visualized shows significant asymmetrical migration values for subpopulations identified using the correlated allele frequency model in program geneland.

The directional relative migration network‐clustered populations in the northern part of our study region along the Colorado–New Mexico border (ECPSRM) with populations located in the central portions of our study region, in the states of Arizona and New Mexico (DMS and MHSS), suggesting high rates of gene flow among these regional subpopulations. Estimated gene flow among the remaining subpopulations was low as most of the pairwise‐relative migration values (87%) were half of that occurring between the highest values from central Arizona and western New Mexico (DMS) to the Colorado–New Mexico border (ECPSRM; Figure 2; Appendix S3: Figure S1; Appendix S3: Table S7). There was a pronounced south‐to‐north linkage pattern of asymmetric gene flow from central Arizona and western New Mexico (DMS) to the Colorado–New Mexico border (ECPSRM) and central New Mexico (MHSS), from southern Arizona (SIS) to central New Mexico (MHSS), and from Texas and northern Mexico (TP) to the Colorado–New Mexico border (ECPSRM; Figure 2; Appendix S3: Figure S1; Appendix S3: Table S7). The mountain range subpopulations exhibited a similar pattern with subpopulations from the central portion of the study area clustering together and asymmetric gene flow in a northward direction (Figures 1 and 3; Appendix S4: Figure S1; Appendix S4: Table S11).

## 4 DISCUSSION

Our study further supports the hypothesis that the Southwest served as a fourth Pleistocene refugium for the American black bear during the LGM and that their present‐day genetic structure is most likely a result of vicariant events as habitat fragmentation occurred when glaciers receded post‐LGM. The Trans‐Pecos population originated from the Sierra Madre Oriental, a north to south running mountain range in northeast Mexico, and is more closely related to the eastern lineage of black bears that occupied the American Southeast refugium (Onorato, Hellgren, Van Den Bussche, & Doan‐Crider, 2004). The other more westerly populations would have likely arisen from the Sierra Madre Occidental, a parallel mountain range in western Mexico that is separated from the Sierra Madre Oriental by the Chihuahuan Desert (Varas‐Nelson, 2010). Our sampled populations were highly structured with those from central Arizona, central New Mexico, and southern Colorado clustering together; these populations were distinct but related to populations within the Sky Islands border region and all of these populations were distinct from Trans‐Pecos and Boulder Mountain. Populations would be expected to show genetic structure if repeated episodes of isolation and admixture occurred, driven by changes in habitat distribution.

### 4.1 The influence of forest refugia on genetic structure

Our analyses supported the hypothesis that the current genetic structure is a consequence of Pleistocene–Holocene vicariance whereby bears occupied forest refugia during the LGM, but then followed changes in the distribution of forests as the Holocene dried and warmed (Pleistocene–Holocene Vicariance Hypothesis). The Southwest likely served as a refugium for black bears during various periods in the Pleistocene and Holocene when habitats in more northerly latitudes were dominated by more cold‐adapted plant species that black bears do not typically use (Betancourt et al., 1990). Paleoecological reconstruction reveals considerable forest habitat available to black bears throughout the Southwest. This forest was widespread and found throughout lower elevation areas in what is currently Chihuahuan and Sonoran desert. In certain areas, these forested habitats were stable for 10,000–20,000 years, and were often found at lower elevations than they are today (Holmgren et al., 2006; McAuliffe & Van Devender, 1998; Van Devender, 1990a, 1990b). As climates aridified, forest habitats either moved up in elevation, moved north dependent upon precipitation patterns, soil moisture regimes, and winter temperatures, or both. Evidence of these expansion and isolation events can also be found in the fossil record. Fossil specimens identified as modern day black bear have been discovered at 12 relatively low‐elevation Pleistocene sites (mean = 1495 m; range = 1171–1716 m) dated to the mid‐ and late‐Wisconsin age (~11,000–65,000 BP) within the present day Chihuahuan and Sonoran deserts (Harris, 1987, 1989, 1993, 2003; Messing, 1986; Saunders, 1977; Skinner, 1942; Slaughter, 1975). Thus, for much of the late Pleistocene and into the early Holocene, the dominant paleovegetation community of the region was a piñon‐juniper‐oak woodland that black bears inhabited, similar to the plant community selected by black bears in the Sky Islands today (Onorato et al., 2003). The isolated Sky Island mountain ranges, currently inhabited by black bears, were most likely functionally connected by this piñon‐juniper‐oak woodland (Van Devender, 1990a).

Black bears are omnivorous, but vegetation, fruits, and nuts comprise 70–90% of the diet, supplemented with insects and vertebrates (Delgadillo Villalobos et al., 2019). In spring, they feed on grasses and other vegetation, in mid‐late summer on soft mast, such as berries, and in late summer–fall prior to hibernation they forage on hard mast, such as acorns and piñon pine nuts (Beck, 1991; Costello et al., 2001; Onorato et al., 2003). In lower elevations within the Southwest, they also feed on sotol (*Dasylirion* spp.), yucca (*Yucca* spp.), and prickly pear cactus (*Opuntia* spp.; Delgadillo Villalobos et al., 2019). Although found in semiarid shrublands, black bears are primarily a forest‐adapted species and forests are important habitats across their range (Evans et al., 2017; Gould et al., 2019; Onorato et al., 2003). Thus, it stands to reason that black bears would track the abundance of their main food over the short term, which would explain contemporary movements and dispersal patterns and populations would track the distribution of their primary habitat over the long term, which would explain species distribution and population genetic structure.

### 4.2 The influence of transportation infrastructure on genetic structure and gene flow

Our analyses did not support the hypothesis that interstate highways are limiting the movement of black bears across the Southwest (Anthropocene Filter Hypothesis). The uncorrelated allele frequency model failed to detect such a genetic pattern at the regional level and the correlated frequency model often clustered bears together that were on opposite sides of major interstates. For example, bears from the Mogollon Rim (MR) population in Arizona were found on both sides of Interstates 17 and 40; bears from the Zuni Mountains (ZM) population in New Mexico also clustered together from both sides of Interstate 25 and 40; bears from the Sandia and Manzano mountains (SMM) population in New Mexico were found on both sides of Interstate 40; bears from the Gila complex (GC) in New Mexico were found on both sides of Interstate 25, although primarily to the west; and bear populations from the Sky Islands North of Interstate 10 (SIN) and from the Chiricahua complex (CHC) of Arizona were found on both sides of Interstate 10.

Roads, urbanization, and interstate highways can negatively influence carnivore populations and the size of the interstate (e.g., number of traffic lanes) and relative traffic flow may be contributing factors as well (Riley et al., 2014; Serieys et al., 2015). Perhaps one of the most extreme cases has occurred in the Santa Monica Mountains of southern California where the morass of urbanization and grand thoroughfares has restricted population size and caused degradation in genetic variation in mountain lions (*Puma concolor*; Riley et al., 2014). Although bear resource use is negatively affected by roads (Gould et al., 2019), the effect of roads on bear movements and gene flow varies across their range. Roads had little effect on movements in remote areas such as in Idaho (Cushman et al., 2006) but had major impacts on movement patterns and genetic structure in more heavily urbanized areas such as Florida (Dixon et al., 2007; McCown et al., 2009). The highways in the Southwest receive less traffic volume and are narrower than heavily populated areas like California or Florida, so their impedance to bear movement would be expected to be reduced. Riparian underpasses could also focus movement across Southwest highways as bears are less likely to traverse the desert scrub matrix and are more likely to be close to streams (Jensen et al., 2022). Furthermore, black bears have a relatively long generation time and interstate highways are recent, anthropogenic barriers or filters, and their current genetic structure may not reflect the impact of interstate highways as there has been insufficient time for populations to diverge among those bisected by interstates (Blair et al., 2012; Epps et al., 2005; Safner et al., 2011).

A handful of observed long‐distance and cross‐interstate movements by bears supports the hypothesis that interstates in the Southwest are not yet a barrier to bear movement and thus have most likely not influenced gene flow. We genotyped five individuals (three males and two females) that we identified as being approximately 90 km, 150 km, 300 km, and 360 km away from where they were originally captured, collected, or detected by collaborating agencies. These observed movements required the individuals to cross Highway 70, Interstate 25, or Interstate 40. Finally, Liley and Walker (2015) placed a GPS collar on a male bear on the New Mexico–Colorado border that subsequently traveled to central Colorado and crossed Interstate 25 twice before returning to New Mexico, a cumulative distance of 1482 km. These observations show that bears in the Southwest can travel long distances and cross both highways and interstates when doing so.

### 4.3 The scale of population genetic structure in Southwestern black bear populations

Regionally, subpopulation boundaries followed the distribution of three major ecoregions (Omernik & Griffith, 2014): the Northwestern Forested Mountains contained populations in the Eastern Colorado Plateau and Southern Rocky Mountains (ECPSRM) and Boulder Mountain, Utah (BM); the Temperate Sierras harbored bears from the Datil‐Mogollon Section (DMS) and the Mexican Highlands and Sacramento Section (MHSS); and the Southern Semi‐Arid Highlands contained populations from the Sky Islands South of Interstate 10 (SIS). Low genetic differentiation and relatively high gene flow among the three largest subpopulations suggest these subpopulations (ECPSRM, DMS, and MHSS) form the core contemporary Southwest black bear population. The SIS in southern Arizona shows moderate differentiation from this core population, which is surprising given their proximity (distance between the SIS and the DMS is ~20 km) and is likely due to the relatively inhospitable habitat matrix separating the Sky Islands region from other subpopulations. The Sky Islands are a series of “montane islands separated by a desert sea” where the intervening landscape matrix of primarily Chihuahuan or Sonoran desert acts as a semi‐permeable barrier to black bear dispersal and gene flow (Lomolino et al., 1989). Atwood et al. (2011) also found genetic substructure among black bear populations along the US–Mexico border within the Sky Islands region.

There was a relatively high degree of genetic differentiation when comparing Boulder Mountain, Utah and the Trans‐Pecos region in west Texas and northeast Mexico to the other Southwest subpopulations. We believe this differentiation is due to genetic isolation rather than incomplete sampling. The origination of the Boulder Mountain population is more enigmatic and more information is needed to determine if it is a product of eastward expansion by populations from the Pacific Northwest refugium or through the expansion and isolation of populations from the north (Lackey et al., 2013; Malaney et al., 2018; Puckett et al., 2015).

We had a small sample from the Trans‐Pecos region, but that subpopulation had the highest allelic richness, the highest observed heterozygosity, many private alleles, and some private alleles occurred at high frequency, indicating a period of isolation and genetic differentiation followed by little to no connectivity (Slatkin, 1985). The existence of private alleles in the Trans‐Pecos region is most likely a product of isolation followed by bears recolonizing west Texas from the Sierra Madre Oriental (Onorato et al., 2007; Onorato, Hellgren, Van Den Bussche, & Doan‐Crider, 2004). Bears in the Sierra Madre Occidental are genetically distinct from those in the Sierra Madre Oriental (Varas‐Nelson, 2010). The high levels of genetic diversity may partly be a product of migration‐dispersal events due to hard mast crop failure in Big Bend National Park, Texas. Those events resulted in the movement of bears back to the Sierra del Carmen, Mexico, and invariably subsequent movements back to west Texas (Onorato et al., 2003; Onorato, Hellgren, Van Den Bussche, & Doan‐Crider, 2004).

The unique genetic variation of the Trans‐Pecos subpopulation also reflects the ancestral relationship between the eastern Mexican and eastern American black bear populations that are hypothesized to have occupied the American Southeast refugium before diverging 67–31 kya (Pedersen et al., 2021, Van Den Bussche et al., 2009). Pedersen et al.'s (2021) hypothesis that gene flow between black bear populations in the Sierra Madre Occidental and Oriental was inhibited by the Chihuahuan Desert conflicts with paleomidden evidence that shows pygmy conifer woodlands dominated the present‐day Chihuahuan Desert during the Pleistocene (Betancourt et al., 1990). Furthermore, black bear fossils have been discovered at low‐elevation Pleistocene caves dated to ~11,000–65,000 BP within the present‐day Chihuahuan Desert (Harris, 1987, 1989, 1993, 2003; Messing, 1986; Saunders, 1977; Slaughter, 1975). These caves could be sampled for ancient environmental DNA or ancient DNA could be amplified from the fossils themselves to further our understanding of the distribution of refugia and the movements and genetic structure of bears in the American Southwest and northern Mexico.

### 4.4 The influence of landscape resistance and geographic distance on gene flow

Our estimates of the relative degree and direction of gene flow also suggested that gene flow occurred from south to north and was high among the regionally central subpopulations where contiguous forest existed. Limited gene flow among these central subpopulations and the southern Madrean Sky Island Archipelago appeared to be filtered by the mosaic of less hospitable habitat found in the lowlands. The relative degree of gene flow was also affected by the geographic distance among subpopulations. The Boulder Mountain, Utah population (BM) and the Trans‐Pecos population in west Texas and northeast Mexico (TP) were isolated from the other populations and showed little gene flow with them. This pattern was not unexpected as ~200 km of the Colorado Plateau and ~430 km of the Chihuahuan Desert separates the BM and TP populations from their nearest subpopulation, respectively.

The consistent pattern of asymmetric gene flow northward is indicative of prehistoric range expansion. Varas‐Nelson (2010) noted a similar pattern in northern Mexico where the migration rate from Sierra El Nido in Sonora to Sierra San Luis in Chihuahua, ~250 km to the north, was 2.5× greater than the migration rate southward. Northward expansion also supports previous research that postulated that the Southwest refugium dominated the genetic assemblage of the Intermountain West before admixing along the US.–Canada border with bears that originated from the Great Lakes region (Pelletier et al., 2011; Puckett et al., 2015).

Our resistance‐based models of gene flow revealed that areas with higher canopy cover and precipitation, essentially forested habitats, were associated with higher rates of gene flow and within these areas, gene flow was facilitated by less rugged areas. Gene flow was also affected by geographic distance. Thus, populations connected by contiguous forest had high gene flow (e.g., Eastern Colorado Plateau and Southern Rocky Mountains, Datil‐Mogollon Section, Mexican Highlands, and Sacramento Section) and populations separated by desert and isolated by distance had lower gene flow (e.g., Trans‐Pecos and Boulder Mountain). Because female black bears are highly philopatric, the effect of distance on gene flow may be governed by their behavior, but also mediated by long‐distance dispersal events by male bears with male bears also having been shown to select against ruggedness (Apps et al., 2006; Johnson et al., 2015; Lara‐Díaz et al., 2018; Pelletier et al., 2011). So, it appears that habitat most likely acts as a conduit (e.g., forest) or filter (e.g., desert) to bear movement and that geographic distance plays an important role owing to intersexual differences in movement behavior.

### 4.5 Conservation implications

American black bears in the Southwest occupy a naturally fragmented landscape with low‐density subpopulations linked together into a metapopulation (Gould et al., 2018; Onorato, Hellgren, Van Den Bussche, & Doan‐Crider, 2004). Habitat loss and fragmentation owing to climate change, anthropogenic land use, and US–Mexico border security could increase the extinction risk of individual subpopulations and sever linkages among key subpopulations within the metapopulation (Lara‐Díaz et al., 2021).

Climate change is contributing to a rise in aridity and temperature in the Southwest and has led to increases in insect outbreaks, intense droughts, and catastrophic wildfires resulting in substantial tree mortality reducing the distribution and quality of bear habitat over the long term (Gould et al., 2019; Thorne et al., 2018; Williams et al., 2010). Increasing human development and population growth is likely to increase human population density and traffic rates resulting in higher rates of road mortality, which could lower genetic connectivity and enhance fragmentation, heightening the extinction risk for some subpopulations (Dixon et al., 2007; Ernest et al., 2014; Riley et al., 2014). Finally, the US.–Mexico border wall poses a threat to the persistence of bears in the Southwest. The current border wall spans ~1125 km, and in the recent past, the US government proposed to increase the length of the border wall and change vehicle barriers that are permeable to bears, to impassable pedestrian barriers that would impede cross‐border migration and dispersal (4–10 m tall, 5–10 cm wide gaps; Flesch et al., 2010). The current wall and more impenetrable barriers could sever linkages between populations in the Sierra Madre Occidental of northern Mexico with those in southern Arizona and New Mexico (Atwood et al., 2011; Varas‐Nelson, 2010) and the Sierra Madre Oriental of northern Mexico with those in west Texas (Hellgren et al., 2005; Onorato, Hellgren, Van Den Bussche, & Doan‐Crider, 2004). Binational collaboration between the United States and Mexico could be crucial to the future persistence and viability of the black bear metapopulation in southwestern North America and represents a unique conservation opportunity.

## AUTHOR CONTRIBUTIONS

**Matt Gould:** Conceptualization (equal); formal analysis (equal); methodology (equal); writing – original draft (equal); writing – review and editing (equal). **James w Cain III:** Conceptualization (equal); funding acquisition (equal); writing – original draft (equal); writing – review and editing (equal). **Todd Atwood:** Writing – review and editing (equal). **Larisa Harding:** Writing – review and editing (equal). **Heather E. Johnson:** Writing – review and editing (equal). **Dave P Onorato:** Writing – review and editing (equal). **Frederic Winslow:** Writing – review and editing (equal). **Gary Roemer:** Conceptualization (equal); funding acquisition (equal); methodology (equal); writing – original draft (equal); writing – review and editing (equal).

## CONFLICT OF INTEREST

None declared.

## Supporting information


Appendix S1–S5

## Data Availability

Microsatellite data used in this study are available through USGS ScienceBase, https://doi.org/10.5066/P91COLPR.
