# Supplementary material for: Pleistocene–Holocene vicariance, not Anthropocene landscape change, explains the genetic structure of American black bear (Ursus americanus) populations in the American Southwest and northern Mexico
Source: Ecol Evol. 2022 Oct 10;12(10):e9406. doi: 10.1002/ece3.9406 (PMC9551525; doi:10.1002/ece3.9406)
Supplement: Supplementary file 1 — Appendix S1–S5 [file ECE3-12-e9406-s001.docx]

**Supplemental Information for:**

**Pleistocene-Holocene vicariance not Anthropocene landscape change, explains the genetic structure of American black bear (*Ursus americanus*) populations in the American Southwest and northern Mexico**

Matthew J. Gould^1, 2,^ †, James W. Cain, III^1, 2, 3^, Todd C. Atwood^4^, Larisa E. Harding^5^, Heather E. Johnson^4^, Dave P. Onorato^6^, Frederic S. Winslow^7^, Gary W. Roemer^1, 2, δ^

^1^ Department of Fish, Wildlife and Conservation Ecology, New Mexico State University, Las Cruces, NM 88003, USA

^2^ Department of Biology, New Mexico State University, Las Cruces, NM 88003, USA

^3^ U.S. Geological Survey New Mexico Cooperative Fish and Wildlife Research Unit, New Mexico State University, Las Cruces, NM 88003, USA

^4^ U.S. Geological Survey, Alaska Science Center, Anchorage, AK 99508, USA

^5^ Arizona Game and Fish Department, Phoenix, AZ 85086, USA

^6^ Fish and Wildlife Research Institute, Florida Fish and Wildlife Conservation Commission, Naples, Florida 34114, USA

^7^ New Mexico Department of Game and Fish Santa Fe, NM 87507 USA

Corresponding author: mjgould4@gmail.com

Table of Contents

[Appendix S1. Sampling and STRUCTURE Analysis 3](#_Toc108956749)

[METHODS 3](#_Toc108956750)

[RESULTS 3](#_Toc108956751)

[Figure S1 6](#_Toc108956752)

[Figure S2 7](#_Toc108956753)

[Figure S3 8](#_Toc108956754)

[Figure S4 9](#_Toc108956755)

[Figure S5. 10](#_Toc108956756)

[Figure S6 11](#_Toc108956757)

[Table S1 12](#_Toc108956758)

[Table S1 continued 13](#_Toc108956759)

[Table S2 14](#_Toc108956760)

[Table S2 continued 16](#_Toc108956761)

[Table S6 18](#_Toc108956762)

[Table S4 19](#_Toc108956763)

[Table S6 20](#_Toc108956764)

[Table S6 21](#_Toc108956765)

[Table S7 22](#_Toc108956766)

[Table S8 23](#_Toc108956767)

[Table S9 24](#_Toc108956768)

[Table S10 25](#_Toc108956769)

[Table S11 26](#_Toc108956770)

[Table S12 27](#_Toc108956771)

[Table S13 33](#_Toc108956772)

[Table S14 34](#_Toc108956773)

[Appendix S2. Global results 35](#_Toc108956774)

[Table S1 35](#_Toc108956775)

[Table S2 36](#_Toc108956776)

[Table S3 37](#_Toc108956777)

[Table S4 38](#_Toc108956778)

[Appendix S3. Regional subpopulation results 39](#_Toc108956779)

[Figure S1 39](#_Toc108956780)

[Table S1 40](#_Toc108956781)

[Table S2 42](#_Toc108956782)

[Table S3 43](#_Toc108956783)

[Table S4 44](#_Toc108956784)

[Table S5 45](#_Toc108956785)

[Table S6 46](#_Toc108956786)

[Table S.7 51](#_Toc108956787)

[Appendix S4. Mountain range subpopulation results 52](#_Toc108956788)

[Figure S1 52](#_Toc108956789)

[Table S1 53](#_Toc108956790)

[Table S1 continued 55](#_Toc108956791)

[Table S2 57](#_Toc108956792)

[Table S3 58](#_Toc108956793)

[Table S4 59](#_Toc108956794)

[Table S5 60](#_Toc108956795)

[Table S6 61](#_Toc108956796)

[Table S7 62](#_Toc108956797)

[Table S8 63](#_Toc108956798)

[Table S9 64](#_Toc108956799)

[Table S10 65](#_Toc108956800)

[Table S.11 71](#_Toc108956801)

[Appendix S5. Resistance surface optimization results 72](#_Toc108956802)

[Table S1 72](#_Toc108956803)

[Table S2 74](#_Toc108956804)

## Appendix S1. Sampling and STRUCTURE Analysis

### **METHODS**

We estimated population structure using the aspatial Bayesian clustering program, structure (v2.3.4; Pritchard, Stephens, & Donnelly, 2000). We conducted 10 independent runs per K, varied K from 1 to 31 (number of sampling locations + 1), and used 100,000 burn-in steps followed by 200,000 Markov Chain Monte Carlo iterations under the admixture model with correlated allele frequencies (Falush, Stephens, & Pritchard, 2003). We used structure harvester (Earl & vonHoldt, 2012) to evaluate the optimum K using a combination of the mean log likelihood, LnP(K), and the ΔK method (Evanno, Regnaut, & Goudet, 2005; Pritchard et al., 2000). We used clumpp to average individual Q-values (i.e., proportion of ancestry belonging to each K) and to deal with label switching among the 10 independent runs (Jakobsson & Rosenberg, 2007). We assigned individuals to a cluster based on their highest Q-value. We investigated hierarchical structure by repeating the structure analysis iteratively for each identified K until K = 1 (Coulon et al., 2008).

We tested for linkage disequilibrium (LD), null alleles, and deviations from Hardy-Weinberg proportions (HW) using the R package popgenreport v3.0.0 (Adamack & Gruber, 2014). We applied a Bonferroni correction of α < 0.0005 (LD) and α < 0.003 (HWE) to reduce the likelihood of a false positive significance test, and we used popgenreport to generate summary statistics for each locus. With individuals grouped into the K clusters, we quantified genetic diversity using unadjusted private alleles (A_P_) and private alleles using rarefaction (A_PR_), accounting for differences in sample size among subpopulations, using hp-rare v1.0 (Kalinowski, 2004, 2005). We also quantified genetic diversity using expected (H_E­_) and observed (H_O_) heterozygosity and allelic richness using rarefaction (A_R_), using the r package diversity (version 1.9.9; Keenan, McGinnity, Cross, Crozier, & Prodöhl, 2013). We also used diversity to estimate an inbreeding coefficient (F_IS_) and its 95% confidence interval (CI) based on 1,000 bootstrap iterations. We calculated genetic differentiation among the genetic clusters (F_ST_) in popgenreport and classified values of 0.05–0.14, 0.15–0.24, and ≥0.25 as moderate, high, and very high differentiation, respectively (Hartl & Clark, 1997).

We investigated asymmetric gene flow by estimating relative migration among the estimated K clusters using divmigrate in the R package diversity where maximum relative gene flow is set at 1 and minimum at 0 (Keenan et al., 2013; Sundqvist, Keenan, Zackrisson, Prodöhl, & Kleinhans, 2016). We calculated Gst for network plots, conducted 1,000 bootstrap iterations to generate 95% CIs to evaluate if asymmetric gene flow was significant, and chose to display connections ≥ 0.50. We implemented all other population genetics analyses in program R v3.5.2 (R Core Team, 2018).

### **RESULTS**

Based on ΔK, the initial analysis by structure supported K = 2 genetic clusters with a smaller peak at K = 7. The LnP(K), however, showed little support for K = 2 with LnP(K) increasing with each increment in K until it began to plateau at K = 7. Therefore, we chose K = 7 as the initial level of hierarchical structure (Appendix Figure A.1; Appendix Table A.1). The 7 clusters followed a similar geographic distribution as those identified in our geneland analysis (K = 6) except bears in the Sangre de Cristo Mountains were designated as their own cluster (Appendix S1: Figure S2). The structure analysis also split most of the Mt. Taylor bears off from the Southern Rockies and clustered them with bears in the Mogollon Rim, AZ and Gila, NM region. Our reanalysis of the 7 clusters for hierarchical structure identified further substructure totaling K = 13 genetic clusters (Appendix Figures A.2–A.5).

The presence of null alleles ranged considerably with 95% CIs not overlapping 0 for a range of loci across all clusters except clusters 5.2 and 6 (Appendix Table A.2). We found non-random associations (LD) between the G10U and G10B loci and between the G10X and G10L loci in cluster 3.2. We also found the CXX20 locus to be non-randomly associated with G10B and G10H in cluster 4 (Appendix Tables A.6–A.9). We found no loci to be out of HW proportions (Appendix Table A.10).

Allelic richness was lowest in the cluster 3.1 (2.88) and highest in the cluster 5.1 (4.54). Clusters 3.1, 5.2, and 7.1 had the fewest private alleles (A_PR_ = 0.02) while cluster 2.2 had the most with 0.63 (Appendix S1: Table S11; Appendix S1: Table S12). Observed heterozygosity ranged from 0.39–0.69 while H_E_ ranged similarly (0.39–0.64) (Appendix S1: Table S11). The F_IS_ estimates suggested potential inbreeding for clusters 1.2, 2.1, and 2.2 as their 95% CIs did not overlap 0, however, H_O_ was higher than H_E_ for all 3 clusters (Appendix S1: Table S11). Genetic differentiation was the lowest between clusters 5.1 and 5.2 (0.01) and highest between 2.1 and 3.1 (0.29). Overall, clusters 2.1, 2.2, and 4 displayed the highest levels of genetic differentiation when compared to all other clusters (Appendix S1: Table S13).

The directional relative migration network grouped the clusters in similar fashion as the mountain range analysis with Arizona, Colorado, and New Mexico clusters (1.1, 1.2, 1.3, 5.1, 5.2, 6, and 7.1) being grouped together. Bear subpopulations found in Boulder Mountain, the Sky Islands south of I-10, and Texas (4, 3.1, 3.2, 2.1, and 2.2, respectively) were grouped independent of each other on the periphery of the main cluster (Appendix S1: Figure S6; Appendix S1: Table S14). Only 1 comparison was ≥ 0.50, which occurred between clusters 5.2 to 5.1 (1 vs. 0.57; Appendix S1: Figure S6 panel b; Appendix S1: Table S14). The 95% CIs for the relative migration network indicated that asymmetric gene flow occurs from clusters from 5.2 to 5.1 (1 vs. 0.57), from 6 to 5.1 (0.26 vs. 0.13), and from cluster 3.1 to 1.3 (0.08 vs. 0.03), 5.1 (0.12 vs.0.04), 5.2 (0.08 vs. 0.03), 6 (0.11 vs. 0.04), 7.1 (0.08 vs. 0.03), and 7.2 (0.06 vs. 0.03; Appendix S1: Figure S6; Appendix S1: Table S14).

**LITERATURE CITED**

Adamack, A. T., & Gruber, B. (2014). PopGenReport: Simplifying basic population genetic analyses in R. *Methods in Ecology and Evolution*, *5*(4), 384–387. doi: 10.1111/2041-210X.12158

Coulon, A., Fitzpatrick, J. W., Bowman, R., Stith, B. M., Makarewich, C. A., Stenzler, L. M., & Lovette, I. J. (2008). Congruent population structure inferred from dispersal behaviour and intensive genetic surveys of the threatened Florida scrub-jay (*Aphelocoma cœrulescens*). *Molecular Ecology*, *17*(7), 1685–1701. doi: 10.1111/j.1365-294X.2008.03705.x

Earl, D. A., & vonHoldt, B. M. (2012). STRUCTURE HARVESTER: A website and program for visualizing STRUCTURE output and implementing the Evanno method. *Conservation Genetics Resources*, *4*(2), 359–361. doi: 10.1007/s12686-011-9548-7

Evanno, G., Regnaut, S., & Goudet, J. (2005). Detecting the number of clusters of individuals using the software structure: A simulation study. *Molecular Ecology*, *14*(8), 2611–2620. doi: 10.1111/j.1365-294X.2005.02553.x

Falush, D., Stephens, M., & Pritchard, J. K. (2003). Inference of population structure using multilocus genotype data: Linked loci and correlated allele frequencies. *Genetics*, *164*, 1567–1587.

Hartl, D. L., & Clark, A. G. (1997). *Principles of population genetics* (3rd ed.). Sinauer Associates, Inc, Sunderland, MA.

Jakobsson, M., & Rosenberg, N. A. (2007). CLUMPP: A cluster matching and permutation program for dealing with label switching and multimodality in analysis of population structure. *Bioinformatics*, *23*(14), 1801–1806.

Kalinowski, S. T. (2004). Counting alleles with rarefaction: Private alleles and hierarchical sampling designs. *Conservation Genetics*, *5*(4), 539–543. doi: 10.1023/B:COGE.0000041021.91777.1a

Kalinowski, S. T. (2005). hp-rare 1.0: A computer program for performing rarefaction on measures of allelic richness. *Molecular Ecology Notes*, *5*(1), 187–189. doi: https://doi.org/10.1111/j.1471-8286.2004.00845.x

Keenan, K., McGinnity, P., Cross, T. F., Crozier, W. W., & Prodöhl, P. A. (2013). diveRsity: An R package for the estimation and exploration of population genetics parameters and their associated errors. *Methods in Ecology and Evolution*, *4*(8), 782–788. doi: 10.1111/2041-210X.12067

Pritchard, J. K., Stephens, M., & Donnelly, P. (2000). Inference of population structure using multilocus genotype data. *Genetics*, *155*(2), 945–959.

R Core Team. (2018). *R: A language and environment for statistical computing.* R Foundation for Statistical Computing, Vienna, Austria. Retrieved from http://www.R-project.org/.

Sundqvist, L., Keenan, K., Zackrisson, M., Prodöhl, P., & Kleinhans, D. (2016). Directional genetic differentiation and relative migration. *Ecology and Evolution*, *6*(11), 3461–3475. doi: 10.1002/ece3.2096


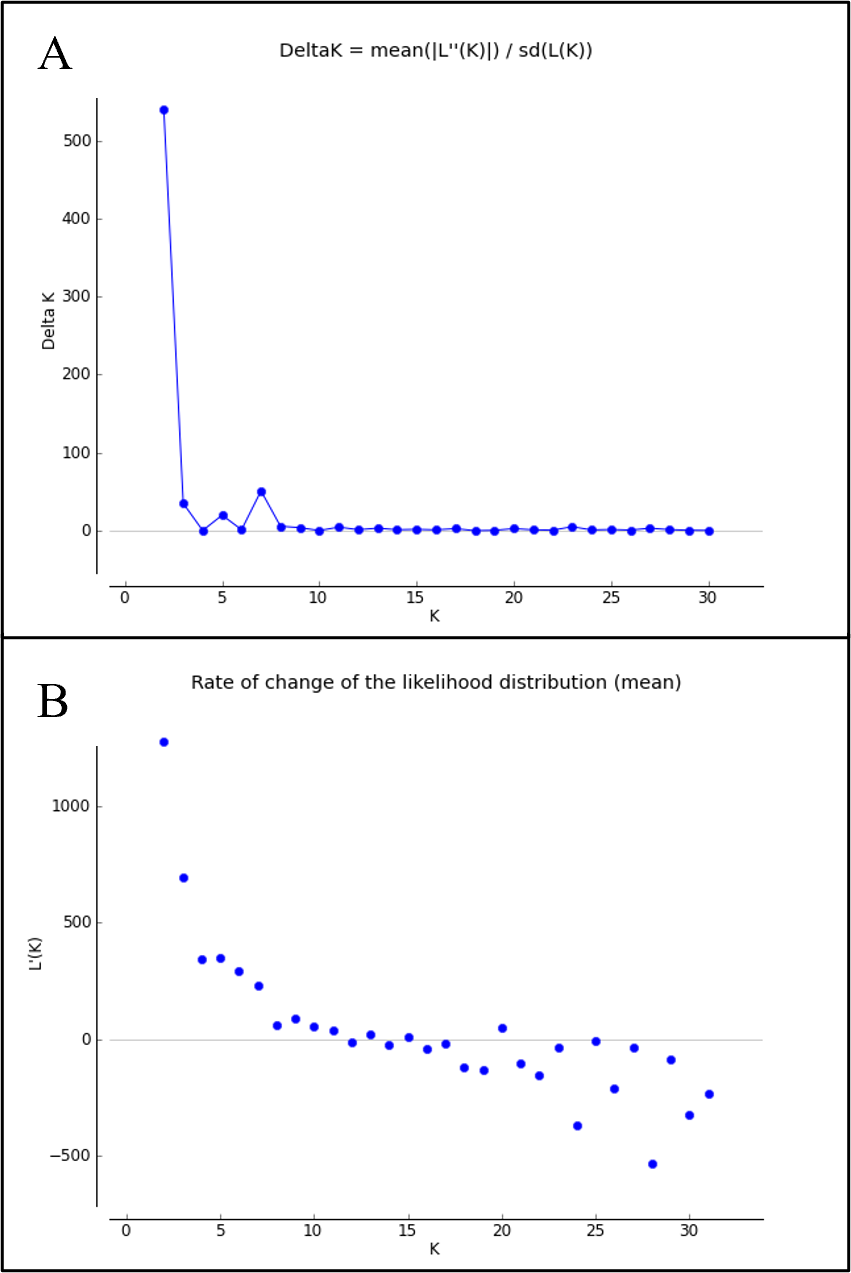


Figure S1. The optimum number of genetic clusters (K) identified by program structure using a combination of the (A) ΔK method (B) and the mean log likelihood, LnP(K), for the global population of American black bears (*Ursus americanus*) in the American Southwest and northern Mexico.


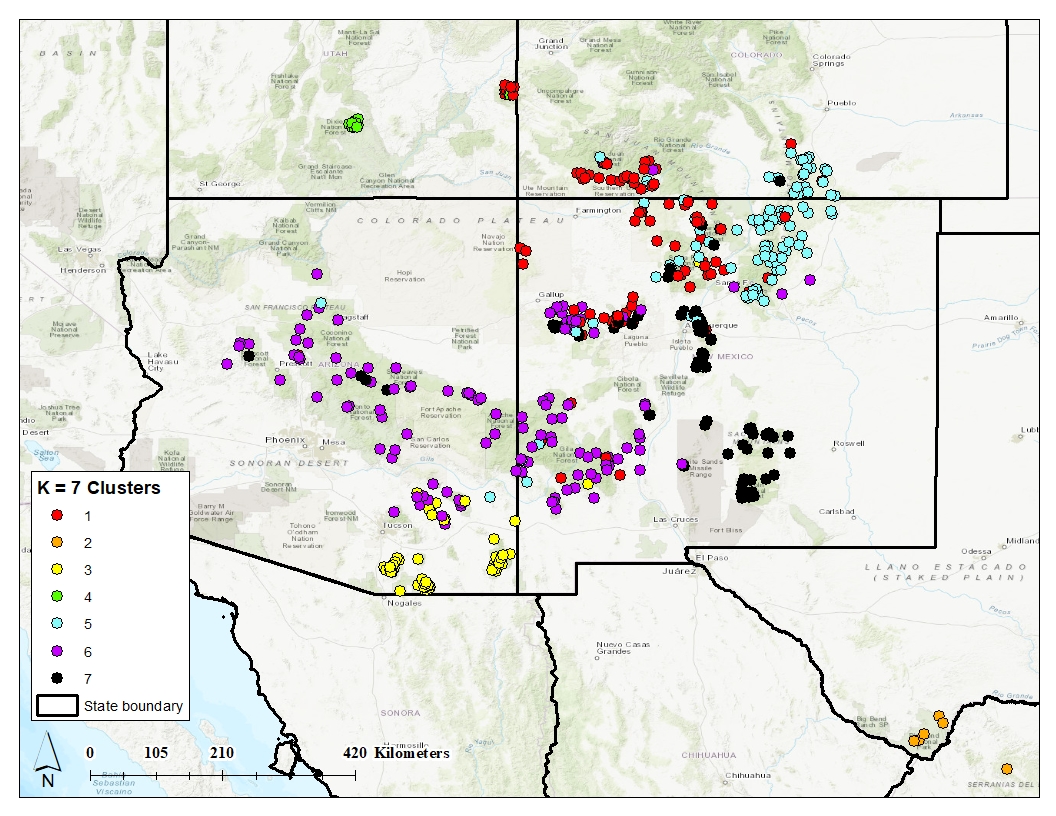

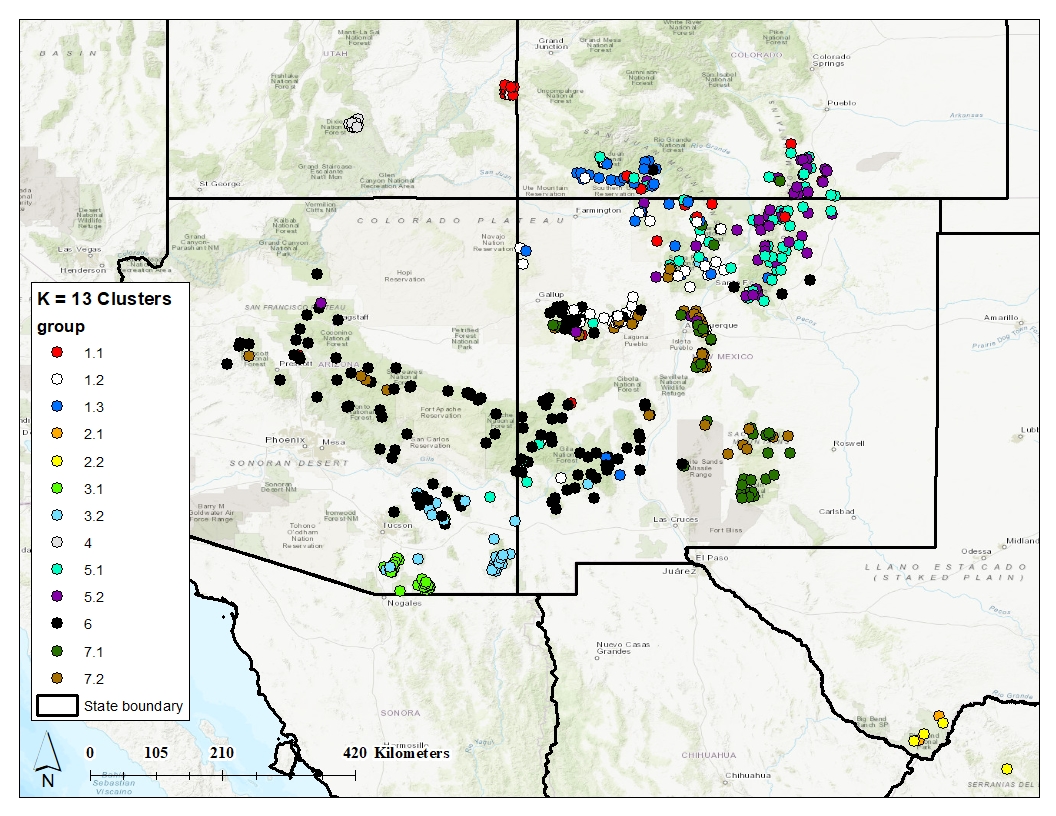


B

A

Figure S2. A) Highest hierarchical levels of genetic clusters identified by program structure and B) genetic clusters identified by program structure after accounting for hierarchical structure for American black bears (*Ursus americanus*) in the American Southwest and northern Mexico. We investigated hierarchical structure by splitting each identified cluster until K = 1.


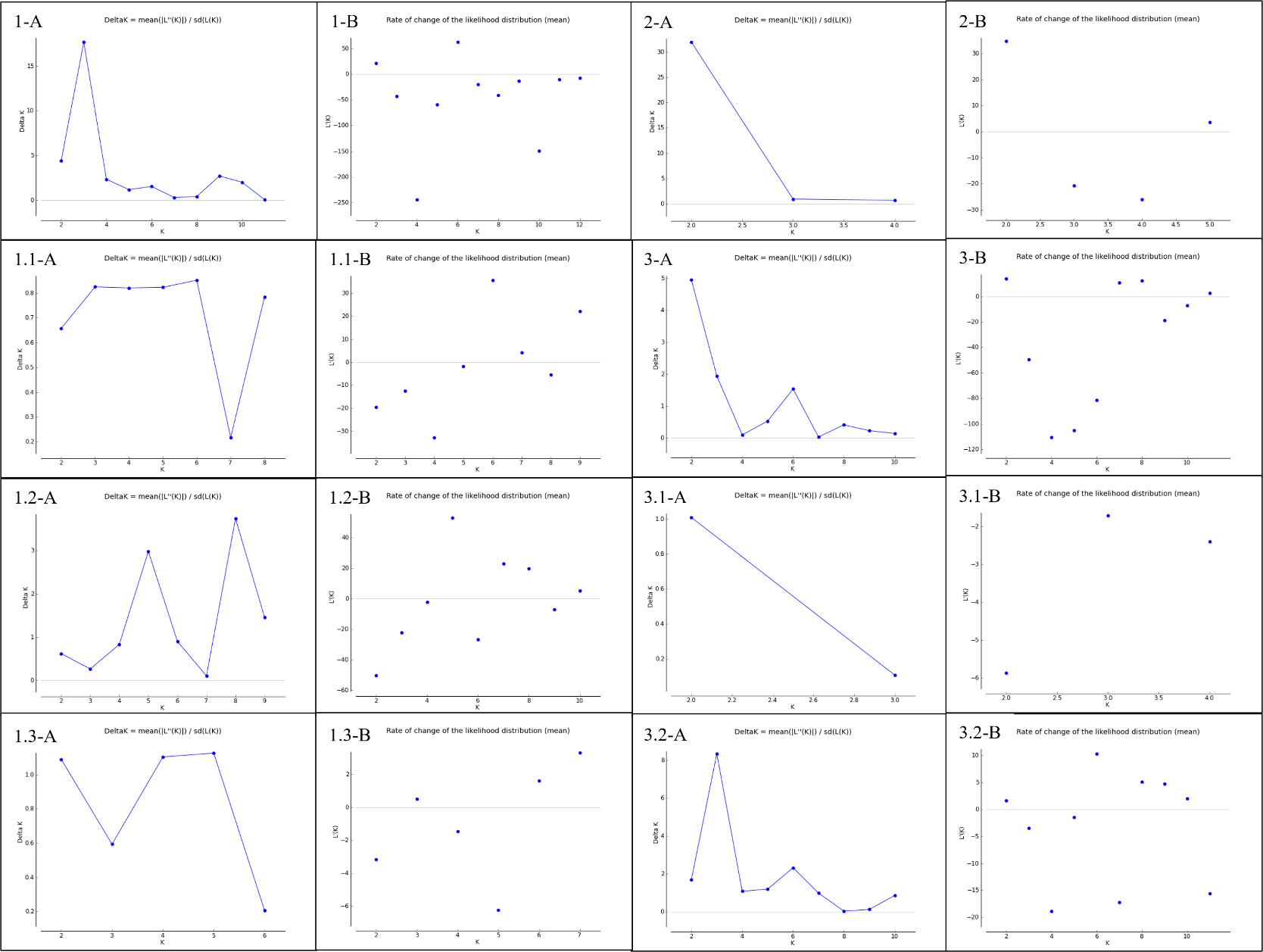


Figure S3. The optimum number of genetic clusters (K) using a combination of the (A) ΔK method (B) and the mean log likelihood, LnP(K), for genetic clusters 1–3. We investigated hierarchical structure for American black bears (*Ursus americanus*) in the American Southwest and northern Mexico by splitting each cluster identified by structure until K = 1.


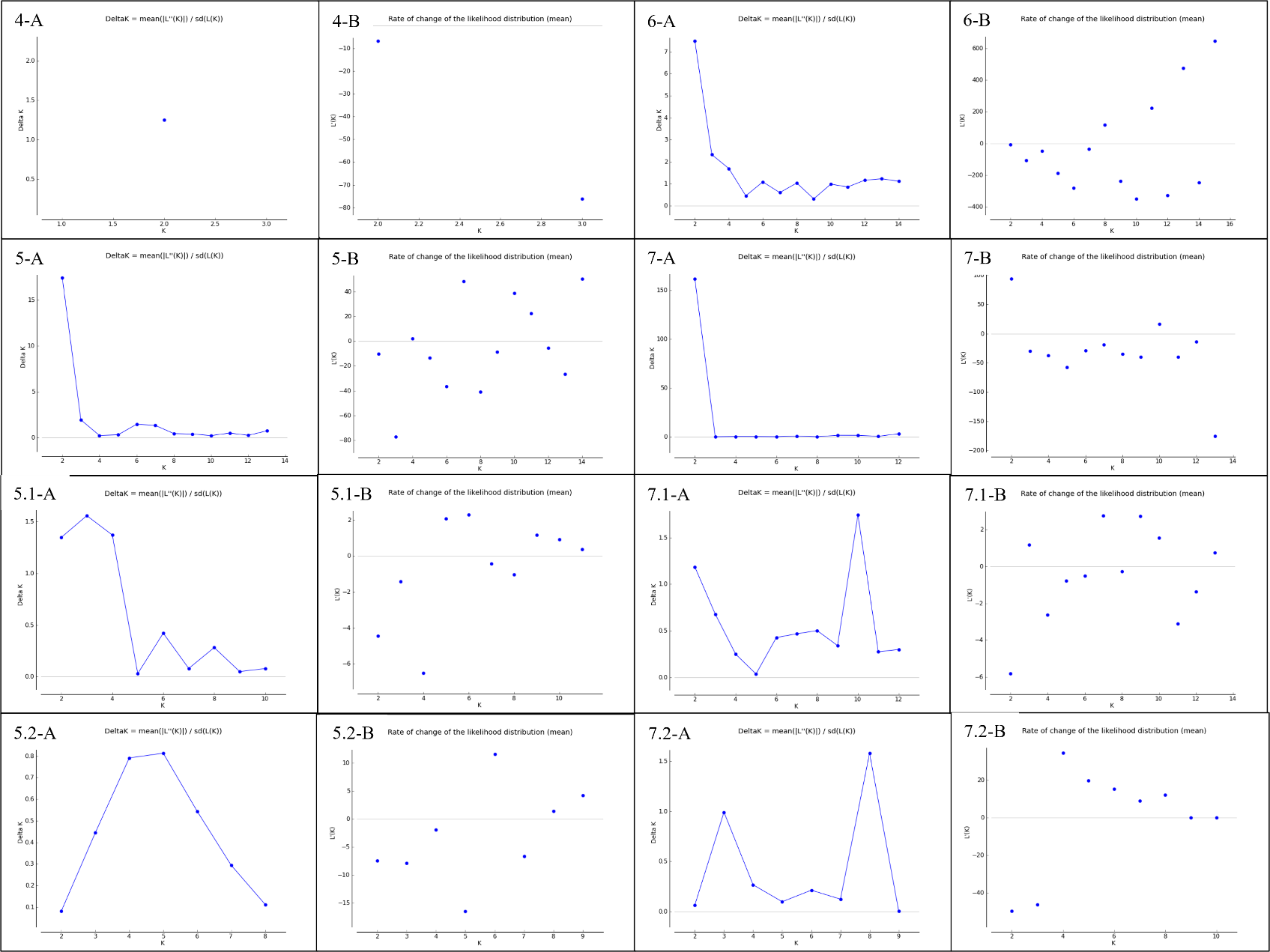


Figure S4. The optimum number of genetic clusters (K) using a combination of the (A) ΔK method (B) and the mean log likelihood, LnP(K), for genetic clusters 4–7. We investigated hierarchical structure for American black bears (*Ursus americanus*) in the American Southwest and northern Mexico by splitting each cluster identified by structure until K = 1.


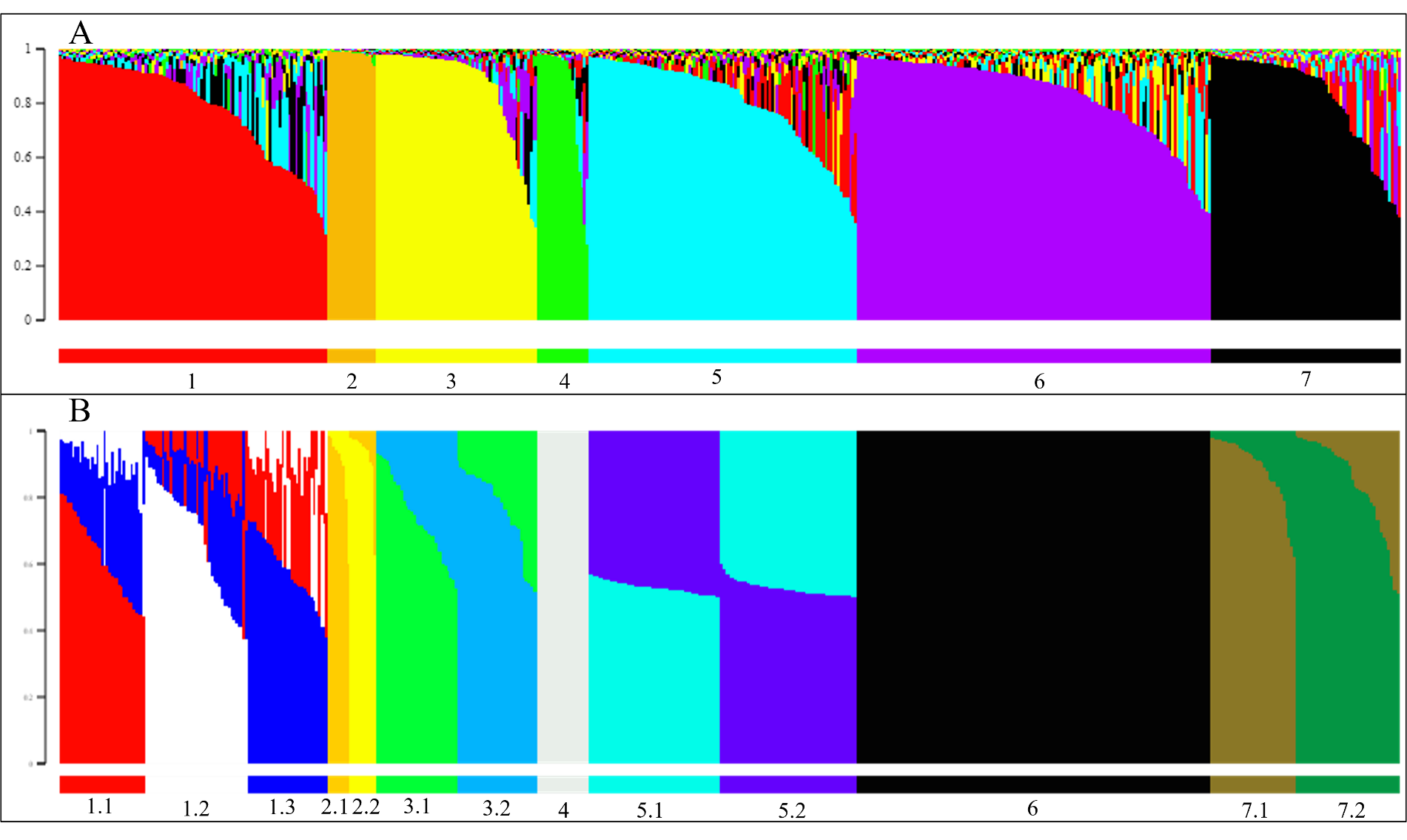


Figure S5. Proportional membership (Q) of individual American black bears for the (A) highest hierarchical levels of genetic clusters identified by program structure and (B) after accounting for hierarchical structure for American black bears (*Ursus americanus*) in the American Southwest and northern Mexico. Individual American black bears are represented by a single vertical bar. The genetic cluster assigned to each individual bear is indicated by the numbers at the bottom of each plot and correspond with the genetic clusters in Figure S2.

Figure S6. Directional relative migration networks based on G_ST_ for American black bear (*Ursus americanus*) clusters in the American Southwest and northern Mexico. Networks visualized include (a) all migration values (b) migration values ≥ 0.50, and (c) significant asymmetrical migration values.

Table S1. Justification for splitting clusters identified by structure when investigating hierarchical structure for American black bears (*Ursus americanus*) in the American Southwest and northern Mexico. We split each identified cluster until K = 1.

| Cluster | | K tested | K selected | Cluster location | Justification for K |
| --- | --- | --- | --- | --- | --- |
| Global | 31 | | 7 | Global | ΔK was highest at K = 2 with another small peak at K = 7. LnP(K) showed an increasing value for K until it plateaued at 7. K = 7 selected. |
| 1 | 12 | | 3 | La Sal, San Juan, | ΔK was highest at K = 3. LnP(K) was highest at K = 2, but K = 3 was close. K = 3 chosen |
|  |  |  |  | Mt. Taylor Region |  |
| 1.1 | 9 | | 1 | La Sal Mountains | ΔK was highest at K = 6, however, K = 2, 3, and 4 were close. Magnitude of ΔK was small. LnP(K) was highest at K = 1 with the trajectory of points declining and confidence intervals increasing. K = 1 selected. |
| 1.2 | 10 | | 1 | San Juan and Mt. Taylor region | ΔK was highest at K = 8 with a smaller peak at K = 5. Magnitude of ΔK was small. LnP(K) was highest at K = 1 with K = 5 well below and and K = 8 receiving similar support as K = 9 and K = 10. K = 1 selected. |
| 1.3 | 7 | | 1 | San Juan region | ΔK highest at K = 5, however, K = 2 and K = 4 received similar levels of support. Magnitude of ΔK was small. LnP(K) highest at K = 1 with large CIs for the other K values. K = 1 selected. |
| 2 | 5 | | 2 | Trans-Pecos region | ΔK and LnP(K) highest at K = 2. Q-values were not split 50/50. K = 2 selected |
| 2.1 | 3 | | 1 | No pattern | K = 1 selected |
| 2.2 | 5 | | 1 | No pattern | K = 1 selected |
| 3 | 11 | | 2 | Sky Islands South I-10 | ΔK and LnP(K) highest at K = 2. A second, but smaller, peak at K = 6 for ΔK but same peak did not occur for Ln(K). K = 2 selected |

Table S1 continued. Justification for splitting structure identified clusters when investigating hierarchical structure for American black bears (*Ursus americanus*) in the American Southwest and northern Mexico. We split each identified cluster until K = 1.

| Cluster | K tested | K selected | Cluster location | Justification for K |
| --- | --- | --- | --- | --- |
| 3.1 | 4 | 1 | Huachuca and Santa Rita Mountains | ΔK highest at K = 2 and LnP(K) highest at K = 1. K = 1 selected because Evanno cannot evaluate K = 1 |
| 3.2 | 11 | 1 | Chiricahua complex | ΔK highest at K = 3. LnP(K) highest at K = 2 with K = 1 a close second. The Q-values are all split in evenly into thirds suggesting false structure. K = 1 selected. |
| 4 | 3 | 1 | Boulder Mountain | LnP(K) highest at K = 1 with larges CIs for K = 2. ΔK could not be evaluated outside of K = 2. K = 1 selected. |
| 5 | 14 | 2 | Sangre de Cristo Mountains | ΔK highest at K = 2. Magnitude of ΔK was larger than other K = 2 scenarios. LnP(K) was slightly higher for K = 1 than K = 2, which also had small CIs. Q-values were not split 50/50. K = 2 selected. |
| 5.1 | 11 | 1 | Sangre de Cristo Mountains | ΔK highest at K = 3. Magnitude of ΔK was small. LnP(K) highest at K = 1 with small CIs. K = 1 selected. |
| 5.2 | 9 | 1 | Sangre de Cristo Mountains | ΔK highest at K = 5. Magnitude of ΔK was extremely small. LnP(K) highest at K = 1. K = 1 selected. |
| 6 | 15 | 1 | Central Arizona, Gila, Zuni Region | ΔK highest at K = 2. LnP(K) highest at K = 1. Q-values were split 50/50. K = 1 selected |
| 7 | 13 | 2 | Sacramento and Sandia mountains | ΔK and LnP(K) highest at K = 2. K = 2 selected. |
| 7.1 | 13 | 1 | Sacramento Mountains | ΔK highest at K = 10. Magnitude of ΔK extremely small. LnP(K) highest at K = 1 with all other values showing extremely large CIs. K = 1 selected. |
| 7.2 | 10 | 1 | Sandia and Manzano mountains | ΔK highest at K = 8 with another peak at K = 3. Magnitude of ΔK extremely small. LnP(K) highest at K = 1. K = 1 selected. |

Table S2. Frequency of null alleles and its 95% confidence interval in each structure identified cluster for American black bears (*Ursus americanus*) in the American Southwest and northern Mexico. Bolded values indicate the 95% confidence interval for the allele does not overlap 0.

| Locus | 1.1 | 1.2 | 1.3 | 2.1 | 2.2 | 3.1 | 3.2 |
| --- | --- | --- | --- | --- | --- | --- | --- |
| CXX20 | **-0.11**  **(-0.16– -0.05)** | 0.11  (-0.02–0.27) | 0.06  (-0.05–0.18) | **-0.17**  **(-0.23– -0.14)** | -0.05  (-0.16–0.05) | -0.01  (-0.13–0.13) | 0.04  (-0.09–0.20) |
| G1A | 0.10  (-0.08–0.34) | **-0.06**  **(-0.09– -0.04)** | -0.01  (-0.12–0.09) | **-0.09**  **(-0.20– -0.03)** | 0.01  (-0.16–0.16) | **-0.06**  **(-0.10– -0.04)** | 0.04  (-0.08–0.19) |
| G1D | 0.12  (0.00–0.27) | -0.04  (-0.11–0.02) | 0.05  (-0.05–0.16) | -0.01  (-0.20–0.33) | **-0.15**  **(-0.26– -0.05)** | 0.01  (-0.09–0.13) | **-0.07**  **(-0.13– -0.01)** |
| G10B | -0.01  (-0.11–0.11) | -0.06  (-0.14–0.03) | -0.02  (-0.12–0.09) | -0.05  (-0.21–0.11) | -0.05  (-0.17–0.08) | 0.01  (-0.11–0.13) | 0.06  (-0.08–0.24) |
| G10C | **-0.06**  **(-0.09– -0.03)** | 0.02  (-0.08–0.14) | -0.01  (-0.11–0.13) | -0.03  (-0.21–0.18) | -0.03  (-0.16–0.15) | **-0.03**  **(-0.06– -0.01)** | **-0.02**  **(-0.04– -0.01)** |
| G10H | 0.02  (-0.07–0.13) | 0.02  (-0.07–0.13) | 0.06  (-0.05–0.19) | **-0.11**  **(-0.21– -0.04)** | 0.15  (-0.11–0.68) | **-0.04**  **(-0.08– -0.02)** | 0.08  (-0.05–0.24) |
| G10J | 0.13  (0.00–0.27) | **-0.06**  **(-0.11– -0.01)** | -0.04  (-0.13–0.05) | **-0.20**  **(-0.26– -0.2)** | -0.04  (-0.16–0.05) | **-0.03**  **(-0.07– -0.01)** | 0.07  (-0.06–0.22) |
| G10L | -0.01  (-0.09–0.07) | 0.00  (-0.07–0.08) | 0.02  (-0.07–0.12) | **-0.13**  **(-0.24– -0.06)** | -0.01  (-0.19–0.20) | 0.02  (-0.08–0.13) | 0.08  (-0.04–0.22) |
| G10M | -0.04  (-0.12–0.05) | **-0.14**  **(-0.20– -0.07)** | 0.01  (-0.10–0.12) | 0.09  (-0.14–0.45) | **-0.12**  **(-0.22– -0.03)** | 0.22  (-0.06–0.64) | -0.01  (-0.12–0.11) |
| G100 | **-0.08**  **(-0.13– -0.05)** | 0.05  (-0.06–0.27) | 0.08  (-0.06–0.28) | **-0.1**  **(-0.20– -0.03)** | 0.01  (-0.12–0.13) | **-0.02**  **(-0.05– -0.01)** | **-0.02**  **(-0.06– -0.01)** |
| G10P | 0.05  (-0.09–0.26) | 0.00  (-0.06–0.09) | 0.01  (-0.10–0.14) | -0.05  (-0.19–0.12) | -0.06  (-0.17–0.08) | -0.06  (-0.14–0.02) | -0.02  (-0.11–0.10) |
| G10U | **-0.09**  **(-0.15– -0.03)** | 0.03  (-0.06–0.12) | -0.05  (-0.12–0.01) | **-0.06**  **(-0.16– -0.03)** | -0.09  (-0.24–0.11) | -0.05  (-0.12–0.02) | 0.05  (-0.08–0.23) |
| G10X | 0.01  (-0.08–0.12) | 0.00  (-0.09–0.11) | -0.03  (-0.13–0.08) | **-0.13**  **(-0.21– -0.07)** | 0.04  (-0.13–0.22) | 0.08  (-0.06–0.28) | 0.02  (-0.10–0.17) |
| MU50 | **-0.08**  **(-0.12– -0.04)** | **-0.04**  **(-0.06– -0.01)** | NA | -0.05  (-0.19–0.14) | -0.04  (-0.12–0.04) | NA | **-0.01**  **(-0.03– -0.01)** |
| MU59 | **-0.05**  **(-0.08– -0.02)** | -0.04  (-0.12–0.06) | -0.03  (-0.12–0.06) | NA | **-0.15**  **(-0.26– -0.02)** | 0.00  (-0.11–0.14) | 0.03  (-0.08–0.20) |

Table S2 continued.

| Locus | 4 | 5.1 | 5.2 | 6 | 7.1 | 7.2 |
| --- | --- | --- | --- | --- | --- | --- |
| CXX20 | **-0.06**  **(-0.12– -0.03)** | 0.00  (-0.08–0.11) | 0.07  (-0.03–0.19) | -0.01  (-0.05–0.02) | 0.03  (-0.08–0.19) | 0.03  (-0.07–0.13) |
| G1A | -0.05  (-0.17–0.11) | -0.05  (-0.10–0.02) | -0.04  (-0.09–0.03) | 0.03  (-0.04–0.13) | 0.02  (-0.10–0.15) | -0.08  (-0.17–0.04) |
| G1D | 0.03  (-0.11–0.21) | 0.03  (-0.04–0.10) | -0.02  (-0.09–0.06) | -0.01  (-0.05–0.03) | -0.02  (-0.11–0.07) | 0.04  (-0.08–0.18) |
| G10B | 0.11  (-0.03–0.27) | **-0.07**  **(-0.12– -0.02)** | -0.03  (-0.10–0.07) | 0.03  (-0.03–0.10) | **-0.09**  **(-0.16– -0.01)** | 0.02  (-0.08–0.13) |
| G10C | 0.27  (0.00–0.56) | 0.07  (-0.06–0.25) | 0.03  (-0.05–0.12) | -0.01  (-0.06–0.05) | **-0.06**  **(-0.09– -0.04)** | 0.01  (-0.12–0.21) |
| G10H | -0.03  (-0.12–0.05) | -0.02  (-0.08–0.03) | -0.03  (-0.10–0.04) | 0.01  (-0.05–0.07) | -0.06  (-0.13–0.03) | 0.10  (-0.02–0.26) |
| G10J | 0.09  (-0.07–0.28) | 0.01  (-0.06–0.08) | 0.04  (-0.04–0.13) | 0.00  (-0.05–0.05) | -0.01  (-0.09–0.08) | -0.01  (-0.08–0.07) |
| G10L | -0.04  (-0.11–0.05) | -0.01  (-0.06–0.04) | 0.02  (-0.05–0.09) | 0.01  (-0.03–0.05) | -0.01  (-0.09–0.07) | **-0.07**  **(-0.12– -0.01)** |
| G10M | -0.07  (-0.17–0.02) | 0.04  (-0.05–0.14) | 0.01  (-0.07–0.11) | 0.05  (0.00–0.11) | 0.03  (-0.07–0.12) | -0.03  (-0.11–0.06) |
| G100 | -0.06  (-0.20–0.14) | -0.01  (-0.09–0.09) | 0.06  (-0.06–0.22) | 0.06  (-0.04–0.22) | 0.23  (-0.03–1.00) | **-0.04**  **(-0.07– -0.02)** |
| G10P | -0.02  (-0.14–0.11) | 0.04  (-0.03–0.12) | 0.03  (-0.06–0.18) | 0.01  (-0.04–0.07) | **-0.12**  **(-0.19– -0.04)** | -0.07  (-0.16–0.06) |
| G10U | 0.01  (-0.12–0.19) | -0.01  (-0.09–0.09) | -0.05  (-0.12–0.03) | 0.01  (-0.04–0.06) | **-0.06**  **(-0.11– -0.01)** | -0.02  (-0.10–0.08) |
| G10X | 0.10  (-0.04–0.28) | -0.01  (-0.08–0.06) | -0.04  (-0.12–0.05) | 0.01  (-0.04–0.06) | -0.01  (-0.11–0.10) | -0.04  (-0.15–0.09) |
| MU50 | **-0.01**  **(-0.05– -0.01)** | **-0.03**  **(-0.06– -0.02)** | 0.00  (-0.01–0.00) | -0.01  (-0.05–0.06) | NA | 0.09  (-0.06–0.45) |
| MU59 | -0.06  (-0.13–0.00) | -0.01  (-0.07–0.06) | -0.03  (-0.09–0.05) | 0.05  (-0.01–0.11) | -0.05  (-0.12–0.03) | -0.04  (-0.10–0.03) |

Table S6. Estimated linkage disequilibrium for 15 loci used to characterize the genetic structure for American black bears (*Ursus americanus*) in the American Southwest and northern Mexico. Bolded values indicate a statistically significant test after applying a Bonferroni correction of α < 0.0005. structure clusters 1.1 (below diagonal) and 1.2 (above diagonal).

| Locus | CXX20 | G10B | G10C | G10H | G10J | G10L | G10M | G10O | G10P | G10U | G10X | G1A | G1D | MU50 | MU59 |
| --- | --- | --- | --- | --- | --- | --- | --- | --- | --- | --- | --- | --- | --- | --- | --- |
| CXX20 | -- | 0.81 | 0.23 | 0.28 | 0.26 | 0.44 | 0.53 | 0.22 | 0.99 | 0.83 | 0.09 | 0.16 | 0.04 | 0.28 | 0.30 |
| G10B | 0.33 | -- | 0.29 | 0.36 | 0.04 | 0.49 | 0.71 | 0.42 | 0.33 | 0.59 | 0.78 | 0.15 | 0.67 | 0.20 | 0.47 |
| G10C | 0.12 | 0.82 | -- | 0.10 | 0.51 | 0.21 | 0.55 | 0.30 | 0.32 | 0.66 | 0.18 | 0.40 | 0.89 | 0.19 | 0.96 |
| G10H | 0.17 | 0.02 | 0.75 | -- | 0.72 | 0.45 | 0.98 | 0.12 | 0.16 | 0.77 | 0.42 | 0.63 | 0.33 | 0.10 | 0.50 |
| G10J | 0.51 | 0.89 | 0.75 | 0.72 | -- | 0.97 | 0.29 | 0.86 | 1.00 | 0.48 | 0.92 | 0.23 | 0.22 | 0.70 | 0.68 |
| G10L | 0.01 | 0.91 | 0.95 | 0.09 | 0.56 | -- | 0.76 | 0.47 | 0.10 | 0.40 | 0.89 | 0.21 | 0.75 | 0.12 | 0.85 |
| G10M | 0.10 | 0.25 | 0.15 | 0.63 | 0.20 | 0.77 | -- | 0.96 | 0.86 | 0.07 | 0.78 | 0.15 | 0.16 | 0.88 | 0.83 |
| G10O | 0.18 | 0.07 | 1.00 | 0.84 | 0.65 | 0.81 | 0.04 | -- | 0.86 | 0.23 | 0.29 | 0.96 | 1.00 | 0.90 | 0.17 |
| G10P | 0.67 | 0.81 | 0.39 | 0.78 | 0.48 | 0.88 | 0.13 | 0.94 | -- | 0.44 | 0.69 | 0.89 | 0.22 | 0.27 | 0.49 |
| G10U | 0.28 | 0.98 | 0.13 | 0.09 | 0.24 | 0.27 | 0.55 | 0.88 | 0.64 | -- | 0.62 | 0.60 | 0.29 | 0.09 | 0.27 |
| G10X | 0.39 | 0.33 | 0.06 | 0.45 | 0.09 | 0.86 | 0.53 | 0.50 | 0.66 | 0.14 | -- | 0.53 | 0.98 | 0.71 | 0.01 |
| G1A | 0.68 | 0.85 | 0.06 | 0.87 | 0.41 | 0.55 | 0.38 | 0.87 | 0.34 | 0.99 | 0.53 | -- | 0.50 | 0.83 | 0.24 |
| G1D | 0.01 | 0.60 | 0.15 | 0.32 | 0.22 | 0.29 | 0.69 | 0.04 | 0.43 | 0.09 | 0.25 | 0.21 | -- | 0.37 | 0.97 |
| MU50 | 0.46 | 0.58 | 1.00 | 0.54 | 0.53 | 0.13 | 0.22 | 0.26 | 0.36 | 0.21 | 0.43 | 0.70 | 0.07 | -- | 0.18 |
| MU59 | 0.34 | 0.25 | 0.39 | 0.35 | 0.13 | 0.96 | 0.00 | 0.22 | 0.76 | 0.00 | 0.20 | 0.85 | 0.54 | 0.53 | -- |

Table S4. Estimated linkage disequilibrium for 15 loci used to characterize the genetic structure for American black bears (*Ursus americanus*) in the American Southwest and northern Mexico. Bolded values indicate a statistically significant test after applying a Bonferroni correction of α < 0.0005. structure clusters 1.3 (below diagonal) and 2.1 (above diagonal).

| Locus | CXX20 | G10B | G10C | G10H | G10J | G10L | G10M | G10O | G10P | G10U | G10X | G1A | G1D | MU50 | MU59 |
| --- | --- | --- | --- | --- | --- | --- | --- | --- | --- | --- | --- | --- | --- | --- | --- |
| CXX20 | -- | 0.03 | 1.00 | 0.82 | 0.39 | 0.50 | 0.72 | 0.95 | 1.00 | 1.00 | 1.00 | 1.00 | 1.00 | 1.00 | NA |
| G10B | 0.11 | -- | 0.75 | 1.00 | 0.83 | 1.00 | 0.50 | 0.64 | 0.45 | 1.00 | 1.00 | 0.79 | 0.92 | 0.45 | NA |
| G10C | 0.70 | 0.78 | -- | 0.18 | 1.00 | 0.31 | 1.00 | 0.91 | 1.00 | 0.19 | 1.00 | 0.24 | 0.57 | 0.22 | NA |
| G10H | 0.65 | 0.31 | 0.79 | -- | 1.00 | 0.01 | 0.94 | 0.41 | 1.00 | 0.03 | 1.00 | 0.36 | 0.91 | 1.00 | NA |
| G10J | 0.74 | 0.69 | 0.30 | 0.70 | -- | 1.00 | 0.28 | 0.41 | 1.00 | 1.00 | 0.48 | 1.00 | 0.91 | 1.00 | NA |
| G10L | 0.04 | 0.03 | 0.87 | 1.00 | 0.51 | -- | 1.00 | 0.33 | 1.00 | 0.22 | 1.00 | 0.24 | 0.66 | 1.00 | NA |
| G10M | 0.91 | 0.52 | 0.47 | 0.63 | 0.95 | 0.48 | -- | 0.81 | 0.37 | 0.61 | 0.37 | 0.57 | 0.25 | 0.37 | NA |
| G10O | 0.99 | 0.46 | 0.41 | 0.54 | 0.17 | 0.19 | 0.27 | -- | 0.12 | 0.31 | 0.12 | 0.29 | 0.71 | 1.00 | NA |
| G10P | 0.58 | 0.23 | 0.65 | 0.93 | 0.81 | 0.30 | 0.57 | 0.86 | -- | 0.89 | 0.11 | 0.24 | 0.02 | 1.00 | NA |
| G10U | 0.55 | 0.22 | 0.99 | 0.46 | 0.32 | 0.36 | 0.45 | 0.59 | 0.85 | -- | 0.33 | 1.00 | 0.75 | 0.89 | NA |
| G10X | 0.01 | 0.93 | 0.95 | 0.17 | 0.67 | 0.98 | 0.92 | 0.55 | 0.99 | 0.96 | -- | 0.76 | 0.31 | 1.00 | NA |
| G1A | 0.28 | 0.97 | 0.04 | 0.23 | 0.14 | 0.82 | 0.59 | 0.42 | 0.02 | 0.34 | 0.54 | -- | 0.04 | 0.23 | NA |
| G1D | 0.44 | 0.05 | 0.91 | 0.37 | 0.65 | 1.00 | 0.98 | 0.25 | 0.72 | 0.93 | 0.68 | 0.97 | -- | 1.00 | NA |
| MU50 | NA | NA | NA | NA | NA | NA | NA | NA | NA | NA | NA | NA | NA | -- | NA |
| MU59 | 0.98 | 0.75 | 0.58 | 0.18 | 0.99 | 0.49 | 0.02 | 0.47 | 0.15 | 0.38 | 0.59 | 0.20 | 0.48 | NA | -- |

Table S6. Estimated linkage disequilibrium for 15 loci used to characterize the genetic structure for American black bears (*Ursus americanus*) in the American Southwest and northern Mexico. Bolded values indicate a statistically significant test after applying a Bonferroni correction of α < 0.0005. structure clusters 2.2 (below diagonal) and 3.1 (above diagonal).

| Locus | CXX20 | G10B | G10C | G10H | G10J | G10L | G10M | G10O | G10P | G10U | G10X | G1A | G1D | MU50 | MU59 |
| --- | --- | --- | --- | --- | --- | --- | --- | --- | --- | --- | --- | --- | --- | --- | --- |
| CXX20 | -- | 0.36 | 0.86 | 0.02 | 0.31 | 0.05 | 0.96 | 0.09 | 0.32 | 0.57 | 0.31 | 0.60 | 0.65 | NA | 0.71 |
| G10B | 0.17 | -- | 0.76 | 0.45 | 0.92 | 0.46 | 0.36 | 0.11 | 0.20 | 0.75 | 0.64 | 0.80 | 0.66 | NA | 0.97 |
| G10C | 0.83 | 0.11 | -- | 0.69 | 0.68 | 0.55 | 1.00 | 1.00 | 0.93 | 0.39 | 0.58 | 1.00 | 0.77 | NA | 1.00 |
| G10H | 1.00 | 0.41 | 0.63 | -- | 0.64 | 0.81 | 1.00 | 0.19 | 0.76 | 0.14 | 0.27 | 0.20 | 0.98 | NA | 0.19 |
| G10J | 1.00 | 0.23 | 0.38 | 0.95 | -- | 0.93 | 0.75 | 0.50 | 0.64 | 0.82 | 0.15 | 0.31 | 0.17 | NA | 0.55 |
| G10L | 1.00 | 0.60 | 0.40 | 0.37 | 1.00 | -- | 0.61 | 0.27 | 0.48 | 0.93 | 0.07 | 0.13 | 0.29 | NA | 0.99 |
| G10M | 0.53 | 0.96 | 0.62 | 0.48 | 1.00 | 0.81 | -- | 1.00 | 0.92 | 0.84 | 0.59 | 0.11 | 0.83 | NA | 0.74 |
| G10O | 1.00 | 1.00 | 1.00 | 1.00 | 1.00 | 1.00 | 1.00 | -- | 0.56 | 0.62 | 0.51 | 0.54 | 0.36 | NA | 0.90 |
| G10P | 1.00 | 0.69 | 1.00 | 0.53 | 1.00 | 1.00 | 0.52 | 1.00 | -- | 0.30 | 0.40 | 0.41 | 0.01 | NA | 0.16 |
| G10U | 1.00 | 0.99 | 0.02 | 0.73 | 0.68 | 0.52 | 0.59 | 0.21 | 0.68 | -- | 0.24 | 0.13 | 0.65 | NA | 0.03 |
| G10X | 1.00 | 0.22 | 0.37 | 0.95 | 1.00 | 0.11 | 1.00 | 1.00 | 0.26 | 0.67 | -- | 0.00 | 0.36 | NA | 0.17 |
| G1A | 0.51 | 0.63 | 0.59 | 1.00 | 0.19 | 1.00 | 0.35 | 0.43 | 0.59 | 0.08 | 0.95 | -- | 0.51 | NA | 0.62 |
| G1D | 1.00 | 0.57 | 0.29 | 0.06 | 0.84 | 0.18 | 0.87 | 1.00 | 0.33 | 0.11 | 0.32 | 0.91 | -- | NA | 0.42 |
| MU50 | NA | NA | NA | NA | NA | NA | NA | NA | NA | NA | NA | NA | NA | -- | NA |
| MU59 | 1.00 | 0.25 | 0.18 | 0.91 | 0.38 | 0.40 | 0.47 | 1.00 | 0.39 | 0.30 | 0.08 | 0.20 | 0.72 | NA | -- |

Table S6. Estimated linkage disequilibrium for 15 loci used to characterize the genetic structure for American black bears (*Ursus americanus*) in the American Southwest and northern Mexico. Bolded values indicate a statistically significant test after applying a Bonferroni correction of α < 0.0005. structure clusters 3.2 (below diagonal) and 4 (above diagonal).

| Locus | CXX20 | G10B | G10C | G10H | G10J | G10L | G10M | G10O | G10P | G10U | G10X | G1A | G1D | MU50 | MU59 |
| --- | --- | --- | --- | --- | --- | --- | --- | --- | --- | --- | --- | --- | --- | --- | --- |
| CXX20 | -- | **< 0.01** | 0.65 | **< 0.01** | 0.01 | 0.36 | 0.09 | 0.08 | 0.55 | 0.97 | 0.71 | 0.55 | 0.69 | 0.14 | 0.03 |
| G10B | 0.11 | -- | 0.44 | 0.49 | 0.78 | 0.91 | 0.50 | 0.97 | 0.36 | 0.19 | 0.89 | 0.52 | 0.80 | 0.57 | 0.23 |
| G10C | 1.00 | 0.19 | -- | 0.96 | 0.75 | 0.29 | 0.80 | 0.12 | 0.06 | 0.98 | 0.35 | 0.60 | 0.39 | 0.62 | 0.60 |
| G10H | 0.96 | 0.94 | 0.08 | -- | 0.02 | 0.18 | 0.07 | 0.04 | 0.20 | 0.11 | 0.97 | 0.44 | 0.46 | 0.19 | 0.13 |
| G10J | 0.77 | 0.47 | 1.00 | 0.71 | -- | 0.28 | 0.09 | 0.53 | 0.14 | 0.99 | 0.89 | 0.25 | 0.79 | 1.00 | **< 0.01** |
| G10L | 0.82 | 0.94 | 1.00 | 0.23 | 0.14 | -- | 0.41 | 0.38 | 0.50 | 0.32 | 0.23 | 0.71 | 0.01 | 0.48 | 0.75 |
| G10M | 0.97 | 0.25 | 0.22 | 0.61 | 0.67 | 0.28 | -- | 0.22 | 0.28 | 0.63 | 0.95 | 0.13 | 0.03 | 0.61 | 0.48 |
| G10O | 0.19 | 0.88 | 1.00 | 0.58 | 0.47 | 0.13 | 0.53 | -- | 0.23 | 0.74 | 0.66 | 0.13 | 0.04 | 0.47 | 0.73 |
| G10P | 0.09 | 0.65 | 1.00 | 0.46 | 0.51 | 0.19 | 0.97 | 0.88 | -- | 0.72 | 0.44 | 0.52 | 0.77 | 1.00 | 0.72 |
| G10U | 0.48 | **< 0.01** | 0.24 | 0.47 | 0.96 | 0.49 | 0.15 | 0.24 | 0.89 | -- | 0.96 | 1.00 | 0.73 | 1.00 | 0.21 |
| G10X | 0.98 | 0.43 | 0.07 | 0.17 | 0.09 | **< 0.01** | 0.57 | 0.07 | 0.10 | 0.64 | -- | 0.17 | 1.00 | 1.00 | 0.33 |
| G1A | 0.14 | 0.50 | 0.47 | 0.84 | 0.80 | 0.52 | 0.89 | 0.91 | 0.11 | 0.88 | 0.60 | -- | 0.29 | 1.00 | 0.92 |
| G1D | 0.16 | 0.72 | 1.00 | 0.53 | 0.48 | 0.44 | 0.34 | 0.96 | 0.42 | 0.44 | 0.44 | 0.06 | -- | 0.91 | 0.57 |
| MU50 | 1.00 | 0.15 | 1.00 | 0.36 | 1.00 | 1.00 | 1.00 | 1.00 | 1.00 | 0.09 | 1.00 | 0.67 | 0.76 | -- | 1.00 |
| MU59 | 0.98 | 0.69 | 0.64 | 0.02 | 0.46 | 0.57 | 0.87 | 0.23 | 0.36 | 0.62 | 0.13 | 0.73 | 0.99 | 0.27 | -- |

Table S7. Estimated linkage disequilibrium for 15 loci used to characterize the genetic structure for American black bears (*Ursus americanus*) in the American Southwest and northern Mexico. Bolded values indicate a statistically significant test after applying a Bonferroni correction of α < 0.0005. structure clusters 5.1 (below diagonal) and 5.2 (above diagonal).

| Locus | CXX20 | G10B | G10C | G10H | G10J | G10L | G10M | G10O | G10P | G10U | G10X | G1A | G1D | MU50 | MU59 |
| --- | --- | --- | --- | --- | --- | --- | --- | --- | --- | --- | --- | --- | --- | --- | --- |
| CXX20 | -- | 0.56 | 0.90 | 0.04 | 0.99 | 0.89 | 0.98 | 0.56 | 0.46 | 0.80 | 0.14 | 0.26 | 0.16 | 1.00 | 0.81 |
| G10B | 0.03 | -- | 0.82 | 0.92 | 0.44 | 0.73 | 0.50 | 0.23 | 0.87 | 0.56 | 0.77 | 0.52 | 0.11 | 1.00 | 0.93 |
| G10C | 0.35 | 0.84 | -- | 0.49 | 0.63 | 0.23 | 0.96 | 0.93 | 0.53 | 0.97 | 0.12 | 0.10 | 0.30 | 1.00 | 0.67 |
| G10H | 0.18 | 0.61 | 0.43 | -- | 0.36 | 0.89 | 0.89 | 0.52 | 0.06 | 0.92 | 0.69 | 0.79 | 0.87 | 0.57 | 0.98 |
| G10J | 0.66 | 0.43 | 0.62 | 0.55 | -- | 0.76 | 0.19 | 0.76 | 0.68 | 0.91 | 0.91 | 0.29 | 0.48 | 0.73 | 0.26 |
| G10L | 0.39 | 0.60 | 0.90 | 0.97 | 0.95 | -- | 0.58 | 0.34 | 0.70 | 0.72 | 0.06 | 0.55 | 0.85 | 1.00 | 0.25 |
| G10M | 0.03 | 0.32 | 0.34 | 0.49 | 0.66 | 0.03 | -- | 0.35 | 0.32 | 0.03 | 0.42 | 0.02 | 0.64 | 0.59 | 0.69 |
| G10O | 0.15 | 0.77 | 0.15 | 0.42 | 0.21 | 0.19 | 0.21 | -- | 0.98 | 0.51 | 0.70 | 0.43 | 0.27 | 0.33 | 0.41 |
| G10P | 0.96 | 0.91 | 0.70 | 0.91 | 0.72 | 0.39 | 0.88 | 0.07 | -- | 0.23 | 0.91 | 0.85 | 0.65 | 1.00 | 0.93 |
| G10U | 0.26 | 0.72 | 0.72 | 0.04 | 0.18 | 0.30 | 0.56 | 0.45 | 0.89 | -- | 0.83 | 0.34 | 0.32 | 0.15 | 0.46 |
| G10X | 0.92 | 0.52 | 0.40 | 0.48 | 0.02 | 0.92 | 0.51 | 0.21 | 0.60 | 0.59 | -- | 0.86 | 0.62 | 0.34 | 0.15 |
| G1A | 0.53 | 0.58 | 0.98 | 0.77 | 0.03 | 0.43 | 0.53 | 0.95 | 0.79 | 0.99 | 0.98 | -- | 0.93 | 0.11 | 0.61 |
| G1D | 0.80 | 0.84 | 0.74 | 0.67 | 0.94 | 0.94 | 0.78 | 0.43 | 0.05 | 0.98 | 0.58 | 0.92 | -- | 0.73 | 0.69 |
| MU50 | 0.05 | 0.16 | 0.16 | 0.71 | 0.30 | 0.08 | 0.26 | 0.47 | 0.47 | 0.49 | 0.51 | 0.86 | 0.87 | -- | 0.70 |
| MU59 | 0.78 | 0.60 | 0.13 | 0.20 | 0.61 | 0.13 | 0.92 | 0.36 | 0.85 | 0.77 | 0.98 | 0.41 | 0.95 | 0.73 | -- |

Table S8. Estimated linkage disequilibrium for 15 loci used to characterize the genetic structure for American black bears (*Ursus americanus*) in the American Southwest and northern Mexico. Bolded values indicate a statistically significant test after applying a Bonferroni correction of α < 0.0005. structure clusters 6 (below diagonal) and 7.1 (above diagonal).

| Locus | CXX20 | G10B | G10C | G10H | G10J | G10L | G10M | G10O | G10P | G10U | G10X | G1A | G1D | MU50 | MU59 |
| --- | --- | --- | --- | --- | --- | --- | --- | --- | --- | --- | --- | --- | --- | --- | --- |
| CXX20 | -- | 0.05 | 0.90 | 0.47 | 0.29 | 0.62 | 0.52 | 0.11 | 0.86 | 0.31 | 0.05 | 0.27 | 0.91 | NA | 0.87 |
| G10B | 0.60 | -- | 0.85 | 0.43 | 0.83 | 0.16 | 0.75 | 0.84 | 0.80 | 0.77 | 0.08 | 0.35 | 0.13 | NA | 0.95 |
| G10C | 0.05 | 0.28 | -- | 0.58 | 0.39 | 0.41 | 0.42 | 1.00 | 0.54 | 0.31 | 0.73 | 0.83 | 0.68 | NA | 0.13 |
| G10H | 0.47 | 0.20 | 0.02 | -- | 0.76 | 0.25 | 0.81 | 0.61 | 0.53 | 0.89 | 0.93 | 0.81 | 0.11 | NA | 0.65 |
| G10J | 0.24 | 0.12 | 0.02 | 0.94 | -- | 0.19 | 0.77 | 0.85 | 0.48 | 0.00 | 0.46 | 0.78 | 0.80 | NA | 0.83 |
| G10L | 0.82 | 0.26 | 0.44 | 0.41 | 0.99 | -- | 0.75 | 0.35 | 0.24 | 0.99 | 0.38 | 0.13 | 0.83 | NA | 0.79 |
| G10M | 0.01 | 0.19 | 0.02 | 0.42 | 0.47 | 0.18 | -- | 0.01 | 0.27 | 0.40 | 0.21 | 0.58 | 0.09 | NA | 0.67 |
| G10O | 0.02 | 0.98 | 0.03 | 0.94 | 0.01 | 0.34 | 0.76 | -- | 0.73 | 0.17 | 0.64 | 0.37 | 0.21 | NA | 0.83 |
| G10P | 0.69 | 0.74 | 0.42 | 0.91 | 0.40 | 0.13 | 0.76 | 0.81 | -- | 0.80 | 0.40 | 0.81 | 0.36 | NA | 0.16 |
| G10U | 0.85 | 0.36 | 0.78 | 0.41 | 0.12 | 0.70 | 0.12 | 0.39 | 0.04 | -- | 0.94 | 0.53 | 0.91 | NA | 0.50 |
| G10X | 0.39 | 0.72 | 0.84 | 0.21 | 0.73 | 0.11 | 0.19 | 0.47 | 0.09 | 0.10 | -- | 0.00 | 0.98 | NA | 0.30 |
| G1A | 0.09 | 0.46 | 0.95 | 0.56 | 0.46 | 0.43 | 0.35 | 0.61 | 0.10 | 0.00 | 0.83 | -- | 0.92 | NA | 0.75 |
| G1D | 0.13 | 0.57 | 0.87 | 0.98 | 0.98 | 0.36 | 0.94 | 0.45 | 0.83 | 0.69 | 0.73 | 0.70 | -- | NA | 0.84 |
| MU50 | 0.17 | 0.02 | 0.73 | 0.52 | 0.07 | 0.54 | 0.14 | 0.01 | 0.23 | 0.20 | 0.66 | 0.00 | 0.41 | -- | NA |
| MU59 | 0.91 | 0.84 | 0.45 | 0.33 | 0.47 | 0.30 | 0.26 | 0.19 | 0.26 | 0.15 | 0.45 | 0.70 | 0.33 | 0.57 | -- |

Table S9. Estimated linkage disequilibrium for 15 loci used to characterize the genetic structure for American black bears (*Ursus americanus*) in the American Southwest and northern Mexico. Bolded values indicate a statistically significant test after applying a Bonferroni correction of α < 0.0005. structure clusters 7.2 (below diagonal)

| Locus | CXX20 | G10B | G10C | G10H | G10J | G10L | G10M | G10O | G10P | G10U | G10X | G1A | G1D | MU50 | MU59 | |
| --- | --- | --- | --- | --- | --- | --- | --- | --- | --- | --- | --- | --- | --- | --- | --- | --- |
| CXX20 | -- |  |  |  |  |  |  |  |  |  |  |  |  |  |  |  |
| G10B | 0.27 | -- |  |  |  |  |  |  |  |  |  |  |  |  |  |  |
| G10C | 0.52 | 0.30 | -- |  |  |  |  |  |  |  |  |  |  |  |  |  |
| G10H | 0.11 | 0.45 | 0.26 | -- |  |  |  |  |  |  |  |  |  |  |  |  |
| G10J | 0.95 | 0.68 | 0.78 | 0.40 | -- |  |  |  |  |  |  |  |  |  |  |  |
| G10L | 0.42 | 0.77 | 0.86 | 0.20 | 0.35 | -- |  |  |  |  |  |  |  |  |  |  |
| G10M | 0.91 | 0.75 | 0.65 | 0.78 | 0.94 | 0.67 | -- |  |  |  |  |  |  |  |  |  |
| G10O | 0.03 | 0.05 | 1.00 | 0.25 | 0.76 | 0.28 | 0.17 | -- |  |  |  |  |  |  |  |  |
| G10P | 0.62 | 0.68 | 0.67 | 0.49 | 0.22 | 0.35 | 0.22 | 0.04 | -- |  |  |  |  |  |  |  |
| G10U | 0.37 | 0.11 | 0.69 | 0.66 | 0.99 | 0.45 | 0.55 | 0.99 | 0.87 | -- |  |  |  |  |  |  |
| G10X | 0.24 | 0.75 | 0.26 | 0.56 | 0.90 | 0.89 | 0.21 | 0.02 | 0.06 | 0.99 | -- |  |  |  |  |  |
| G1A | 0.37 | 0.37 | 0.42 | 0.99 | 0.55 | 0.90 | 0.16 | 0.83 | 0.54 | 0.39 | 0.84 | -- |  |  |  |  |
| G1D | 0.27 | 0.28 | 0.53 | 0.32 | 0.30 | 0.98 | 0.65 | 0.41 | 0.10 | 0.89 | 0.90 | 0.82 | -- |  |  |  |
| MU50 | 0.01 | 0.49 | 0.80 | 0.30 | 0.24 | 0.95 | 0.19 | 0.24 | 0.71 | 0.72 | 0.11 | 0.06 | 0.63 | -- |  |  |
| MU59 | 0.21 | 0.65 | 0.82 | 0.96 | 0.55 | 0.17 | 0.43 | 0.05 | 0.41 | 0.71 | 0.10 | 0.25 | 0.50 | 0.75 | -- |  |

Table S10. Hardy-Weinberg proportion tests for 15 loci used to characterize the genetic structure for structure identified clusters of American black bears (*Ursus americanus*) in the American Southwest and northern Mexico. Bolded values indicate a statistically significant test after applying a Bonferroni correction of α < 0.003.

| Locus | 1.1 | 1.2 | 1.3 | 2.1 | 2.2 | 3.1 | 3.2 | 4 | 5.1 | 5.2 | 6 | 7.1 | 7.2 |
| --- | --- | --- | --- | --- | --- | --- | --- | --- | --- | --- | --- | --- | --- |
| CXX20 | 0.52 | 0.03 | 0.12 | 0.72 | 0.91 | 1.00 | 0.22 | 1.00 | 0.54 | 0.05 | 0.84 | 0.49 | 0.57 |
| G1A | 0.06 | 1.00 | 0.06 | 1.00 | 0.17 | 1.00 | 0.18 | 0.83 | 0.92 | 0.43 | 0.41 | 0.40 | 0.74 |
| G1D | 0.06 | 0.17 | 0.37 | 1.00 | 0.15 | 0.74 | 0.89 | 0.29 | 0.12 | 0.16 | 0.84 | 0.12 | 0.59 |
| G10B | 0.34 | 0.66 | 0.82 | 0.48 | 0.76 | 0.41 | 0.85 | 0.03 | 0.91 | 0.72 | 0.17 | 0.31 | 0.78 |
| G10C | 1.00 | 0.44 | 0.89 | 1.00 | 0.61 | 1.00 | 1.00 | 0.07 | 0.32 | 0.15 | 0.60 | 1.00 | 1.00 |
| G10H | 0.41 | 0.40 | 0.17 | 1.00 | 0.40 | 1.00 | 0.60 | 0.18 | 0.96 | 0.39 | 0.37 | 0.33 | 0.20 |
| G10J | 0.01 | 0.84 | 0.11 | 0.37 | 0.56 | 1.00 | 0.17 | 0.30 | 0.79 | 0.01 | 0.45 | 0.72 | 0.46 |
| G10L | 0.61 | 0.99 | 0.69 | 1.00 | 0.07 | 0.46 | 0.25 | 0.73 | 0.34 | 0.47 | 0.40 | 0.48 | 0.94 |
| G10M | 0.77 | 0.02 | 0.28 | 0.50 | 0.91 | 0.10 | 0.94 | 0.15 | 0.36 | 0.31 | 0.34 | 0.39 | 0.58 |
| G100 | 1.00 | 0.35 | 0.11 | 1.00 | 0.64 | 1.00 | 1.00 | 1.00 | 0.53 | 0.29 | 0.23 | 0.03 | 1.00 |
| G10P | 0.32 | 0.49 | 0.86 | 1.00 | 0.90 | 0.96 | 0.60 | 0.50 | 0.21 | 0.64 | 0.62 | 0.27 | 0.79 |
| G10U | 0.30 | 0.22 | 0.83 | 1.00 | 0.79 | 0.64 | 0.39 | 0.84 | 0.83 | 0.53 | 0.78 | 0.61 | 0.84 |
| G10X | 0.83 | 0.80 | 0.89 | 1.00 | 0.07 | 0.52 | 0.38 | 0.31 | 0.74 | 0.04 | 0.38 | 0.47 | 0.16 |
| MU50 | 1.00 | 1.00 | 1.00 | 1.00 | 0.97 | 1.00 | 1.00 | 1.00 | 1.00 | 1.00 | 0.78 | 1.00 | 0.37 |
| MU59 | 1.00 | 0.24 | 0.34 | 1.00 | 0.56 | 0.66 | 0.38 | 0.30 | 0.86 | 0.76 | 0.06 | 0.12 | 0.68 |

Table S11. Number of individuals (N), private alleles (A_P_), private alleles using rarefaction (A_PR_), allelic richness using rarefaction (A_R_), observed (H_O_) and expected (H_E_) heterozygosity, and an inbreeding coefficient (F_IS_) and its 95% confidence interval (LCI and UCI) based on 1,000 bootstrap iterations for structure identified clusters of American black bears (*Ursus americanus*) in the American Southwest and northern Mexico.

| Genetic cluster | State | N | A_P_ | A_PR_ | A_R_ | H_O_ | H_E_ | F_IS_ | LCI | UCI |
| --- | --- | --- | --- | --- | --- | --- | --- | --- | --- | --- |
| 1.1 | UT | 35 | 0 | 0.06 | 3.77 | 0.54 | 0.54 | -0.02 | -0.08 | 0.02 |
| 1.2 | CO, NM | 42 | 1 | 0.04 | 3.76 | 0.56 | 0.54 | -0.03 | -0.09 | -0.01 |
| 1.3 | CO, NM | 33 | 2 | 0.10 | 4.30 | 0.57 | 0.58 | 0.01 | -0.06 | 0.05 |
| 2.1 | TX | 9 | 5 | 0.38 | 3.60 | 0.59 | 0.49 | -0.18 | -0.35 | -0.15 |
| 2.2 | TX | 11 | 8 | 0.63 | 4.38 | 0.69 | 0.62 | -0.09 | -0.27 | -0.04 |
| 3.1 | AZ | 33 | 0 | 0.02 | 2.88 | 0.39 | 0.39 | -0.01 | -0.07 | 0.03 |
| 3.2 | AZ | 33 | 0 | 0.04 | 3.48 | 0.44 | 0.47 | 0.04 | -0.03 | 0.08 |
| 4 | UT | 21 | 3 | 0.27 | 3.80 | 0.57 | 0.58 | 0.01 | -0.09 | 0.07 |
| 5.1 | CO, NM | 56 | 1 | 0.06 | 4.54 | 0.64 | 0.64 | -0.01 | -0.05 | 0.02 |
| 5.2 | CO, NM | 54 | 0 | 0.02 | 3.79 | 0.54 | 0.53 | 0.00 | -0.05 | 0.04 |
| 6 | AZ, NM | 145 | 1 | 0.03 | 3.83 | 0.49 | 0.51 | 0.03 | 0.00 | 0.05 |
| 7.1 | NM | 43 | 1 | 0.02 | 3.68 | 0.57 | 0.54 | -0.04 | -0.12 | 0.02 |
| 7.2 | NM | 35 | 1 | 0.04 | 3.69 | 0.56 | 0.54 | -0.02 | -0.09 | 0.03 |

Table S12. Allele frequencies for the 15 microsatellite loci used to characterize the genetic structure for structure identified clusters of American black bears (*Ursus americanus*) in the American Southwest and northern Mexico.

| Locus/allele | 1.1 | 1.2 | 1.3 | 2.1 | 2.2 | 3.1 | 3.2 | 4 | 5.1 | 5.2 | 6 | 7.1 | 7.2 |
| --- | --- | --- | --- | --- | --- | --- | --- | --- | --- | --- | --- | --- | --- |
| CXX20 |  |  |  |  |  |  |  |  |  |  |  |  |  |
| 123 | 0.00 | 0.00 | 0.00 | 0.22 | 0.09 | 0.00 | 0.00 | 0.00 | 0.00 | 0.00 | 0.04 | 0.00 | 0.00 |
| 129 | 0.00 | 0.00 | 0.00 | 0.17 | 0.05 | 0.00 | 0.00 | 0.00 | 0.02 | 0.01 | 0.00 | 0.00 | 0.01 |
| 131 | 0.41 | 0.55 | 0.24 | 0.00 | 0.00 | 0.44 | 0.65 | 0.02 | 0.55 | 0.61 | 0.81 | 0.74 | 0.36 |
| 133 | 0.21 | 0.21 | 0.26 | 0.00 | 0.00 | 0.00 | 0.03 | 0.10 | 0.23 | 0.16 | 0.04 | 0.12 | 0.30 |
| 137 | 0.13 | 0.08 | 0.11 | 0.00 | 0.05 | 0.00 | 0.00 | 0.00 | 0.09 | 0.17 | 0.02 | 0.00 | 0.00 |
| 139 | 0.23 | 0.13 | 0.26 | 0.11 | 0.55 | 0.11 | 0.21 | 0.83 | 0.05 | 0.03 | 0.05 | 0.07 | 0.17 |
| 141 | 0.00 | 0.00 | 0.00 | 0.44 | 0.14 | 0.00 | 0.00 | 0.00 | 0.01 | 0.00 | 0.00 | 0.00 | 0.00 |
| 143 | 0.01 | 0.02 | 0.12 | 0.06 | 0.14 | 0.46 | 0.08 | 0.00 | 0.05 | 0.03 | 0.03 | 0.07 | 0.16 |
| 145 | 0.00 | 0.00 | 0.00 | 0.00 | 0.00 | 0.00 | 0.00 | 0.00 | 0.00 | 0.00 | 0.01 | 0.00 | 0.00 |
| 147 | 0.00 | 0.00 | 0.02 | 0.00 | 0.00 | 0.00 | 0.03 | 0.05 | 0.00 | 0.00 | 0.00 | 0.00 | 0.00 |
| G1A |  |  |  |  |  |  |  |  |  |  |  |  |  |
| 184 | 0.00 | 0.00 | 0.00 | 0.00 | 0.00 | 0.00 | 0.00 | 0.19 | 0.00 | 0.00 | 0.00 | 0.00 | 0.00 |
| 188 | 0.01 | 0.06 | 0.05 | 0.00 | 0.00 | 0.00 | 0.00 | 0.00 | 0.08 | 0.01 | 0.04 | 0.11 | 0.30 |
| 192 | 0.73 | 0.83 | 0.61 | 0.00 | 0.00 | 0.83 | 0.56 | 0.52 | 0.56 | 0.73 | 0.86 | 0.66 | 0.60 |
| 194 | 0.21 | 0.04 | 0.18 | 0.83 | 0.77 | 0.02 | 0.11 | 0.29 | 0.18 | 0.17 | 0.08 | 0.21 | 0.10 |
| 196 | 0.01 | 0.00 | 0.06 | 0.00 | 0.00 | 0.00 | 0.00 | 0.00 | 0.07 | 0.02 | 0.00 | 0.00 | 0.00 |
| 198 | 0.01 | 0.07 | 0.05 | 0.17 | 0.18 | 0.00 | 0.00 | 0.00 | 0.09 | 0.07 | 0.00 | 0.00 | 0.00 |
| 200 | 0.00 | 0.00 | 0.00 | 0.00 | 0.05 | 0.00 | 0.00 | 0.00 | 0.00 | 0.00 | 0.01 | 0.00 | 0.00 |
| 202 | 0.01 | 0.00 | 0.06 | 0.00 | 0.00 | 0.06 | 0.06 | 0.00 | 0.02 | 0.00 | 0.00 | 0.02 | 0.00 |
| 204 | 0.00 | 0.00 | 0.00 | 0.00 | 0.00 | 0.09 | 0.27 | 0.00 | 0.00 | 0.00 | 0.01 | 0.00 | 0.00 |
| G1D |  |  |  |  |  |  |  |  |  |  |  |  |  |
| 172 | 0.31 | 0.13 | 0.08 | 0.00 | 0.00 | 0.30 | 0.23 | 0.00 | 0.12 | 0.26 | 0.18 | 0.16 | 0.17 |
| 174 | 0.00 | 0.01 | 0.14 | 0.00 | 0.00 | 0.00 | 0.00 | 0.21 | 0.05 | 0.04 | 0.01 | 0.00 | 0.00 |
| 176 | 0.14 | 0.30 | 0.20 | 0.28 | 0.14 | 0.05 | 0.08 | 0.38 | 0.13 | 0.08 | 0.15 | 0.34 | 0.14 |
| 178 | 0.00 | 0.00 | 0.00 | 0.00 | 0.00 | 0.36 | 0.08 | 0.00 | 0.01 | 0.00 | 0.01 | 0.00 | 0.00 |
| 180 | 0.11 | 0.24 | 0.20 | 0.00 | 0.00 | 0.00 | 0.03 | 0.12 | 0.19 | 0.10 | 0.03 | 0.30 | 0.09 |
| 182 | 0.00 | 0.00 | 0.00 | 0.06 | 0.05 | 0.00 | 0.00 | 0.00 | 0.00 | 0.00 | 0.00 | 0.00 | 0.00 |
| 184 | 0.41 | 0.31 | 0.35 | 0.61 | 0.27 | 0.24 | 0.49 | 0.29 | 0.33 | 0.46 | 0.52 | 0.19 | 0.60 |
| 186 | 0.01 | 0.01 | 0.05 | 0.00 | 0.55 | 0.05 | 0.11 | 0.00 | 0.17 | 0.06 | 0.09 | 0.01 | 0.00 |
| 188 | 0.00 | 0.00 | 0.00 | 0.06 | 0.00 | 0.00 | 0.00 | 0.00 | 0.00 | 0.00 | 0.00 | 0.00 | 0.00 |
| G10B |  |  |  |  |  |  |  |  |  |  |  |  |  |
| 154 | 0.00 | 0.00 | 0.00 | 0.06 | 0.00 | 0.00 | 0.00 | 0.00 | 0.00 | 0.00 | 0.00 | 0.00 | 0.00 |
| 156 | 0.00 | 0.00 | 0.02 | 0.67 | 0.68 | 0.00 | 0.02 | 0.00 | 0.03 | 0.00 | 0.02 | 0.04 | 0.43 |
| 158 | 0.17 | 0.01 | 0.05 | 0.00 | 0.00 | 0.00 | 0.03 | 0.14 | 0.09 | 0.04 | 0.03 | 0.00 | 0.01 |
| 160 | 0.00 | 0.00 | 0.03 | 0.06 | 0.18 | 0.03 | 0.00 | 0.38 | 0.05 | 0.03 | 0.00 | 0.00 | 0.00 |
| 162 | 0.20 | 0.21 | 0.27 | 0.22 | 0.09 | 0.42 | 0.35 | 0.07 | 0.41 | 0.48 | 0.15 | 0.43 | 0.20 |
| 164 | 0.59 | 0.60 | 0.61 | 0.00 | 0.05 | 0.44 | 0.58 | 0.33 | 0.27 | 0.35 | 0.62 | 0.33 | 0.21 |
| 166 | 0.04 | 0.17 | 0.03 | 0.00 | 0.00 | 0.11 | 0.03 | 0.07 | 0.15 | 0.09 | 0.18 | 0.21 | 0.14 |
| 168 | 0.00 | 0.00 | 0.00 | 0.00 | 0.00 | 0.00 | 0.00 | 0.00 | 0.00 | 0.01 | 0.00 | 0.00 | 0.00 |
| 170 | 0.00 | 0.01 | 0.00 | 0.00 | 0.00 | 0.00 | 0.00 | 0.00 | 0.00 | 0.00 | 0.00 | 0.00 | 0.00 |
| G10C |  |  |  |  |  |  |  |  |  |  |  |  |  |
| 197 | 0.07 | 0.16 | 0.12 | 0.00 | 0.00 | 0.06 | 0.03 | 0.00 | 0.11 | 0.07 | 0.07 | 0.07 | 0.00 |
| 199 | 0.86 | 0.76 | 0.74 | 0.00 | 0.00 | 0.94 | 0.97 | 0.52 | 0.85 | 0.87 | 0.76 | 0.85 | 0.77 |
| 203 | 0.07 | 0.04 | 0.14 | 0.00 | 0.00 | 0.00 | 0.00 | 0.48 | 0.04 | 0.06 | 0.11 | 0.08 | 0.23 |
| 209 | 0.00 | 0.00 | 0.00 | 0.33 | 0.00 | 0.00 | 0.00 | 0.00 | 0.00 | 0.00 | 0.00 | 0.00 | 0.00 |
| 211 | 0.00 | 0.04 | 0.00 | 0.06 | 0.18 | 0.00 | 0.00 | 0.00 | 0.01 | 0.00 | 0.07 | 0.00 | 0.00 |
| 213 | 0.00 | 0.00 | 0.00 | 0.11 | 0.09 | 0.00 | 0.00 | 0.00 | 0.00 | 0.00 | 0.00 | 0.00 | 0.00 |
| 215 | 0.00 | 0.01 | 0.00 | 0.50 | 0.73 | 0.00 | 0.00 | 0.00 | 0.00 | 0.00 | 0.00 | 0.00 | 0.00 |
| G10H |  |  |  |  |  |  |  |  |  |  |  |  |  |
| 231 | 0.07 | 0.00 | 0.05 | 0.00 | 0.00 | 0.00 | 0.00 | 0.00 | 0.00 | 0.00 | 0.01 | 0.04 | 0.04 |
| 237 | 0.54 | 0.49 | 0.68 | 0.06 | 0.18 | 0.88 | 0.62 | 0.14 | 0.25 | 0.26 | 0.70 | 0.54 | 0.63 |
| 239 | 0.17 | 0.04 | 0.06 | 0.17 | 0.05 | 0.02 | 0.03 | 0.57 | 0.31 | 0.46 | 0.12 | 0.02 | 0.01 |
| 241 | 0.07 | 0.31 | 0.08 | 0.72 | 0.77 | 0.00 | 0.02 | 0.05 | 0.13 | 0.07 | 0.02 | 0.01 | 0.03 |
| 243 | 0.00 | 0.00 | 0.00 | 0.00 | 0.00 | 0.06 | 0.12 | 0.00 | 0.09 | 0.06 | 0.01 | 0.00 | 0.01 |
| 245 | 0.04 | 0.13 | 0.08 | 0.00 | 0.00 | 0.05 | 0.15 | 0.12 | 0.06 | 0.04 | 0.11 | 0.23 | 0.16 |
| 249 | 0.00 | 0.00 | 0.00 | 0.00 | 0.00 | 0.00 | 0.00 | 0.00 | 0.00 | 0.00 | 0.00 | 0.01 | 0.00 |
| 251 | 0.00 | 0.00 | 0.00 | 0.06 | 0.00 | 0.00 | 0.00 | 0.00 | 0.00 | 0.00 | 0.00 | 0.00 | 0.00 |
| 252 | 0.06 | 0.00 | 0.03 | 0.00 | 0.00 | 0.00 | 0.00 | 0.07 | 0.04 | 0.00 | 0.00 | 0.00 | 0.00 |
| 265 | 0.04 | 0.04 | 0.03 | 0.00 | 0.00 | 0.00 | 0.02 | 0.00 | 0.09 | 0.08 | 0.01 | 0.13 | 0.11 |
| 267 | 0.00 | 0.00 | 0.00 | 0.00 | 0.00 | 0.00 | 0.05 | 0.05 | 0.04 | 0.03 | 0.00 | 0.02 | 0.00 |
| G10J |  |  |  |  |  |  |  |  |  |  |  |  |  |
| 185 | 0.00 | 0.00 | 0.00 | 0.33 | 0.14 | 0.00 | 0.00 | 0.00 | 0.04 | 0.01 | 0.00 | 0.01 | 0.03 |
| 187 | 0.03 | 0.00 | 0.03 | 0.28 | 0.36 | 0.00 | 0.00 | 0.41 | 0.09 | 0.07 | 0.02 | 0.08 | 0.10 |
| 189 | 0.00 | 0.00 | 0.02 | 0.00 | 0.27 | 0.00 | 0.02 | 0.02 | 0.00 | 0.00 | 0.02 | 0.00 | 0.00 |
| 195 | 0.31 | 0.16 | 0.08 | 0.00 | 0.00 | 0.92 | 0.46 | 0.00 | 0.14 | 0.11 | 0.27 | 0.17 | 0.01 |
| 197 | 0.17 | 0.08 | 0.15 | 0.00 | 0.00 | 0.06 | 0.17 | 0.36 | 0.18 | 0.12 | 0.07 | 0.08 | 0.11 |
| 199 | 0.00 | 0.00 | 0.00 | 0.00 | 0.00 | 0.02 | 0.02 | 0.02 | 0.00 | 0.00 | 0.00 | 0.00 | 0.00 |
| 201 | 0.00 | 0.00 | 0.00 | 0.00 | 0.00 | 0.00 | 0.00 | 0.00 | 0.00 | 0.00 | 0.00 | 0.00 | 0.01 |
| 203 | 0.40 | 0.48 | 0.50 | 0.39 | 0.09 | 0.00 | 0.32 | 0.12 | 0.34 | 0.50 | 0.50 | 0.22 | 0.23 |
| 205 | 0.00 | 0.19 | 0.14 | 0.00 | 0.14 | 0.00 | 0.03 | 0.07 | 0.21 | 0.18 | 0.09 | 0.08 | 0.07 |
| 207 | 0.09 | 0.10 | 0.09 | 0.00 | 0.00 | 0.00 | 0.00 | 0.00 | 0.01 | 0.02 | 0.03 | 0.35 | 0.43 |
| G10L |  |  |  |  |  |  |  |  |  |  |  |  |  |
| 135 | 0.00 | 0.00 | 0.00 | 0.22 | 0.09 | 0.02 | 0.00 | 0.00 | 0.03 | 0.01 | 0.00 | 0.00 | 0.00 |
| 137 | 0.07 | 0.16 | 0.24 | 0.00 | 0.00 | 0.02 | 0.15 | 0.07 | 0.13 | 0.11 | 0.16 | 0.07 | 0.14 |
| 139 | 0.00 | 0.00 | 0.00 | 0.72 | 0.46 | 0.09 | 0.06 | 0.00 | 0.00 | 0.00 | 0.03 | 0.00 | 0.00 |
| 141 | 0.56 | 0.13 | 0.26 | 0.00 | 0.00 | 0.15 | 0.08 | 0.07 | 0.18 | 0.25 | 0.16 | 0.05 | 0.00 |
| 145 | 0.16 | 0.32 | 0.20 | 0.00 | 0.00 | 0.11 | 0.02 | 0.02 | 0.21 | 0.17 | 0.11 | 0.28 | 0.17 |
| 149 | 0.03 | 0.00 | 0.03 | 0.06 | 0.14 | 0.00 | 0.00 | 0.00 | 0.00 | 0.00 | 0.00 | 0.00 | 0.00 |
| 157 | 0.00 | 0.00 | 0.00 | 0.00 | 0.09 | 0.00 | 0.00 | 0.00 | 0.00 | 0.00 | 0.00 | 0.00 | 0.00 |
| 159 | 0.10 | 0.24 | 0.06 | 0.00 | 0.23 | 0.38 | 0.27 | 0.74 | 0.20 | 0.28 | 0.27 | 0.41 | 0.36 |
| 161 | 0.07 | 0.06 | 0.00 | 0.00 | 0.00 | 0.00 | 0.00 | 0.00 | 0.02 | 0.00 | 0.03 | 0.00 | 0.01 |
| 167 | 0.01 | 0.00 | 0.02 | 0.00 | 0.00 | 0.00 | 0.00 | 0.05 | 0.02 | 0.00 | 0.08 | 0.05 | 0.13 |
| 169 | 0.00 | 0.10 | 0.20 | 0.00 | 0.00 | 0.24 | 0.42 | 0.05 | 0.21 | 0.19 | 0.17 | 0.15 | 0.19 |
| G10M |  |  |  |  |  |  |  |  |  |  |  |  |  |
| 206 | 0.00 | 0.00 | 0.00 | 0.00 | 0.00 | 0.00 | 0.03 | 0.14 | 0.00 | 0.00 | 0.02 | 0.00 | 0.00 |
| 210 | 0.19 | 0.01 | 0.18 | 0.28 | 0.27 | 0.14 | 0.29 | 0.31 | 0.14 | 0.19 | 0.08 | 0.00 | 0.17 |
| 211 | 0.00 | 0.00 | 0.00 | 0.00 | 0.00 | 0.00 | 0.00 | 0.00 | 0.01 | 0.00 | 0.00 | 0.00 | 0.00 |
| 212 | 0.44 | 0.31 | 0.26 | 0.11 | 0.50 | 0.86 | 0.53 | 0.36 | 0.37 | 0.40 | 0.37 | 0.41 | 0.31 |
| 213 | 0.27 | 0.54 | 0.42 | 0.00 | 0.00 | 0.00 | 0.11 | 0.10 | 0.22 | 0.20 | 0.29 | 0.26 | 0.40 |
| 214 | 0.10 | 0.14 | 0.11 | 0.00 | 0.05 | 0.00 | 0.05 | 0.07 | 0.26 | 0.19 | 0.24 | 0.17 | 0.10 |
| 216 | 0.00 | 0.00 | 0.00 | 0.61 | 0.18 | 0.00 | 0.00 | 0.02 | 0.00 | 0.01 | 0.00 | 0.16 | 0.01 |
| 217 | 0.00 | 0.00 | 0.03 | 0.00 | 0.00 | 0.00 | 0.00 | 0.00 | 0.00 | 0.00 | 0.00 | 0.00 | 0.00 |
| G10O |  |  |  |  |  |  |  |  |  |  |  |  |  |
| 150 | 0.00 | 0.00 | 0.00 | 0.00 | 0.18 | 0.00 | 0.00 | 0.00 | 0.00 | 0.00 | 0.00 | 0.00 | 0.00 |
| 154 | 0.01 | 0.01 | 0.06 | 0.00 | 0.00 | 0.03 | 0.05 | 0.02 | 0.11 | 0.09 | 0.00 | 0.01 | 0.00 |
| 156 | 0.00 | 0.00 | 0.02 | 0.00 | 0.00 | 0.00 | 0.00 | 0.00 | 0.00 | 0.00 | 0.00 | 0.00 | 0.00 |
| 180 | 0.00 | 0.00 | 0.00 | 0.00 | 0.09 | 0.00 | 0.00 | 0.00 | 0.00 | 0.00 | 0.00 | 0.00 | 0.00 |
| 192 | 0.00 | 0.00 | 0.00 | 0.78 | 0.23 | 0.00 | 0.00 | 0.00 | 0.00 | 0.00 | 0.00 | 0.00 | 0.00 |
| 194 | 0.00 | 0.00 | 0.00 | 0.00 | 0.00 | 0.00 | 0.00 | 0.00 | 0.07 | 0.07 | 0.00 | 0.00 | 0.06 |
| 196 | 0.81 | 0.87 | 0.79 | 0.00 | 0.00 | 0.94 | 0.96 | 0.57 | 0.72 | 0.81 | 0.92 | 0.94 | 0.89 |
| 198 | 0.14 | 0.06 | 0.14 | 0.00 | 0.09 | 0.00 | 0.00 | 0.41 | 0.07 | 0.03 | 0.00 | 0.02 | 0.01 |
| 204 | 0.00 | 0.00 | 0.00 | 0.00 | 0.05 | 0.00 | 0.00 | 0.00 | 0.00 | 0.00 | 0.00 | 0.00 | 0.00 |
| 208 | 0.03 | 0.06 | 0.00 | 0.06 | 0.18 | 0.00 | 0.00 | 0.00 | 0.03 | 0.00 | 0.00 | 0.02 | 0.04 |
| 210 | 0.00 | 0.00 | 0.00 | 0.17 | 0.18 | 0.03 | 0.00 | 0.00 | 0.00 | 0.00 | 0.07 | 0.00 | 0.00 |
| G10P |  |  |  |  |  |  |  |  |  |  |  |  |  |
| 147 | 0.00 | 0.00 | 0.00 | 0.06 | 0.32 | 0.00 | 0.00 | 0.00 | 0.05 | 0.00 | 0.00 | 0.00 | 0.00 |
| 151 | 0.00 | 0.00 | 0.00 | 0.06 | 0.27 | 0.00 | 0.00 | 0.00 | 0.00 | 0.00 | 0.00 | 0.00 | 0.00 |
| 153 | 0.70 | 0.86 | 0.71 | 0.00 | 0.00 | 0.59 | 0.64 | 0.55 | 0.63 | 0.87 | 0.71 | 0.70 | 0.74 |
| 155 | 0.13 | 0.01 | 0.00 | 0.00 | 0.00 | 0.00 | 0.00 | 0.00 | 0.01 | 0.00 | 0.02 | 0.00 | 0.00 |
| 157 | 0.04 | 0.06 | 0.15 | 0.17 | 0.00 | 0.00 | 0.00 | 0.00 | 0.01 | 0.02 | 0.01 | 0.00 | 0.01 |
| 159 | 0.13 | 0.04 | 0.06 | 0.39 | 0.14 | 0.08 | 0.14 | 0.29 | 0.17 | 0.07 | 0.05 | 0.27 | 0.23 |
| 161 | 0.00 | 0.02 | 0.08 | 0.00 | 0.00 | 0.05 | 0.05 | 0.17 | 0.01 | 0.00 | 0.17 | 0.01 | 0.00 |
| 163 | 0.00 | 0.00 | 0.00 | 0.33 | 0.27 | 0.23 | 0.06 | 0.00 | 0.05 | 0.03 | 0.05 | 0.00 | 0.00 |
| 165 | 0.00 | 0.00 | 0.00 | 0.00 | 0.00 | 0.06 | 0.12 | 0.00 | 0.03 | 0.02 | 0.00 | 0.02 | 0.00 |
| 167 | 0.00 | 0.01 | 0.00 | 0.00 | 0.00 | 0.00 | 0.00 | 0.00 | 0.05 | 0.00 | 0.00 | 0.00 | 0.01 |
| G10U |  |  |  |  |  |  |  |  |  |  |  |  |  |
| 161 | 0.00 | 0.24 | 0.15 | 0.00 | 0.00 | 0.02 | 0.00 | 0.00 | 0.00 | 0.00 | 0.01 | 0.02 | 0.00 |
| 163 | 0.04 | 0.01 | 0.08 | 0.00 | 0.00 | 0.02 | 0.05 | 0.02 | 0.05 | 0.00 | 0.11 | 0.00 | 0.01 |
| 165 | 0.51 | 0.29 | 0.50 | 0.00 | 0.00 | 0.14 | 0.18 | 0.17 | 0.44 | 0.46 | 0.30 | 0.27 | 0.09 |
| 167 | 0.21 | 0.02 | 0.17 | 0.00 | 0.00 | 0.68 | 0.73 | 0.33 | 0.32 | 0.25 | 0.42 | 0.19 | 0.09 |
| 169 | 0.04 | 0.25 | 0.00 | 0.00 | 0.00 | 0.15 | 0.02 | 0.00 | 0.01 | 0.02 | 0.02 | 0.27 | 0.21 |
| 171 | 0.00 | 0.00 | 0.02 | 0.00 | 0.00 | 0.00 | 0.00 | 0.00 | 0.00 | 0.00 | 0.00 | 0.00 | 0.00 |
| 173 | 0.04 | 0.10 | 0.02 | 0.00 | 0.00 | 0.00 | 0.00 | 0.00 | 0.00 | 0.00 | 0.00 | 0.07 | 0.10 |
| 175 | 0.00 | 0.00 | 0.00 | 0.11 | 0.46 | 0.00 | 0.02 | 0.00 | 0.00 | 0.01 | 0.10 | 0.11 | 0.00 |
| 177 | 0.11 | 0.10 | 0.05 | 0.89 | 0.41 | 0.00 | 0.02 | 0.48 | 0.19 | 0.26 | 0.05 | 0.08 | 0.49 |
| 179 | 0.03 | 0.00 | 0.03 | 0.00 | 0.00 | 0.00 | 0.00 | 0.00 | 0.00 | 0.00 | 0.00 | 0.00 | 0.01 |
| 181 | 0.00 | 0.00 | 0.00 | 0.00 | 0.14 | 0.00 | 0.00 | 0.00 | 0.00 | 0.00 | 0.00 | 0.00 | 0.00 |
| G10X |  |  |  |  |  |  |  |  |  |  |  |  |  |
| 133 | 0.00 | 0.00 | 0.00 | 0.00 | 0.00 | 0.00 | 0.00 | 0.19 | 0.00 | 0.00 | 0.00 | 0.00 | 0.00 |
| 139 | 0.00 | 0.00 | 0.03 | 0.00 | 0.09 | 0.00 | 0.00 | 0.00 | 0.01 | 0.01 | 0.00 | 0.00 | 0.00 |
| 141 | 0.04 | 0.04 | 0.02 | 0.06 | 0.23 | 0.06 | 0.05 | 0.17 | 0.13 | 0.08 | 0.04 | 0.00 | 0.00 |
| 143 | 0.00 | 0.02 | 0.02 | 0.00 | 0.00 | 0.00 | 0.00 | 0.00 | 0.00 | 0.00 | 0.01 | 0.00 | 0.00 |
| 145 | 0.37 | 0.39 | 0.29 | 0.06 | 0.14 | 0.38 | 0.23 | 0.24 | 0.30 | 0.33 | 0.21 | 0.30 | 0.31 |
| 147 | 0.27 | 0.43 | 0.50 | 0.17 | 0.00 | 0.56 | 0.64 | 0.36 | 0.27 | 0.41 | 0.64 | 0.55 | 0.50 |
| 149 | 0.09 | 0.00 | 0.05 | 0.00 | 0.00 | 0.00 | 0.09 | 0.02 | 0.00 | 0.00 | 0.10 | 0.02 | 0.13 |
| 151 | 0.23 | 0.12 | 0.11 | 0.00 | 0.00 | 0.00 | 0.00 | 0.02 | 0.30 | 0.17 | 0.01 | 0.13 | 0.06 |
| 153 | 0.00 | 0.00 | 0.00 | 0.17 | 0.05 | 0.00 | 0.00 | 0.00 | 0.00 | 0.00 | 0.00 | 0.00 | 0.00 |
| 155 | 0.00 | 0.00 | 0.00 | 0.00 | 0.23 | 0.00 | 0.00 | 0.00 | 0.00 | 0.00 | 0.00 | 0.00 | 0.00 |
| 157 | 0.00 | 0.00 | 0.00 | 0.50 | 0.27 | 0.00 | 0.00 | 0.00 | 0.00 | 0.00 | 0.00 | 0.00 | 0.00 |
| 159 | 0.00 | 0.00 | 0.00 | 0.06 | 0.00 | 0.00 | 0.00 | 0.00 | 0.00 | 0.00 | 0.00 | 0.00 | 0.00 |
| MU50 |  |  |  |  |  |  |  |  |  |  |  |  |  |
| 120 | 0.00 | 0.00 | 0.00 | 0.39 | 0.14 | 0.00 | 0.00 | 0.00 | 0.00 | 0.00 | 0.00 | 0.00 | 0.00 |
| 122 | 0.86 | 0.93 | 1.00 | 0.00 | 0.14 | 1.00 | 0.99 | 0.98 | 0.90 | 0.99 | 0.90 | 1.00 | 0.89 |
| 124 | 0.00 | 0.00 | 0.00 | 0.00 | 0.18 | 0.00 | 0.00 | 0.00 | 0.01 | 0.00 | 0.00 | 0.00 | 0.01 |
| 126 | 0.00 | 0.00 | 0.00 | 0.17 | 0.23 | 0.00 | 0.00 | 0.00 | 0.00 | 0.00 | 0.02 | 0.00 | 0.00 |
| 132 | 0.00 | 0.00 | 0.00 | 0.06 | 0.00 | 0.00 | 0.00 | 0.00 | 0.05 | 0.01 | 0.00 | 0.00 | 0.00 |
| 134 | 0.14 | 0.07 | 0.00 | 0.00 | 0.00 | 0.00 | 0.02 | 0.02 | 0.01 | 0.00 | 0.08 | 0.00 | 0.10 |
| 136 | 0.00 | 0.00 | 0.00 | 0.00 | 0.05 | 0.00 | 0.00 | 0.00 | 0.00 | 0.00 | 0.00 | 0.00 | 0.00 |
| 138 | 0.00 | 0.00 | 0.00 | 0.06 | 0.14 | 0.00 | 0.00 | 0.00 | 0.00 | 0.00 | 0.00 | 0.00 | 0.00 |
| 140 | 0.00 | 0.00 | 0.00 | 0.33 | 0.09 | 0.00 | 0.00 | 0.00 | 0.04 | 0.00 | 0.00 | 0.00 | 0.00 |
| 144 | 0.00 | 0.00 | 0.00 | 0.00 | 0.05 | 0.00 | 0.00 | 0.00 | 0.00 | 0.00 | 0.00 | 0.00 | 0.00 |
| MU59 |  |  |  |  |  |  |  |  |  |  |  |  |  |
| 231 | 0.87 | 0.54 | 0.52 | 0.00 | 0.00 | 0.76 | 0.77 | 0.31 | 0.39 | 0.58 | 0.45 | 0.56 | 0.80 |
| 235 | 0.00 | 0.00 | 0.00 | 0.00 | 0.00 | 0.03 | 0.08 | 0.10 | 0.00 | 0.00 | 0.08 | 0.20 | 0.00 |
| 237 | 0.06 | 0.23 | 0.14 | 0.00 | 0.00 | 0.00 | 0.00 | 0.10 | 0.05 | 0.02 | 0.01 | 0.06 | 0.00 |
| 239 | 0.00 | 0.10 | 0.06 | 1.00 | 0.59 | 0.00 | 0.00 | 0.00 | 0.12 | 0.05 | 0.00 | 0.00 | 0.04 |
| 241 | 0.01 | 0.07 | 0.14 | 0.00 | 0.09 | 0.03 | 0.06 | 0.17 | 0.08 | 0.08 | 0.05 | 0.07 | 0.00 |
| 243 | 0.06 | 0.07 | 0.12 | 0.00 | 0.32 | 0.00 | 0.03 | 0.07 | 0.21 | 0.12 | 0.29 | 0.12 | 0.04 |
| 245 | 0.00 | 0.00 | 0.03 | 0.00 | 0.00 | 0.18 | 0.06 | 0.02 | 0.16 | 0.15 | 0.12 | 0.00 | 0.11 |
| 247 | 0.00 | 0.00 | 0.00 | 0.00 | 0.00 | 0.00 | 0.00 | 0.24 | 0.00 | 0.00 | 0.00 | 0.00 | 0.00 |

Table S13. Estimated pairwise genetic differentiation (F_ST_) and their 95% confidence intervals based on 1,000 bootstrap iterations for structure identified clusters of American black bears (*Ursus americanus*) in the American Southwest and northern Mexico. Bolded values signify statistically significant differentiation (FST ≥ 0.05; Hartl & Clark, 1997).

| Cluster | 1.1 | 1.2 | 1.3 | 2.1 | 2.2 | 3.1 | 3.2 | 4 | 5.1 | 5.2 | 6 | 7.1 | 7.2 |
| --- | --- | --- | --- | --- | --- | --- | --- | --- | --- | --- | --- | --- | --- |
| 1.1 | -- |  |  |  |  |  |  |  |  |  |  |  |  |
| 1.2 | 0.04 | -- |  |  |  |  |  |  |  |  |  |  |  |
| 1.3 | 0.03 | 0.02 | -- |  |  |  |  |  |  |  |  |  |  |
| 2.1 | 0.20 | 0.18 | 0.19 | -- |  |  |  |  |  |  |  |  |  |
| 2.2 | 0.18 | 0.17 | 0.17 | 0.09 | -- |  |  |  |  |  |  |  |  |
| 3.1 | 0.09 | 0.11 | 0.10 | 0.29 | 0.25 | -- |  |  |  |  |  |  |  |
| 3.2 | 0.05 | 0.07 | 0.05 | 0.24 | 0.21 | 0.05 | -- |  |  |  |  |  |  |
| 4 | 0.10 | 0.10 | 0.09 | 0.21 | 0.16 | 0.17 | 0.12 | -- |  |  |  |  |  |
| 5.1 | 0.03 | 0.03 | 0.03 | 0.12 | 0.10 | 0.08 | 0.05 | 0.06 | -- |  |  |  |  |
| 5.2 | 0.03 | 0.04 | 0.03 | 0.15 | 0.14 | 0.09 | 0.05 | 0.08 | 0.01 | -- |  |  |  |
| 6 | 0.03 | 0.03 | 0.02 | 0.08 | 0.08 | 0.05 | 0.02 | 0.06 | 0.03 | 0.03 | -- |  |  |
| 7.1 | 0.05 | 0.03 | 0.04 | 0.18 | 0.17 | 0.08 | 0.05 | 0.10 | 0.03 | 0.04 | 0.03 | -- |  |
| 7.2 | 0.06 | 0.05 | 0.05 | 0.18 | 0.17 | 0.11 | 0.07 | 0.09 | 0.05 | 0.05 | 0.04 | 0.04 | -- |

Table S14. Estimated pairwise-relative migration rates for structure identified clusters of American black bears (*Ursus americanus*) in the American Southwest and northern Mexico. Bolded values indicate significant asymmetric migration based on 1,000 bootstrap iterations. Table is interpreted as migration occurring from the cluster in the left-hand column to the cluster in the top row.

| Cluster | 1.1 | 1.2 | 1.3 | 2.1 | 2.2 | 3.1 | 3.2 | 4 | 5.1 | 5.2 | 6 | 7.1 | 7.2 |
| --- | --- | --- | --- | --- | --- | --- | --- | --- | --- | --- | --- | --- | --- |
| 1.1 | -- | 0.21 | 0.29 | 0.01 | 0.01 | 0.04 | 0.11 | 0.07 | 0.31 | 0.23 | 0.16 | 0.13 | 0.09 |
| 1.2 | 0.17 | -- | 0.27 | 0.00 | 0.01 | 0.03 | 0.07 | 0.07 | 0.21 | 0.17 | 0.15 | 0.22 | 0.13 |
| 1.3 | 0.21 | 0.33 | -- | 0.01 | 0.01 | 0.03 | 0.10 | 0.10 | 0.28 | 0.22 | 0.16 | 0.17 | 0.13 |
| 2.1 | 0.01 | 0.01 | 0.01 | -- | 0.11 | 0.01 | 0.01 | 0.01 | 0.01 | 0.01 | 0.01 | 0.01 | 0.01 |
| 2.2 | 0.01 | 0.01 | 0.01 | 0.04 | -- | 0.01 | 0.02 | 0.01 | 0.02 | 0.01 | 0.01 | 0.01 | 0.01 |
| 3.1 | 0.05 | 0.05 | **0.08** | 0.01 | 0.01 | -- | 0.19 | 0.02 | **0.12** | **0.08** | **0.11** | **0.08** | **0.06** |
| 3.2 | 0.08 | 0.07 | 0.12 | 0.01 | 0.01 | 0.11 | -- | 0.05 | 0.17 | 0.13 | 0.19 | 0.13 | 0.10 |
| 4 | 0.05 | 0.04 | 0.06 | 0.01 | 0.01 | 0.02 | 0.03 | -- | 0.07 | 0.05 | 0.04 | 0.05 | 0.04 |
| 5.1 | 0.14 | 0.16 | 0.23 | 0.01 | 0.01 | 0.04 | 0.09 | 0.09 | -- | 0.57 | 0.13 | 0.15 | 0.12 |
| 5.2 | 0.19 | 0.18 | 0.21 | 0.01 | 0.01 | 0.03 | 0.11 | 0.09 | **1.00** | -- | 0.18 | 0.15 | 0.12 |
| 6 | 0.14 | 0.19 | 0.23 | 0.00 | 0.01 | 0.04 | 0.20 | 0.07 | **0.26** | 0.20 | -- | 0.23 | 0.14 |
| 7.1 | 0.13 | 0.19 | 0.13 | 0.01 | 0.01 | 0.03 | 0.09 | 0.06 | 0.19 | 0.17 | 0.15 | -- | 0.17 |
| 7.2 | 0.10 | 0.10 | 0.12 | 0.01 | 0.01 | 0.03 | 0.05 | 0.05 | 0.13 | 0.09 | 0.09 | 0.15 | -- |

## Appendix S2. Global results


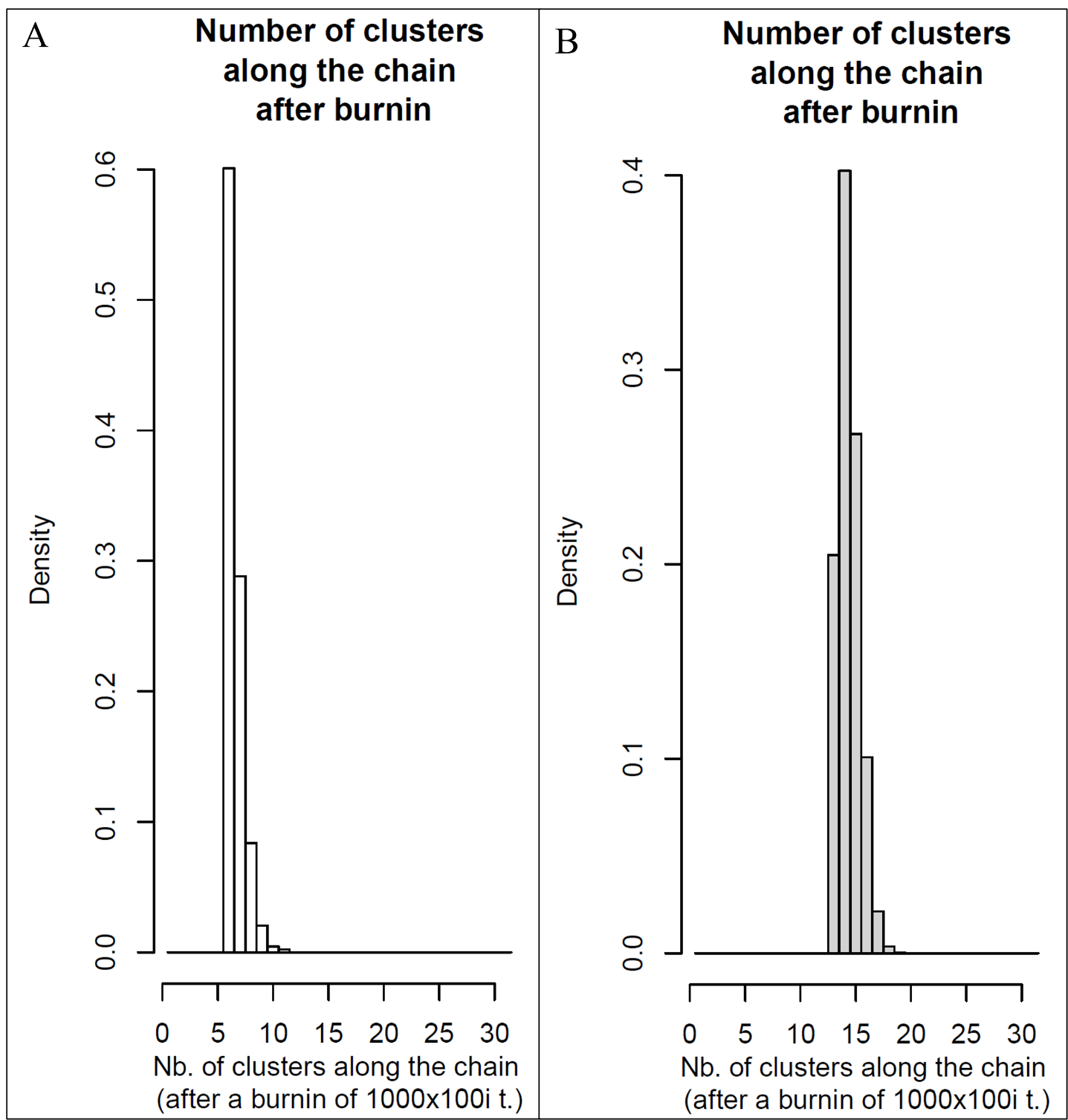


Figure S1. The number of subpopulations identified by geneland using the uncorrelated (A) and correlated (B) allele frequency models.

Table S1**.** Number of individuals genotyped to 15 microsatellite loci and a sex marker used in our analysis by state and sampling locale.

| Location | State, Country | Male | Female | Total |
| --- | --- | --- | --- | --- |
| Chiricahua complex | Arizona, USA | 6 | 9 | 15 |
| Galiuro and Winchester mountains | Arizona, USA | 8 | 6 | 14 |
| Huachuca Mountains | Arizona, USA | 8 | 10 | 18 |
| Pinaleno Mountains | Arizona, USA | 6 | 6 | 12 |
| Prescott Region | Arizona, USA | 13 | 17 | 30 |
| Southern Gila and northern Peloncillo | Arizona, USA | 4 | 0 | 4 |
| Santa Catalina Mountains | Arizona, USA | 1 | 0 | 1 |
| Santa Rita Mountains | Arizona, USA | 14 | 7 | 21 |
| Santa Teresa Mountains | Arizona, USA | 1 | 1 | 2 |
| Whetstone Mountains | Arizona, USA | 1 | 0 | 1 |
| White Mountains Region | Arizona, USA | 11 | 14 | 25 |
| Sangre de Cristo Mountains | Colorado, USA | 16 | 15 | 31 |
| San Juan Mountains | Colorado, USA | 16 | 14 | 30 |
| Chuska Mountains | New Mexico, USA | 2 | 1 | 3 |
| Gila complex | New Mexico, USA | 25 | 28 | 53 |
| Mt. Taylor | New Mexico, USA | 15 | 12 | 27 |
| Sacramento Complex | New Mexico, USA | 16 | 13 | 29 |
| San Andres and Oscura mountains | New Mexico, USA | 2 | 1 | 3 |
| Sandia and Manzano mountains | New Mexico, USA | 16 | 13 | 29 |
| Sangre de Cristo Mountains | New Mexico, USA | 28 | 25 | 53 |
| San Juan Mountains | New Mexico, USA | 21 | 29 | 50 |
| Zuni Mountains | New Mexico, USA | 17 | 13 | 30 |
| Big Bend National Park | Texas, USA | 6 | 6 | 12 |
| Black Gap Wildlife Management Area | Texas, USA | 3 | 1 | 4 |
| Gladys Porter Zoo, captured in Zapata, TX | Texas, USA | 1 | 0 | 1 |
| Boulder Mountain | Utah, USA | 14 | 7 | 21 |
| LaSal Mountains | Utah, USA | 14 | 14 | 28 |
| Serranias del Burro | Coahuila de Zaragoza, Mexico | 0 | 3 | 3 |
|  |  | 285 | 265 | 550 |

Table S2. Frequency of null alleles and its 95% confidence interval for the global population of American black bears (*Ursus americanus*) in the American Southwest and northern Mexico. Bolded values indicate the 95% confidence interval for the allele does not overlap 0.

| Locus | Global |
| --- | --- |
| CXX20 | **0.10 (0.07–0.13)** |
| G1A | **0.06 (0.03–0.10)** |
| G1D | **0.04 (0.01–0.06)** |
| G10B | **0.05 (0.02–0.08)** |
| G10C | **0.09 (0.05–0.14)** |
| G10H | **0.09 (0.06–0.11)** |
| G10J | **0.08 (0.05–0.10)** |
| G10L | **0.04 (0.02–0.07)** |
| G10M | **0.05 (0.02–0.08)** |
| G100 | **0.12 (0.07–0.17)** |
| G10P | **0.04 (0.01–0.07)** |
| G10U | **0.06 (0.03–0.09)** |
| G10X | **0.04 (0.01–0.06)** |
| MU50 | **0.10 (0.05–0.15)** |
| MU59 | **0.06 (0.03–0.09)** |

Table S3. Estimated linkage disequilibrium for 15 loci used to characterize the genetic structure for the global population of American black bears (*Ursus americanus*) in the American Southwest and northern Mexico. Bolded values indicate a statistically significant test after applying a Bonferroni correction of α < 0.0005.

|  | CXX20 | G1A | G1D | G10B | G10C | G10H | G10J | G10L | G10M | G10O | G10P | G10U | G10X | MU50 | MU59 |
| --- | --- | --- | --- | --- | --- | --- | --- | --- | --- | --- | --- | --- | --- | --- | --- |
| CXX20 | -- |  |  |  |  |  |  |  |  |  |  |  |  |  |  |
| G1A | **0.00** | -- |  |  |  |  |  |  |  |  |  |  |  |  |  |
| G1D | **0.00** | 0.05 | -- |  |  |  |  |  |  |  |  |  |  |  |  |
| G10B | **0.00** | **0.00** | 0.05 | -- |  |  |  |  |  |  |  |  |  |  |  |
| G10C | **0.00** | **0.00** | 0.00 | **0.00** | -- |  |  |  |  |  |  |  |  |  |  |
| G10H | **0.00** | **0.00** | 0.06 | **0.00** | **0.00** | -- |  |  |  |  |  |  |  |  |  |
| G10J | **0.00** | **0.00** | **0.00** | **0.00** | **0.00** | **0.00** | -- |  |  |  |  |  |  |  |  |
| G10L | 0.01 | **0.00** | 0.42 | **< 0.01** | **0.00** | **0.00** | 0.35 | -- |  |  |  |  |  |  |  |
| G10M | **0.00** | 0.01 | 0.03 | 0.01 | **0.00** | 0.01 | **0.00** | **0.00** | -- |  |  |  |  |  |  |
| G10O | **0.00** | **0.00** | **0.00** | **0.00** | **0.00** | **0.00** | **0.00** | **0.00** | **<0.01** | -- |  |  |  |  |  |
| G10P | **0.00** | **0.00** | **0.00** | **0.00** | **0.00** | 0.03 | **0.00** | **0.00** | **0.00** | **0.00** | -- |  |  |  |  |
| G10U | **0.00** | **0.00** | 0.08 | **0.00** | **0.00** | **0.00** | **0.00** | **0.00** | **0.00** | **0.00** | **0.00** | -- |  |  |  |
| G10X | **0.00** | **0.00** | 0.00 | **0.00** | **0.00** | 0.01 | **0.00** | **0.00** | **0.00** | **0.00** | **0.00** | < 0.01 | -- |  |  |
| MU50 | **0.00** | **0.00** | 0.03 | **0.00** | **0.00** | **0.00** | **0.00** | **0.00** | **0.00** | **0.00** | **0.00** | **0.00** | **0.00** | -- |  |
| MU59 | **0.00** | **0.00** | 0.07 | **0.00** | **0.00** | **0.00** | **0.00** | < 0.01 | **0.00** | **0.00** | **0.00** | **0.00** | **0.00** | **0.00** | -- |

Table S4. Hardy-Weinberg proportion tests for 15 loci used to characterize the genetic structure for the global population of American black bears (*Ursus americanus*) in the American Southwest and northern Mexico. Bolded values indicate a statistically significant test after applying a Bonferroni correction of α < 0.003.

| Locus | Global population |
| --- | --- |
| CXX20 | 0.00 |
| G1A | 0.00 |
| G1D | 0.00 |
| G10B | 0.00 |
| G10C | 0.00 |
| G10H | 0.00 |
| G10J | 0.00 |
| G10L | 0.00 |
| G10M | 0.00 |
| G10O | 0.00 |
| G10P | 0.00 |
| G10U | 0.00 |
| G10X | 0.00 |
| MU50 | 0.00 |
| MU59 | 0.00 |

## Appendix S3. Regional subpopulation results


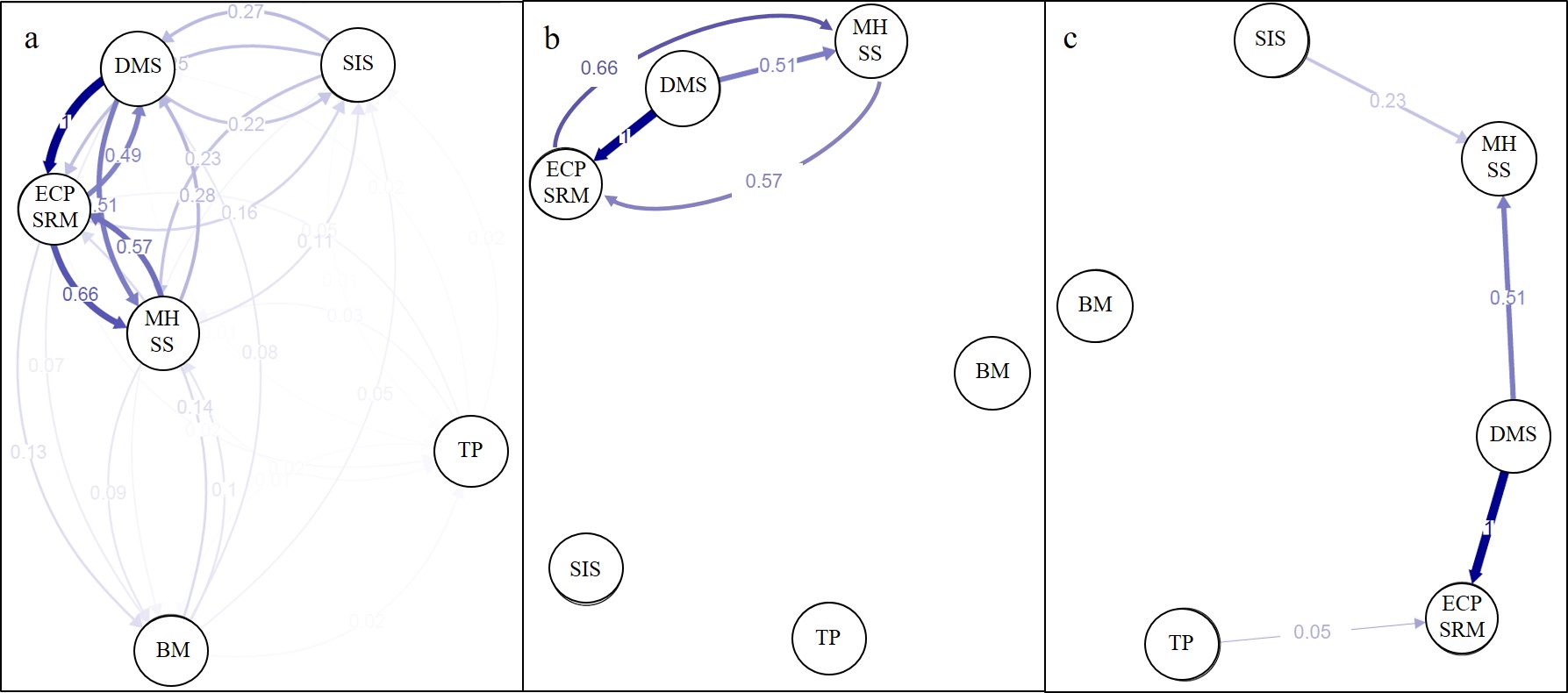


Figure S1. Directional-relative migration networks based on G_ST_ for regional subpopulations identified by geneland using the uncorrelated allele frequency model for American black bears (*Ursus americanus*) in the American Southwest and northern Mexico. Networks visualized include (a) all migration values (b) migration values ≥ 0.50, and (c) significant asymmetrical migration values. Populations visualized include Boulder Mountain (BM), the eastern Colorado Plateau and Southern Rocky Mountains (ECPSRM), the Datil-Mogollon Section (DMS), the Mexican Highland and Sacramento sections (MHSS), the Sky Islands South of Interstate-10 (SIS), and the Trans-Pecos region (TP). Significant asymmetric gene flow occurs from DMS to ECPSRM (1.0 vs. 0.49), from DMS to MHSS (0.51 vs. 0.28), from SIS to MHSS (0.23 vs 0.11), and from TP to ECPSRM (0.05 vs. 0.02).

Table C.1. Frequency of null alleles and its 95% confidence interval for regional subpopulations identified by geneland using the uncorrelated allele frequency model for American black bears (*Ursus americanus*) in the American Southwest and northern Mexico. Bolded values indicate the 95% confidence interval for the allele does not overlap 0.

| Locus | BM^a^ | ECPSRM^b^ | DMS^c^ | MHSS^d^ | SIS^e^ | TP^f^ |
| --- | --- | --- | --- | --- | --- | --- |
| CXX20 | **-0.07**  **-0.13– -0.03** | 0.04  0.00–0.09 | -0.01  -0.04–0.03 | 0.06  -0.02–0.16 | 0.07  -0.03–0.20 | -0.06  -0.13–0.01 |
| G1A | -0.05  -0.16–0.10 | -0.01  -0.04–0.03 | 0.03  -0.04–0.12 | 0.00  -0.09–0.09 | 0.06  -0.05–0.19 | -0.03  -0.13–0.11 |
| G1D | 0.07  -0.08–0.25 | 0.03  0.00–0.07 | -0.01  -0.05–0.04 | 0.07  -0.01–0.17 | -0.02  -0.09–0.05 | -0.01  -0.14–0.16 |
| G10B | 0.10  -0.04–0.26 | -0.01  -0.05–0.03 | 0.07  0.00–0.15 | 0.01  -0.06–0.08 | 0.01  -0.08–0.12 | -0.04  -0.13–0.04 |
| G10C | 0.27  0.00–0.66 | 0.02  -0.02–0.08 | -0.02  -0.06–0.04 | 0.00  -0.09–0.15 | -0.02  -0.04–0.00 | 0.01  -0.11–0.17 |
| G10H | -0.03  -0.12–0.05 | **0.05**  **0.01–0.09** | 0.01  -0.04–0.07 | 0.00  -0.06–0.07 | 0.02  -0.06–0.14 | 0.01  -0.12–0.26 |
| G10J | 0.09  -0.06–0.28 | 0.02  -0.02–0.05 | 0.03  -0.03–0.09 | 0.00  -0.06–0.08 | **0.13**  **0.01–0.28** | **-0.08**  **-0.15–-0.02** |
| G10L | -0.04  -0.11–0.05 | 0.02  -0.01–0.05 | 0.04  -0.01–0.08 | -0.04  -0.09–0.01 | 0.04  -0.04–0.12 | -0.02  -0.14–0.11 |
| G10M | -0.07  -0.16–0.03 | 0.01  -0.03–0.05 | 0.03  -0.02–0.09 | 0.05  -0.03–0.14 | 0.06  -0.05–0.21 | 0.02  -0.12–0.19 |
| G10O | -0.06  -0.22–0.15 | 0.04  -0.01–0.11 | 0.07  -0.04–0.22 | **-0.03**  **-0.05– -0.02** | **-0.02**  **-0.05– -0.01** | 0.05  -0.07–0.17 |
| G10P | 0.00  -0.12–0.16 | 0.04  0.00–0.09 | 0.01  -0.04–0.08 | -0.09  -0.16–0.00 | -0.05  -0.11–0.03 | -0.02  -0.11–0.09 |
| G10U | -0.01  -0.13–0.15 | 0.01  -0.03–0.05 | 0.02  -0.02–0.07 | 0.03  -0.03–0.10 | -0.04  -0.10–0.01 | 0.01  -0.14–0.22 |
| G10X | 0.09  -0.04–0.25 | 0.00  -0.04–0.04 | 0.01  -0.04–0.08 | 0.01  -0.07–0.11 | 0.04  -0.07–0.17 | 0.00  -0.11–0.10 |
| MU50 | **-0.01**  **-0.04– -0.01** | 0.00  -0.03–0.08 | **-0.03**  **-0.05– -0.02** | 0.08  -0.04–0.33 | NA | -0.01  -0.09–0.08 |
| MU59 | -0.04  -0.12–0.03 | 0.01  -0.03–0.04 | 0.05  -0.01–0.12 | 0.02  -0.04–0.11 | 0.02  -0.07–0.13 | -0.04  -0.14–0.07 |

^a^ Boulder Mountain (BM), ^b^ eastern Colorado Plateau and Southern Rocky Mountains (ECPSRM), ^c^ Datil-Mogollon Section (DMS), ^d^ Mexican Highland and Sacramento sections (MHSS), ^e^ Sky Islands South of Interstate 10 (SIS), ^f^ Trans-Pecos (TP).

Table C.2. Estimated linkage disequilibrium for 15 loci used to characterize the genetic structure for regional subpopulations identified by geneland using the uncorrelated allele frequency model for American black bears (*Ursus americanus*) in the American Southwest and northern Mexico. Bolded values indicate a statistically significant test after applying a Bonferroni correction of α < 0.0005. Boulder Mountain (below diagonal) and eastern Colorado Plateau and Southern Rocky Mountains (above diagonal).

| Locus | CXX20 | G10B | G10C | G10H | G10J | G10L | G10M | G10O | G10P | G10U | G10X | G1A | G1D | MU50 | MU59 |
| --- | --- | --- | --- | --- | --- | --- | --- | --- | --- | --- | --- | --- | --- | --- | --- |
| CXX20 | -- | 0.52 | 0.19 | 0.39 | 0.24 | 0.18 | 0.22 | 0.16 | 0.59 | 0.48 | 0.12 | 0.20 | 0.00 | 0.18 | 0.51 |
| G10B | **<0.01** | -- | 0.29 | 0.24 | 0.09 | 0.13 | 0.38 | 0.42 | 0.11 | 0.55 | 0.87 | 0.80 | 0.51 | 0.06 | 0.49 |
| G10C | 0.41 | 0.40 | -- | 0.84 | 0.30 | 0.01 | 0.62 | 0.70 | 0.89 | 0.95 | 0.82 | 0.63 | 0.50 | 0.04 | 0.12 |
| G10H | **<0.01** | 0.61 | 0.96 | -- | 0.43 | 0.09 | 0.83 | 0.85 | 0.61 | 0.12 | 0.92 | 0.31 | 0.69 | 0.07 | 0.22 |
| G10J | 0.02 | 0.84 | 0.74 | 0.02 | -- | 0.95 | 0.44 | 0.22 | 0.86 | 0.45 | 0.28 | 0.32 | 0.51 | 0.80 | 0.38 |
| G10L | 0.19 | 0.84 | 0.54 | 0.16 | 0.28 | -- | 0.61 | 0.91 | 0.41 | 0.17 | 0.22 | 0.63 | 0.93 | 0.20 | 0.70 |
| G10M | 0.11 | 0.62 | 0.79 | 0.05 | 0.10 | 0.42 | -- | 0.39 | 0.50 | 0.05 | 0.15 | 0.69 | 0.43 | 0.75 | 0.80 |
| G10O | 0.04 | 0.95 | 0.13 | 0.03 | 0.52 | 0.56 | 0.24 | -- | 0.52 | 0.68 | 0.50 | 0.13 | 0.22 | 0.13 | 0.06 |
| G10P | 0.29 | 0.21 | 0.17 | 0.23 | 0.16 | 0.48 | 0.32 | 0.27 | -- | 0.79 | 0.89 | 0.07 | 0.35 | 0.16 | 0.37 |
| G10U | 0.87 | 0.19 | 0.99 | 0.05 | 0.98 | 0.32 | 0.49 | 0.78 | 0.79 | -- | 0.58 | 0.96 | 0.41 | 0.85 | 0.70 |
| G10X | 0.89 | 0.94 | 0.46 | 0.98 | 0.89 | 0.27 | 0.97 | 0.75 | 0.89 | 0.95 | -- | 0.67 | 0.12 | 0.45 | 0.69 |
| G1A | 0.33 | 0.42 | 0.60 | 0.46 | 0.27 | 0.32 | 0.13 | 0.13 | 0.19 | 1.00 | 0.21 | -- | 0.84 | 0.92 | 0.08 |
| G1D | 0.45 | 0.63 | 0.43 | 0.52 | 0.81 | 0.09 | 0.04 | 0.05 | 0.69 | 0.90 | 1.00 | 0.16 | -- | 0.53 | 0.51 |
| MU50 | 0.10 | 0.38 | 0.62 | 0.19 | 1.00 | 0.48 | 0.62 | 0.48 | 0.16 | 0.76 | 1.00 | 1.00 | 0.38 | -- | 0.21 |
| MU59 | 0.02 | 0.12 | 0.67 | 0.15 | 0.01 | 0.78 | 0.51 | 0.81 | 0.37 | 0.19 | 0.41 | 0.94 | 0.64 | 1.00 | -- |

Table C.3. Estimated linkage disequilibrium for 15 loci used to characterize the genetic structure for regional subpopulations identified by geneland using the uncorrelated allele frequency model for American black bears (*Ursus americanus*) in the American Southwest and northern Mexico. Bolded values indicate a statistically significant test after applying a Bonferroni correction of α < 0.0005. Datil-Mogollon Section (below diagonal) and Mexican Highland and Sacramento sections (above diagonal).

| Locus | CXX20 | G10B | G10C | G10H | G10J | G10L | G10M | G10O | G10P | G10U | G10X | G1A | G1D | MU50 | MU59 |
| --- | --- | --- | --- | --- | --- | --- | --- | --- | --- | --- | --- | --- | --- | --- | --- |
| CXX20 | -- | 0.01 | 0.57 | 0.02 | 0.51 | 0.14 | 0.95 | 0.02 | 0.71 | 0.12 | 0.13 | 0.27 | 0.63 | 0.01 | 0.32 |
| G10B | 0.94 | -- | 0.14 | 0.68 | 0.51 | 0.44 | 0.75 | 0.01 | 0.83 | 0.01 | 0.30 | 0.30 | 0.08 | 0.41 | 0.14 |
| G10C | 0.35 | 0.70 | -- | 0.15 | 0.83 | 0.82 | 0.09 | 0.79 | 0.77 | 0.45 | 0.59 | 0.83 | 0.77 | 0.77 | 0.28 |
| G10H | 0.37 | 0.25 | 0.25 | -- | 0.20 | 0.09 | 0.41 | 0.40 | 0.08 | 0.35 | 0.99 | 0.70 | 0.08 | 0.48 | 0.31 |
| G10J | 0.38 | 0.34 | 0.12 | 0.76 | -- | 0.21 | 0.93 | 0.32 | 0.93 | 0.66 | 0.64 | 0.04 | 0.80 | 0.34 | 0.80 |
| G10L | 0.84 | 0.23 | 0.67 | 0.83 | 0.42 | -- | 0.57 | 0.20 | 0.38 | 0.96 | 0.40 | 0.70 | 0.85 | 0.42 | 0.93 |
| G10M | 0.08 | 0.15 | 0.15 | 0.13 | 0.47 | 0.66 | -- | 0.13 | 0.05 | 0.11 | 0.05 | 0.21 | 0.37 | 0.73 | 0.02 |
| G10O | 0.23 | 0.93 | 0.03 | 0.58 | 0.46 | 0.38 | 0.21 | -- | 0.17 | 0.44 | 0.02 | 0.46 | 0.78 | 0.32 | 0.27 |
| G10P | 0.58 | 0.45 | 0.65 | 0.90 | 0.04 | 0.20 | 0.76 | 0.92 | -- | 0.70 | 0.15 | 0.99 | 0.01 | 0.44 | 0.09 |
| G10U | 0.63 | 0.41 | 0.53 | 0.85 | 0.24 | 0.18 | 0.10 | 0.15 | 0.07 | -- | 0.43 | 0.13 | 0.30 | 0.82 | 0.15 |
| G10X | 0.45 | 0.57 | 0.09 | 0.11 | 0.27 | 0.57 | 0.59 | 0.18 | 0.50 | 0.12 | -- | 0.23 | 0.33 | 0.92 | 0.11 |
| G1A | 0.16 | 0.13 | 0.48 | 0.60 | 0.16 | 0.13 | 0.71 | 0.21 | 0.17 | 0.03 | 0.71 | -- | 0.87 | 0.14 | 0.15 |
| G1D | 0.18 | 0.72 | 0.96 | 0.82 | 0.92 | 0.80 | 0.47 | 0.19 | 0.67 | 0.32 | 0.37 | 0.75 | -- | 0.55 | 0.16 |
| MU50 | 0.32 | 0.18 | 0.93 | 0.68 | 0.24 | 0.72 | 0.09 | 0.23 | 0.49 | 0.45 | 0.64 | 0.00 | 0.09 | -- | 0.51 |
| MU59 | 0.76 | 0.87 | 0.38 | 0.94 | 0.36 | 0.08 | 0.52 | 0.01 | 0.34 | 0.41 | 0.50 | 0.71 | 0.48 | 0.25 | -- |

Table C.4. Estimated linkage disequilibrium for 15 loci used to characterize the genetic structure for regional subpopulations identified by geneland using the uncorrelated allele frequency model for American black bears (*Ursus americanus*) in the American Southwest and northern Mexico. Bolded values indicate a statistically significant test after applying a Bonferroni correction of α < 0.0005. Sky Islands South of Interstate-10 (below diagonal) and Trans-Pecos region (above diagonal).

| Locus | CXX20 | G10B | G10C | G10H | G10J | G10L | G10M | G10O | G10P | G10U | G10X | G1A | G1D | MU50 | MU59 |
| --- | --- | --- | --- | --- | --- | --- | --- | --- | --- | --- | --- | --- | --- | --- | --- |
| CXX20 | -- | 0.07 | 0.81 | 0.92 | 0.37 | 0.16 | 0.25 | 0.48 | 1.00 | 0.51 | 1.00 | 0.74 | 1.00 | 1.00 | 0.34 |
| G10B | 0.14 | -- | 0.11 | 0.75 | 0.55 | 0.60 | 0.30 | 0.82 | 0.78 | 0.72 | 0.32 | 0.39 | 0.62 | 0.25 | 0.09 |
| G10C | 0.84 | 0.39 | -- | 0.08 | 0.94 | 0.14 | 0.84 | 0.78 | 0.93 | 0.27 | 0.58 | 0.82 | 0.09 | 0.16 | 0.45 |
| G10H | 0.30 | 0.91 | 0.41 | -- | 0.90 | 0.01 | 0.96 | 0.70 | 0.13 | 0.93 | 0.91 | 0.51 | 0.13 | 1.00 | 0.98 |
| G10J | 0.57 | 0.40 | 0.57 | 0.18 | -- | 0.84 | 0.19 | 0.04 | 1.00 | 0.30 | 0.60 | 0.44 | 0.67 | 1.00 | 0.04 |
| G10L | 0.19 | 0.11 | 0.45 | 0.76 | 0.25 | -- | 0.97 | 0.07 | 1.00 | 0.05 | 0.45 | 0.71 | 0.10 | 1.00 | 0.23 |
| G10M | 0.83 | 0.15 | 0.31 | 0.85 | 0.04 | 0.78 | -- | 0.87 | 0.40 | 0.15 | 0.76 | 0.11 | 0.25 | 0.10 | 0.11 |
| G10O | 0.10 | 0.52 | 1.00 | 0.77 | 0.85 | 0.04 | 0.67 | -- | 0.30 | 0.01 | 0.27 | 0.30 | 0.78 | 1.00 | 0.19 |
| G10P | 0.05 | 0.27 | 0.79 | 0.68 | 0.75 | 0.41 | 0.57 | 0.59 | -- | 0.51 | 0.07 | 0.81 | 0.01 | 1.00 | 0.19 |
| G10U | 0.83 | 0.53 | 0.10 | 0.80 | 0.94 | 0.77 | 0.88 | 0.69 | 0.13 | -- | 0.15 | 0.16 | 0.05 | 0.73 | 0.00 |
| G10X | 0.84 | 0.31 | 0.15 | 0.10 | 0.01 | 0.00 | 0.36 | 0.99 | 0.78 | 0.34 | -- | 0.93 | 0.06 | 1.00 | 0.23 |
| G1A | 0.05 | 0.70 | 0.17 | 0.09 | 0.04 | 0.21 | 0.08 | 0.96 | 0.23 | 0.66 | 0.02 | -- | 0.32 | 0.28 | 0.04 |
| G1D | 0.07 | 0.42 | 0.68 | 0.80 | 0.15 | 0.43 | 0.89 | 0.42 | 0.29 | 0.68 | 0.54 | 0.39 | -- | 1.00 | 0.22 |
| MU50 | NA | NA | NA | NA | NA | NA | NA | NA | NA | NA | NA | NA | NA | -- | 0.58 |
| MU59 | 0.96 | 0.93 | 0.58 | 0.02 | 0.13 | 0.68 | 0.78 | 0.52 | 0.17 | 0.08 | 0.56 | 0.73 | 0.27 | NA | -- |

Table C.5. Hardy-Weinberg proportion tests for 15 loci used to characterize the genetic structure for regional subpopulations identified by geneland using the uncorrelated allele frequency model American black bears (*Ursus americanus*) in the American Southwest and northern Mexico. Bolded values indicate a statistically significant test after applying a Bonferroni correction of α < 0.003.

| Locus | BM^a^ | ECPSRM^b^ | DMS^c^ | MHSS^d^ | SIS^e^ | TP^f^ |
| --- | --- | --- | --- | --- | --- | --- |
| CXX20 | 1.00 | 0.11 | 0.64 | 0.16 | 0.10 | 0.85 |
| G1A | 0.81 | 0.06 | 0.40 | 0.88 | 0.48 | 0.25 |
| G1D | 0.12 | 0.04 | 0.61 | 0.01 | 0.32 | 0.97 |
| G10B | 0.02 | 0.87 | 0.07 | 0.12 | 0.67 | 0.91 |
| G10C | 0.07 | 0.07 | 0.77 | 0.81 | 1.00 | 0.65 |
| G10H | 0.21 | 0.03 | 0.19 | 0.42 | 0.61 | 0.54 |
| G10J | 0.43 | 0.10 | 0.21 | 0.51 | 0.03 | 0.49 |
| G10L | 0.83 | 0.55 | **< 0.01** | 0.56 | 0.38 | 0.17 |
| G10M | 0.15 | 0.05 | 0.48 | 0.14 | 0.29 | 0.60 |
| G10O | 1.00 | 0.04 | 0.24 | 1.00 | 1.00 | 0.11 |
| G10P | 0.35 | 0.09 | 0.64 | 0.20 | 0.83 | 0.97 |
| G10U | 0.97 | **< 0.01** | 0.80 | 0.21 | 0.86 | 1.00 |
| G10X | 0.29 | 0.14 | 0.90 | 0.39 | 0.65 | 0.41 |
| MU50 | 1.00 | 0.70 | 1.00 | 0.31 | 1.00 | 0.94 |
| MU59 | 0.20 | 0.19 | 0.29 | 0.15 | 0.63 | 0.43 |

^a^ Boulder Mountain (BM), ^b^ eastern Colorado Plateau and Southern Rocky Mountains (ECPSRM), ^c^ Datil-Mogollon Section (DMS), ^d^ Mexican Highland and Sacramento sections (MHSS), ^e^ Sky Islands South of Interstate 10 (SIS), ^f^ Trans-Pecos (TP).

Table C.6. Allele frequencies for the 15 microsatellite loci used to characterize the genetic structure for regional subpopulations identified by geneland using the uncorrelated allele frequency model for American black bears (*Ursus americanus*) in the American Southwest and northern Mexico.

| Locus/allele | BM^a^ | ECPSRM^b^ | DMS^c^ | MHSS^d^ | SIS^e^ | TP^f^ |
| --- | --- | --- | --- | --- | --- | --- |
| CXX20 |  |  |  |  |  |  |
| 123 | 0.000 | 0.004 | 0.032 | 0.000 | 0.000 | 0.150 |
| 129 | 0.000 | 0.006 | 0.000 | 0.008 | 0.000 | 0.100 |
| 131 | 0.000 | 0.512 | 0.817 | 0.554 | 0.536 | 0.000 |
| 133 | 0.119 | 0.198 | 0.042 | 0.200 | 0.009 | 0.000 |
| 137 | 0.000 | 0.103 | 0.025 | 0.000 | 0.000 | 0.025 |
| 139 | 0.833 | 0.117 | 0.060 | 0.115 | 0.136 | 0.350 |
| 141 | 0.000 | 0.002 | 0.000 | 0.000 | 0.000 | 0.275 |
| 143 | 0.000 | 0.055 | 0.011 | 0.123 | 0.300 | 0.100 |
| 145 | 0.000 | 0.000 | 0.014 | 0.000 | 0.000 | 0.000 |
| 147 | 0.048 | 0.002 | 0.000 | 0.000 | 0.018 | 0.000 |
| G1A |  |  |  |  |  |  |
| 184 | 0.190 | 0.000 | 0.000 | 0.000 | 0.000 | 0.000 |
| 188 | 0.000 | 0.040 | 0.042 | 0.215 | 0.000 | 0.000 |
| 192 | 0.524 | 0.721 | 0.835 | 0.585 | 0.682 | 0.000 |
| 194 | 0.286 | 0.142 | 0.092 | 0.169 | 0.045 | 0.800 |
| 196 | 0.000 | 0.030 | 0.000 | 0.000 | 0.000 | 0.000 |
| 198 | 0.000 | 0.055 | 0.000 | 0.008 | 0.000 | 0.175 |
| 200 | 0.000 | 0.000 | 0.007 | 0.000 | 0.009 | 0.025 |
| 202 | 0.000 | 0.012 | 0.000 | 0.023 | 0.073 | 0.000 |
| 204 | 0.000 | 0.000 | 0.025 | 0.000 | 0.191 | 0.000 |
| G1D |  |  |  |  |  |  |
| 172 | 0.000 | 0.174 | 0.176 | 0.169 | 0.309 | 0.000 |
| 174 | 0.190 | 0.045 | 0.007 | 0.000 | 0.000 | 0.000 |
| 176 | 0.357 | 0.180 | 0.137 | 0.223 | 0.055 | 0.200 |
| 178 | 0.000 | 0.002 | 0.018 | 0.000 | 0.245 | 0.000 |
| 180 | 0.119 | 0.156 | 0.049 | 0.200 | 0.000 | 0.000 |
| 182 | 0.000 | 0.000 | 0.000 | 0.000 | 0.000 | 0.050 |
| 184 | 0.333 | 0.383 | 0.514 | 0.392 | 0.318 | 0.425 |
| 186 | 0.000 | 0.061 | 0.099 | 0.015 | 0.073 | 0.300 |
| 188 | 0.000 | 0.000 | 0.000 | 0.000 | 0.000 | 0.025 |
| G10B |  |  |  |  |  |  |
| 154 | 0.000 | 0.000 | 0.000 | 0.000 | 0.000 | 0.025 |
| 156 | 0.000 | 0.014 | 0.021 | 0.223 | 0.009 | 0.675 |
| 158 | 0.119 | 0.071 | 0.018 | 0.008 | 0.018 | 0.000 |
| 160 | 0.405 | 0.022 | 0.000 | 0.000 | 0.018 | 0.125 |
| 162 | 0.095 | 0.320 | 0.183 | 0.308 | 0.373 | 0.150 |
| 164 | 0.333 | 0.447 | 0.630 | 0.262 | 0.509 | 0.025 |
| 166 | 0.048 | 0.119 | 0.148 | 0.200 | 0.073 | 0.000 |
| 168 | 0.000 | 0.004 | 0.000 | 0.000 | 0.000 | 0.000 |
| 170 | 0.000 | 0.002 | 0.000 | 0.000 | 0.000 | 0.000 |
| G10C |  |  |  |  |  |  |
| 197 | 0.000 | 0.099 | 0.070 | 0.031 | 0.036 | 0.000 |
| 199 | 0.524 | 0.798 | 0.806 | 0.815 | 0.955 | 0.000 |
| 203 | 0.476 | 0.065 | 0.102 | 0.154 | 0.009 | 0.000 |
| 209 | 0.000 | 0.000 | 0.000 | 0.000 | 0.000 | 0.150 |
| 211 | 0.000 | 0.036 | 0.021 | 0.000 | 0.000 | 0.125 |
| 213 | 0.000 | 0.000 | 0.000 | 0.000 | 0.000 | 0.100 |
| 215 | 0.000 | 0.002 | 0.000 | 0.000 | 0.000 | 0.625 |
| G10H |  |  |  |  |  |  |
| 231 | 0.000 | 0.020 | 0.000 | 0.046 | 0.000 | 0.000 |
| 237 | 0.143 | 0.431 | 0.694 | 0.554 | 0.827 | 0.125 |
| 239 | 0.571 | 0.213 | 0.130 | 0.015 | 0.018 | 0.100 |
| 241 | 0.024 | 0.136 | 0.000 | 0.023 | 0.000 | 0.750 |
| 243 | 0.000 | 0.028 | 0.014 | 0.023 | 0.100 | 0.000 |
| 245 | 0.119 | 0.075 | 0.137 | 0.200 | 0.045 | 0.000 |
| 249 | 0.000 | 0.000 | 0.000 | 0.008 | 0.000 | 0.000 |
| 251 | 0.000 | 0.000 | 0.000 | 0.000 | 0.000 | 0.025 |
| 252 | 0.071 | 0.020 | 0.000 | 0.000 | 0.000 | 0.000 |
| 265 | 0.000 | 0.069 | 0.004 | 0.115 | 0.009 | 0.000 |
| 267 | 0.071 | 0.008 | 0.021 | 0.015 | 0.000 | 0.000 |
| G10J |  |  |  |  |  |  |
| 185 | 0.000 | 0.010 | 0.000 | 0.023 | 0.000 | 0.225 |
| 187 | 0.381 | 0.053 | 0.025 | 0.062 | 0.000 | 0.325 |
| 189 | 0.000 | 0.010 | 0.014 | 0.000 | 0.009 | 0.150 |
| 195 | 0.000 | 0.170 | 0.271 | 0.108 | 0.718 | 0.000 |
| 197 | 0.381 | 0.123 | 0.088 | 0.115 | 0.091 | 0.000 |
| 199 | 0.024 | 0.000 | 0.000 | 0.000 | 0.018 | 0.000 |
| 201 | 0.000 | 0.000 | 0.000 | 0.008 | 0.000 | 0.000 |
| 203 | 0.119 | 0.437 | 0.468 | 0.208 | 0.155 | 0.225 |
| 205 | 0.095 | 0.148 | 0.095 | 0.046 | 0.009 | 0.075 |
| 207 | 0.000 | 0.049 | 0.039 | 0.431 | 0.000 | 0.000 |
| G10L |  |  |  |  |  |  |
| 135 | 0.000 | 0.006 | 0.000 | 0.008 | 0.009 | 0.150 |
| 137 | 0.071 | 0.144 | 0.155 | 0.069 | 0.091 | 0.000 |
| 139 | 0.000 | 0.004 | 0.025 | 0.000 | 0.091 | 0.575 |
| 141 | 0.048 | 0.251 | 0.144 | 0.023 | 0.100 | 0.000 |
| 145 | 0.048 | 0.202 | 0.092 | 0.262 | 0.073 | 0.000 |
| 149 | 0.000 | 0.008 | 0.000 | 0.000 | 0.000 | 0.100 |
| 157 | 0.000 | 0.000 | 0.000 | 0.000 | 0.000 | 0.050 |
| 159 | 0.738 | 0.213 | 0.289 | 0.331 | 0.318 | 0.125 |
| 161 | 0.000 | 0.022 | 0.032 | 0.008 | 0.000 | 0.000 |
| 167 | 0.048 | 0.014 | 0.074 | 0.085 | 0.000 | 0.000 |
| 169 | 0.048 | 0.136 | 0.190 | 0.215 | 0.318 | 0.000 |
| G10M |  |  |  |  |  |  |
| 206 | 0.143 | 0.000 | 0.021 | 0.000 | 0.009 | 0.000 |
| 210 | 0.310 | 0.138 | 0.102 | 0.062 | 0.191 | 0.275 |
| 211 | 0.000 | 0.002 | 0.000 | 0.000 | 0.000 | 0.000 |
| 212 | 0.357 | 0.374 | 0.345 | 0.369 | 0.755 | 0.325 |
| 213 | 0.095 | 0.298 | 0.331 | 0.292 | 0.018 | 0.000 |
| 214 | 0.071 | 0.178 | 0.201 | 0.177 | 0.027 | 0.025 |
| 216 | 0.024 | 0.006 | 0.000 | 0.100 | 0.000 | 0.375 |
| 217 | 0.000 | 0.004 | 0.000 | 0.000 | 0.000 | 0.000 |
| G10O |  |  |  |  |  |  |
| 150 | 0.000 | 0.000 | 0.000 | 0.000 | 0.000 | 0.100 |
| 154 | 0.024 | 0.057 | 0.007 | 0.000 | 0.036 | 0.000 |
| 156 | 0.000 | 0.002 | 0.000 | 0.000 | 0.000 | 0.000 |
| 180 | 0.000 | 0.000 | 0.000 | 0.000 | 0.000 | 0.050 |
| 192 | 0.000 | 0.000 | 0.000 | 0.000 | 0.000 | 0.475 |
| 194 | 0.000 | 0.032 | 0.000 | 0.031 | 0.000 | 0.000 |
| 196 | 0.571 | 0.812 | 0.926 | 0.915 | 0.936 | 0.000 |
| 198 | 0.405 | 0.071 | 0.000 | 0.023 | 0.000 | 0.050 |
| 204 | 0.000 | 0.000 | 0.000 | 0.000 | 0.000 | 0.025 |
| 208 | 0.000 | 0.024 | 0.000 | 0.031 | 0.000 | 0.125 |
| 210 | 0.000 | 0.002 | 0.067 | 0.000 | 0.027 | 0.175 |
| G10P |  |  |  |  |  |  |
| 147 | 0.000 | 0.010 | 0.000 | 0.000 | 0.000 | 0.200 |
| 151 | 0.000 | 0.000 | 0.000 | 0.000 | 0.000 | 0.175 |
| 153 | 0.571 | 0.761 | 0.694 | 0.700 | 0.600 | 0.000 |
| 155 | 0.000 | 0.028 | 0.011 | 0.000 | 0.000 | 0.000 |
| 157 | 0.000 | 0.040 | 0.011 | 0.008 | 0.000 | 0.075 |
| 159 | 0.262 | 0.091 | 0.074 | 0.269 | 0.091 | 0.250 |
| 161 | 0.167 | 0.024 | 0.158 | 0.000 | 0.055 | 0.000 |
| 163 | 0.000 | 0.018 | 0.046 | 0.008 | 0.164 | 0.300 |
| 165 | 0.000 | 0.012 | 0.007 | 0.015 | 0.091 | 0.000 |
| 167 | 0.000 | 0.014 | 0.000 | 0.000 | 0.000 | 0.000 |
| G10U |  |  |  |  |  |  |
| 161 | 0.000 | 0.063 | 0.011 | 0.000 | 0.009 | 0.000 |
| 163 | 0.048 | 0.034 | 0.095 | 0.000 | 0.045 | 0.000 |
| 165 | 0.167 | 0.403 | 0.306 | 0.223 | 0.118 | 0.000 |
| 167 | 0.357 | 0.243 | 0.370 | 0.154 | 0.736 | 0.000 |
| 169 | 0.000 | 0.057 | 0.049 | 0.231 | 0.091 | 0.000 |
| 171 | 0.000 | 0.000 | 0.007 | 0.000 | 0.000 | 0.000 |
| 173 | 0.000 | 0.032 | 0.000 | 0.069 | 0.000 | 0.000 |
| 175 | 0.000 | 0.006 | 0.109 | 0.054 | 0.000 | 0.300 |
| 177 | 0.429 | 0.154 | 0.053 | 0.262 | 0.000 | 0.625 |
| 179 | 0.000 | 0.008 | 0.000 | 0.008 | 0.000 | 0.000 |
| 181 | 0.000 | 0.000 | 0.000 | 0.000 | 0.000 | 0.075 |
| G10X |  |  |  |  |  |  |
| 161 | 0.000 | 0.063 | 0.011 | 0.000 | 0.009 | 0.000 |
| 163 | 0.048 | 0.034 | 0.095 | 0.000 | 0.045 | 0.000 |
| 165 | 0.167 | 0.403 | 0.306 | 0.223 | 0.118 | 0.000 |
| 167 | 0.357 | 0.243 | 0.370 | 0.154 | 0.736 | 0.000 |
| 169 | 0.000 | 0.057 | 0.049 | 0.231 | 0.091 | 0.000 |
| 171 | 0.000 | 0.000 | 0.007 | 0.000 | 0.000 | 0.000 |
| 173 | 0.000 | 0.032 | 0.000 | 0.069 | 0.000 | 0.000 |
| 175 | 0.000 | 0.006 | 0.109 | 0.054 | 0.000 | 0.300 |
| 177 | 0.429 | 0.154 | 0.053 | 0.262 | 0.000 | 0.625 |
| 179 | 0.000 | 0.008 | 0.000 | 0.008 | 0.000 | 0.000 |
| 181 | 0.000 | 0.000 | 0.000 | 0.000 | 0.000 | 0.075 |
| MU50 |  |  |  |  |  |  |
| 120 | 0.000 | 0.000 | 0.000 | 0.000 | 0.000 | 0.250 |
| 122 | 0.976 | 0.929 | 0.926 | 0.923 | 1.000 | 0.075 |
| 124 | 0.000 | 0.002 | 0.000 | 0.008 | 0.000 | 0.100 |
| 126 | 0.000 | 0.004 | 0.018 | 0.000 | 0.000 | 0.200 |
| 132 | 0.000 | 0.010 | 0.000 | 0.008 | 0.000 | 0.025 |
| 134 | 0.024 | 0.047 | 0.056 | 0.062 | 0.000 | 0.000 |
| 136 | 0.000 | 0.000 | 0.000 | 0.000 | 0.000 | 0.025 |
| 138 | 0.000 | 0.000 | 0.000 | 0.000 | 0.000 | 0.100 |
| 140 | 0.000 | 0.008 | 0.000 | 0.000 | 0.000 | 0.200 |
| 144 | 0.000 | 0.000 | 0.000 | 0.000 | 0.000 | 0.025 |
| MU59 |  |  |  |  |  |  |
| 231 | 0.333 | 0.551 | 0.514 | 0.623 | 0.745 | 0.000 |
| 235 | 0.095 | 0.020 | 0.060 | 0.108 | 0.064 | 0.000 |
| 237 | 0.095 | 0.079 | 0.004 | 0.054 | 0.000 | 0.000 |
| 239 | 0.000 | 0.063 | 0.000 | 0.023 | 0.000 | 0.775 |
| 241 | 0.167 | 0.081 | 0.035 | 0.031 | 0.045 | 0.050 |
| 243 | 0.048 | 0.123 | 0.282 | 0.100 | 0.009 | 0.175 |
| 245 | 0.024 | 0.083 | 0.106 | 0.062 | 0.136 | 0.000 |
| 247 | 0.238 | 0.000 | 0.000 | 0.000 | 0.000 | 0.000 |

^a^ Boulder Mountain (BM), ^b^ eastern Colorado Plateau and Southern Rocky Mountains (ECPSRM), ^c^ Datil-Mogollon Section (DMS), ^d^ Mexican Highland and Sacramento sections (MHSS), ^e^ Sky Islands South of Interstate 10 (SIS), ^f^ Trans-Pecos (TP).

Table C.7. Estimated pairwise-relative migration rates for regional subpopulations identified by geneland using the uncorrelated allele frequency model for American black bears (*Ursus americanus*) in the American Southwest and northern Mexico. Bolded values indicate significant asymmetric migration based on 1,000 bootstrap iterations. Table is interpreted as migration occurring from the cluster in the left-hand column to the cluster in the top row.

| Cluster | BM^a^ | ECPSRM^b^ | DMS^c^ | MHSS^d^ | SIS^e^ | TP^f^ |
| --- | --- | --- | --- | --- | --- | --- |
| BM | -- | 0.14 | 0.08 | 0.10 | 0.05 | 0.02 |
| ECPSRM | 0.13 | -- | **0.49** | 0.66 | 0.16 | **0.02** |
| DMS | 0.07 | **1.00** | -- | **0.51** | 0.22 | 0.01 |
| MHSS | 0.09 | 0.57 | **0.28** | -- | **0.11** | 0.02 |
| SIS | 0.04 | 0.25 | 0.27 | **0.23** | -- | 0.01 |
| TP | 0.01 | **0.05** | 0.02 | 0.03 | 0.02 | -- |

^a^ Boulder Mountain (BM), ^b^ eastern Colorado Plateau and Southern Rocky Mountains (ECPSRM), ^c^ Datil-Mogollon Section (DMS), ^d^ Mexican Highland and Sacramento sections (MHSS), ^e^ Sky Islands South of Interstate 10 (SIS), ^f^ Trans-Pecos (TP).

## Appendix S4. Mountain range subpopulation results


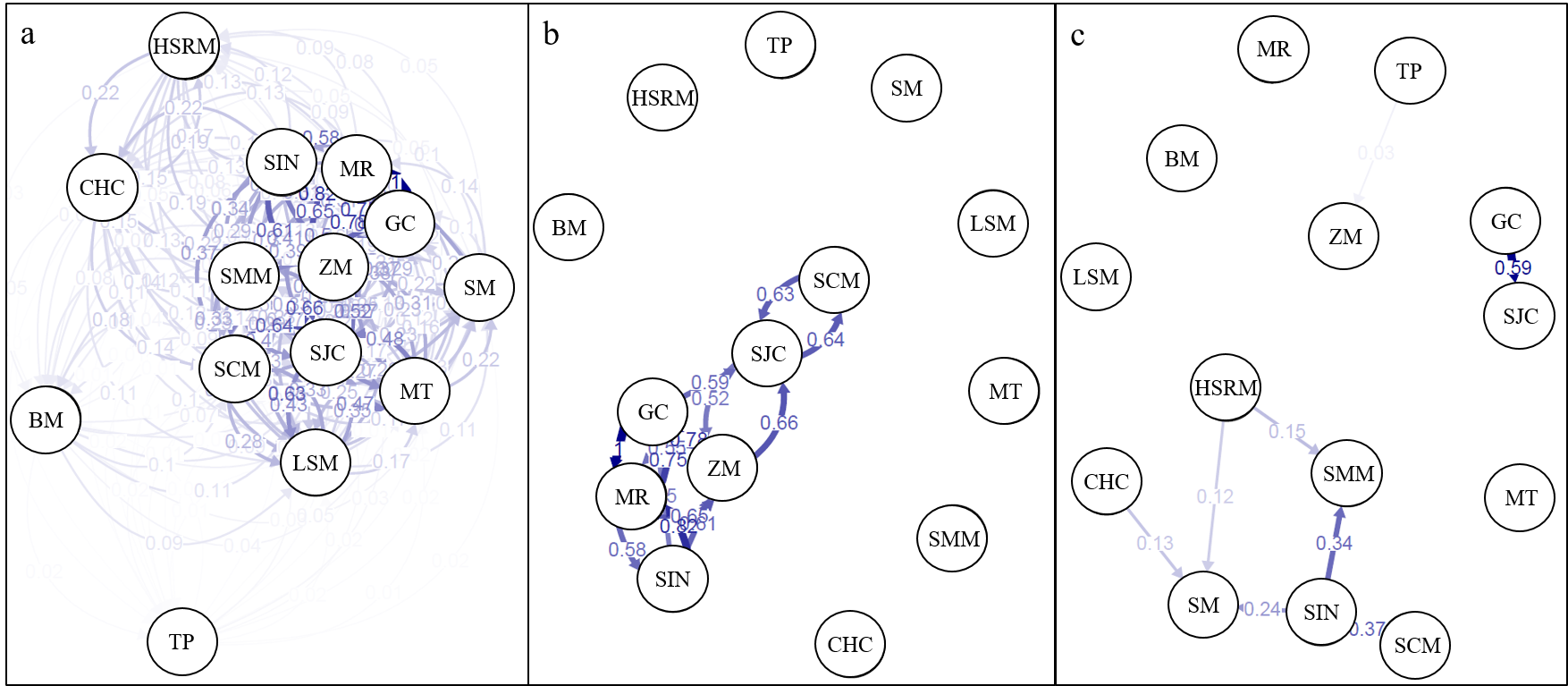


Figure S1. Directional-relative migration networks based on G_ST_ for mountain range subpopulations identified by geneland using the correlated allele frequency model for American black bears (*Ursus americanus*) in the American Southwest and northern Mexico. Networks visualized include (a) all migration values (b) migration values ≥ 0.50, and (c) significant asymmetrical migration values. Populations visualized include Boulder Mountain (BM), La Sal Mountains (LSM), San Juan and Chuska mountains (SJC), Sangre de Cristo Mountains (SCM), Zuni Mountains (ZM), Mt. Taylor (MT), Sandia and Manzano mountains (SMM), Mogollon Rim (MR), Gila complex (GC), Sacramento Mountains (SM), Sky Islands north of Interstate-10 (SIN), Huachuca and Santa Rita mountains (HSRM), Chiricahua complex (CHC), Trans-Pecos region (TP). Significant asymmetrical migration values include GC to SJC (0.59 vs. 0.23), from SIN to SCM (0.37 vs. 0.14), from SIN to SMM (0.34 vs. 0.13), from SIN to SM (0.24 vs. 0.10), from HSRM to SMM (0.15 vs. 0.07), from CHC to SM (0.13 vs. 0.05), from HSRM to SM (0.12 vs. 0.05), and from TP to ZM (0.03 vs. 0.01).

Table S1. Frequency of null alleles and its 95% confidence interval for mountain range subpopulations identified by geneland using the correlated allele frequency model for American black bears (*Ursus americanus*) in the American Southwest and northern Mexico. Bolded values indicate the 95% confidence interval for the allele does not overlap 0.

| Locus | BM^a^ | LSM^b^ | SJC^c^ | SCM^d^ | ZM^e^ | MT^f^ | SMM^g^ |
| --- | --- | --- | --- | --- | --- | --- | --- |
| CXX20 | -**0.07**  **-0.13– -0.03** | -0.05  -0.13–0.03 | **0.09**  **0.01–0.17** | 0.00  -0.07–0.07 | 0.01  -0.12–0.17 | -0.05  -0.16–0.08 | 0.07  -0.07–0.24 |
| G1A | -0.05  -0.17–0.10 | -0.01  -0.12–0.19 | 0.00  -0.06–0.07 | -0.04  -0.09–0.02 | **-0.03**  **-0.06– -0.01** | **-0.09**  **-0.14– -0.05** | 0.00  -0.12–0.14 |
| G1D | 0.07  -0.09–0.25 | 0.06  -0.07–0.20 | **0.07**  **0.01–0.14** | -0.01  -0.06–0.05 | -0.06  -0.12–0.02 | 0.00  -0.11–0.12 | 0.04  -0.06–0.15 |
| G10B | 0.10  -0.05–0.26 | **-0.10**  **-0.16– -0.04** | -0.01  -0.08–0.07 | -0.05  -0.11–0.00 | -0.02  -0.11–0.08 | 0.00  -0.14–0.16 | -0.08  -0.15–0.01 |
| G10C | 0.27  0.00–0.66 | -0.01  -0.08–0.12 | 0.00  -0.08–0.08 | 0.03  -0.05–0.15 | 0.01  -0.10–0.16 | 0.02  -0.12–0.26 | **-0.07**  **-0.11– -0.04** |
| G10H | -0.03  -0.12–0.05 | 0.03  -0.07–0.14 | 0.02  -0.04–0.08 | 0.00  -0.05–0.06 | 0.12  -0.01–0.28 | 0.00  -0.13–0.19 | -0.04  -0.11–0.04 |
| G10J | 0.09  -0.07–0.28 | **0.15**  **0.01–0.29** | 0.01  -0.05–0.08 | -0.02  -0.07–0.04 | -0.01  -0.10–0.09 | -0.06  -0.13–0.01 | -0.03  -0.10–0.05 |
| G10L | -0.04  -0.11–0.05 | 0.10  -0.02–0.25 | -0.01  -0.06–0.04 | 0.00  -0.05–0.05 | -0.05  -0.11–0.02 | 0.02  -0.08–0.15 | **-0.07**  **-0.13– -0.01** |
| G10M | -0.07  -0.17–0.03 | -0.06  -0.15–0.02 | 0.03  -0.04–0.11 | -0.02  -0.08–0.05 | -0.03  -0.12–0.08 | -0.08  -0.17–0.06 | 0.09  -0.02–0.24 |
| G10O | -0.06  -0.21–0.15 | 0.00  -0.11–0.15 | -0.03  -0.07–0.02 | 0.03  -0.05–0.13 | 0.16  -0.05–0.63 | 0.20  -0.06–1.00 | **-0.02**  **-0.05– -0.01** |
| G10P | 0.00  -0.11–0.15 | 0.03  -0.11–0.19 | 0.03  -0.05–0.11 | 0.03  -0.04–0.10 | -0.02  -0.08–0.06 | **-0.04**  **-0.07– -0.02** | -0.04  -0.14–0.10 |
| G10U | -0.01  -0.13–0.13 | 0.03  -0.07–0.14 | -0.02  -0.07–0.04 | -0.06  -0.13–0.01 | 0.00  -0.1–0.11 | 0.03  -0.10–0.17 | -0.01  -0.09–0.07 |
| G10X | 0.09  -0.06–0.26 | 0.07  -0.05–0.21 | -0.02  -0.08–0.04 | -0.03  -0.09–0.03 | -0.05  -0.15–0.08 | -0.03  -0.18–0.16 | 0.01  -0.09–0.13 |
| MU50 | **-0.01**  **-0.05– -0.01** | **-0.11**  **-0.17– -0.06** | 0.00  -0.01–0.00 | **-0.02**  **-0.03– -0.01** | **-0.05**  **-0.08– -0.02** | 0.13  -0.08–0.63 | 0.12  -0.06–0.46 |
| MU59 | -0.04  -0.12–0.04 | 0.02  -0.10–0.23 | -0.03  -0.08–0.03 | -0.01  -0.07–0.05 | 0.01  -0.07–0.11 | 0.00  -0.13–0.15 | 0.04  -0.07–0.16 |

^a^ Boulder Mountain (BM), ^b^ La Sal Mountains (LSM), ^c^ San Juan and Chuska mountains (SJC), ^d^ Sangre de Cristo Mountains (SCM), ^e^ Zuni Mountains (ZM), ^f^ Mt. Taylor (MT), ^g^ Sandia and Manzano mountains (SMM), ^h^ Mogollon Rim (MR), ^i^ Gila complex (GC), ^j^ Sacramento Mountains (SM), ^k^ Sky Islands north of Interstate 10 (SIN), ^l^ Huachuca and Santa Rita mountains (HSRM), ^m^ Chiricahua complex (CHC), ^n^ Trans-Pecos region (TP).

Table S1 continued.

| Locus | MR^h^ | GC^i^ | SM^j^ | SIN^k^ | HSRM^l^ | CHC^m^ | TP^n^ |
| --- | --- | --- | --- | --- | --- | --- | --- |
| CXX20 | 0.02  -0.05–0.08 | 0.01  -0.05–0.11 | 0.03  -0.06–0.13 | **-0.06**  **-0.11– -0.01** | -0.01  -0.12–0.13 | 0.11  -0.10–0.38 | -0.06  -0.14–0.02 |
| G1A | 0.00  -0.07–0.13 | 0.04  -0.10–0.21 | -0.04  -0.17–0.12 | **-0.05**  **-0.09– -0.02** | 0.05  -0.06–0.20 | -0.01  -0.16–0.16 | -0.03  -0.14–0.11 |
| G1D | -0.02  -0.08–0.04 | 0.02  -0.07–0.12 | 0.08  -0.05–0.23 | **-0.06**  **-0.12– -0.01** | 0.01  -0.08–0.11 | **-0.13**  **-0.20– -0.08** | -0.01  -0.15–0.14 |
| G10B | 0.10  -0.01–0.21 | 0.06  -0.06–0.21 | 0.03  -0.07–0.15 | 0.00  -0.12–0.14 | -0.03  -0.13–0.10 | 0.08  -0.10–0.31 | -0.04  -0.13–0.05 |
| G10C | -0.04  -0.1–0.03 | 0.02  -0.05–0.12 | 0.07  -0.11–0.35 | -0.03  -0.09–0.07 | **-0.03**  **-0.05– -0.01** | **-0.02**  **-0.05– -0.02** | 0.01  -0.12–0.17 |
| G10H | -0.03  -0.10–0.04 | 0.00  -0.09–0.14 | 0.06  -0.05–0.21 | 0.04  -0.07–0.16 | **-0.04**  **-0.07– -0.02** | 0.04  -0.13–0.29 | 0.01  -0.12–0.26 |
| G10J | 0.00  -0.07–0.09 | 0.03  -0.08–0.14 | 0.02  -0.09–0.13 | 0.04  -0.07–0.15 | 0.10  -0.05–0.34 | -0.04  -0.18–0.13 | **-0.08**  **-0.15– -0.02** |
| G10L | 0.01  -0.05–0.07 | 0.03  -0.05–0.11 | -0.03  -0.10–0.05 | 0.05  -0.06–0.18 | 0.02  -0.07–0.13 | 0.00  -0.13–0.12 | -0.02  -0.14–0.12 |
| G10M | 0.05  -0.03–0.14 | -0.01  -0.10–0.10 | -0.02  -0.10–0.07 | 0.02  -0.07–0.13 | 0.21  -0.04–0.50 | **-0.11**  **-0.20– -0.03** | 0.02  -0.12–0.19 |
| G10O | 0.08  -0.06–0.29 | **-0.01**  **-0.02– -0.01** | **-0.04**  **-0.08– -0.02** | **-0.02**  **-0.05– -0.01** | **-0.02**  **-0.04– -0.01** | **-0.04**  **-0.09– -0.02** | 0.05  -0.07–0.16 |
| G10P | 0.05  -0.03–0.16 | -0.06  -0.13–0.01 | **-0.14**  **-0.23– -0.04** | 0.06  -0.06–0.24 | -0.07  -0.14–0.00 | **-0.09**  **-0.18– -0.01** | -0.02  -0.12–0.09 |
| G10U | 0.01  -0.06–0.07 | 0.01  -0.08–0.10 | 0.01  -0.08–0.1 | 0.03  -0.07–0.16 | -0.05  -0.11–0.01 | **-0.06**  **-0.12– -0.02** | 0.01  -0.14–0.24 |
| G10X | 0.04  -0.04–0.13 | -0.01  -0.11–0.10 | 0.00  -0.13–0.15 | -0.04  -0.10–0.02 | 0.08  -0.06–0.26 | -0.02  -0.18–0.20 | 0.00  -0.11–0.10 |
| MU50 | **-0.04**  **-0.06– -0.02** | **-0.04**  **-0.06– -0.02** | **-0.02**  **-0.05– -0.01** | **-0.03**  **-0.06– -0.01** | NA | NA | -0.01  -0.09–0.07 |
| MU59 | 0.04  -0.04–0.14 | 0.05  -0.05–0.17 | -0.07  -0.12–0.00 | 0.00  -0.11–0.14 | 0.05  0.08–0.22 | -0.03  -0.13–0.10 | -0.04  -0.14–0.06 |

^a^ Boulder Mountain (BM), ^b^ La Sal Mountains (LSM), ^c^ San Juan and Chuska mountains (SJC), ^d^ Sangre de Cristo Mountains (SCM), ^e^ Zuni Mountains (ZM), ^f^ Mt. Taylor (MT), ^g^ Sandia and Manzano mountains (SMM), ^h^ Mogollon Rim (MR), ^i^ Gila complex (GC), ^j^ Sacramento Mountains (SM), ^k^ Sky Islands north of Interstate 10 (SIN), ^l^ Huachuca and Santa Rita mountains (HSRM), ^m^ Chiricahua complex (CHC), ^n^ Trans-Pecos region (TP).

Table D.2. Estimated linkage disequilibrium for 15 loci used to characterize the genetic structure for mountain range subpopulations identified by geneland using the correlated allele frequency model for American black bears (*Ursus americanus*) in the American Southwest and northern Mexico. Bolded values indicate a statistically significant test after applying a Bonferroni correction of α < 0.0005. Boulder Mountain (below diagonal) and the La Sal Mountains (above diagonal).

| Locus | CXX20 | G10B | G10C | G10H | G10J | G10L | G10M | G10O | G10P | G10U | G10X | G1A | G1D | MU50 | MU59 |
| --- | --- | --- | --- | --- | --- | --- | --- | --- | --- | --- | --- | --- | --- | --- | --- |
| CXX20 | -- | 0.63 | 0.14 | 0.59 | 0.82 | 0.01 | 0.09 | 0.36 | 0.75 | 1.00 | 0.63 | 0.53 | 0.02 | 0.24 | 0.77 |
| G10B | **<0.01** | -- | 0.95 | 0.70 | 0.34 | 0.58 | 0.96 | 0.05 | 0.77 | 0.67 | 0.70 | 0.44 | 0.45 | 0.55 | 0.28 |
| G10C | 0.41 | 0.40 | -- | 0.07 | 0.61 | 0.21 | 0.28 | 0.91 | 0.57 | 0.52 | 0.82 | 0.08 | 0.09 | 0.45 | 0.47 |
| G10H | **<0.01** | 0.62 | 0.96 | -- | 0.02 | 0.32 | 0.82 | 0.76 | 0.84 | 0.54 | 0.97 | 0.20 | 0.83 | 0.38 | 0.77 |
| G10J | 0.01 | 0.85 | 0.75 | 0.02 | -- | 0.44 | 0.28 | 0.80 | 0.69 | 0.43 | 0.07 | 0.34 | 0.27 | 0.46 | 0.05 |
| G10L | 0.20 | 0.84 | 0.54 | 0.15 | 0.26 | -- | 0.88 | 0.02 | 0.44 | 0.78 | 0.52 | 0.21 | 0.41 | 0.90 | 0.99 |
| G10M | 0.10 | 0.62 | 0.79 | 0.06 | 0.10 | 0.39 | -- | 0.43 | 0.19 | 0.15 | 0.69 | 0.06 | 0.39 | 0.16 | 0.08 |
| G10O | 0.04 | 0.95 | 0.13 | 0.04 | 0.52 | 0.58 | 0.23 | -- | 0.58 | 0.91 | 0.42 | 0.28 | 0.08 | 0.38 | 0.28 |
| G10P | 0.30 | 0.20 | 0.17 | 0.23 | 0.15 | 0.47 | 0.32 | 0.27 | -- | 0.86 | 0.05 | 0.46 | 0.12 | 0.97 | 0.83 |
| G10U | 0.86 | 0.19 | 0.99 | 0.05 | 0.98 | 0.35 | 0.49 | 0.77 | 0.60 | -- | 0.37 | 0.89 | 0.18 | 0.32 | 0.10 |
| G10X | 0.89 | 0.94 | 0.45 | 0.97 | 0.89 | 0.28 | 0.97 | 0.73 | 0.54 | 0.95 | -- | 0.30 | 0.11 | 0.30 | 0.64 |
| G1A | 0.32 | 0.42 | 0.59 | 0.45 | 0.26 | 0.32 | 0.12 | 0.13 | 0.19 | 1.00 | 0.22 | -- | 0.11 | 1.00 | 0.16 |
| G1D | 0.45 | 0.63 | 0.43 | 0.52 | 0.82 | 0.09 | 0.05 | 0.04 | 0.69 | 0.90 | 1.00 | 0.16 | -- | 0.44 | 0.68 |
| MU50 | 0.10 | 0.38 | 0.62 | 0.19 | 1.00 | 0.48 | 0.63 | 0.47 | 1.00 | 0.76 | 1.00 | 1.00 | 0.38 | -- | 0.34 |
| MU59 | 0.02 | 0.11 | 0.67 | 0.14 | <0.01 | 0.78 | 0.47 | 0.80 | 0.48 | 0.19 | 0.40 | 0.93 | 0.66 | 1.00 | -- |

Table D.3. Estimated linkage disequilibrium for 15 loci used to characterize the genetic structure for mountain range subpopulations identified by geneland using the correlated allele frequency model for American black bears (*Ursus americanus*) in the American Southwest and northern Mexico. Bolded values indicate a statistically significant test after applying a Bonferroni correction of α < 0.0005. San Juan and Chuska (below diagonal) and Sangre de Cristo (above diagonal) mountains.

| Locus | CXX20 | G10B | G10C | G10H | G10J | G10L | G10M | G10O | G10P | G10U | G10X | G1A | G1D | MU50 | MU59 |
| --- | --- | --- | --- | --- | --- | --- | --- | --- | --- | --- | --- | --- | --- | --- | --- |
| CXX20 | -- | 0.92 | 0.26 | 0.30 | 0.51 | 0.80 | 0.13 | 0.77 | 0.82 | 0.40 | 0.11 | 0.60 | 0.17 | 0.28 | 0.99 |
| G10B | 0.86 | -- | 0.48 | 0.73 | 0.55 | 0.69 | 0.63 | 0.80 | 0.21 | 0.12 | 0.04 | 0.94 | 0.29 | 0.55 | 0.75 |
| G10C | 0.45 | 0.06 | -- | 0.14 | 0.48 | 0.53 | 0.87 | 0.79 | 0.73 | 0.53 | 0.87 | 0.35 | 0.06 | 0.44 | 0.24 |
| G10H | 0.59 | 0.85 | 0.80 | -- | 0.02 | 0.92 | 0.73 | 0.84 | 0.46 | 0.58 | 0.10 | 0.79 | 0.98 | 0.08 | 0.16 |
| G10J | 0.64 | 0.27 | 0.19 | 0.86 | -- | 0.96 | 0.68 | 0.09 | 0.65 | 0.58 | 0.63 | 0.01 | 0.75 | 0.76 | 0.71 |
| G10L | 0.21 | 0.00 | 0.16 | 0.22 | 0.25 | -- | 0.28 | 0.19 | 0.20 | 0.82 | 0.35 | 0.85 | 0.98 | 0.25 | 0.02 |
| G10M | 0.78 | 0.26 | 0.54 | 0.83 | 0.63 | 0.64 | -- | 0.21 | 0.93 | 0.08 | 0.56 | 0.19 | 0.89 | 0.68 | 0.76 |
| G10O | 0.49 | 0.79 | 0.48 | 0.68 | 0.57 | 0.70 | 0.13 | -- | 0.11 | 0.26 | 0.82 | 0.67 | 0.38 | 0.86 | 0.28 |
| G10P | 0.27 | 0.16 | 0.34 | 0.79 | 0.28 | 0.89 | 0.18 | 0.41 | -- | 0.70 | 0.49 | 0.62 | 0.12 | 0.41 | 0.12 |
| G10U | 0.79 | 0.40 | 0.89 | 0.80 | 0.52 | 0.10 | 0.65 | 0.33 | 0.36 | -- | 0.73 | 0.89 | 0.78 | 0.65 | 0.65 |
| G10X | 0.12 | 0.98 | 0.88 | 0.89 | 0.33 | 0.85 | 0.85 | 0.41 | 0.99 | 0.52 | -- | 0.68 | 0.21 | 0.34 | 0.41 |
| G1A | 0.38 | 0.81 | 0.79 | 0.20 | 0.25 | 0.62 | 0.99 | 0.27 | 0.03 | 0.35 | 0.51 | -- | 0.62 | 0.64 | 0.06 |
| G1D | 0.16 | 0.07 | 0.56 | 0.26 | 0.28 | 0.98 | 0.45 | 0.11 | 0.86 | 0.66 | 0.26 | 0.68 | -- | 0.66 | 0.86 |
| MU50 | 0.77 | 0.88 | 0.17 | 0.14 | 0.32 | 0.92 | 0.30 | 0.48 | 1.00 | 0.96 | 0.15 | 1.00 | 0.33 | -- | 0.40 |
| MU59 | 0.07 | 0.05 | 0.49 | 0.43 | 0.93 | 0.88 | 0.74 | 0.32 | 0.67 | 0.62 | 0.30 | 0.49 | 0.18 | 0.61 | -- |

Table D.4. Estimated linkage disequilibrium for 15 loci used to characterize the genetic structure for mountain range subpopulations identified by geneland using the correlated allele frequency model for American black bears (*Ursus americanus*) in the American Southwest and northern Mexico. Bolded values indicate a statistically significant test after applying a Bonferroni correction of α < 0.0005. Zuni Mountains (below diagonal) and Mt. Taylor (above diagonal)

| Locus | CXX20 | G10B | G10C | G10H | G10J | G10L | G10M | G10O | G10P | G10U | G10X | G1A | G1D | MU50 | MU59 |
| --- | --- | --- | --- | --- | --- | --- | --- | --- | --- | --- | --- | --- | --- | --- | --- |
| CXX20 | -- | 0.01 | 0.17 | 1.00 | 0.87 | 0.42 | 0.16 | 0.66 | 0.68 | 0.92 | 0.22 | 0.21 | 0.98 | 0.55 | 0.17 |
| G10B | 0.11 | -- | 0.00 | 0.23 | 0.13 | 0.37 | 0.10 | 0.75 | 0.01 | 0.44 | 0.37 | 0.10 | 0.78 | 0.67 | 0.29 |
| G10C | 0.36 | 0.31 | -- | 0.49 | 0.16 | 0.32 | 0.11 | 0.65 | 0.01 | 0.17 | 0.48 | 0.28 | 0.48 | 0.53 | 0.46 |
| G10H | 0.49 | 0.57 | 0.01 | -- | 0.88 | 0.12 | 0.75 | 0.92 | 0.25 | 0.30 | 0.27 | 0.30 | 0.49 | 0.34 | 0.44 |
| G10J | 0.20 | 0.11 | 0.32 | 0.86 | -- | 0.81 | 0.32 | 1.00 | 0.24 | 0.69 | 0.91 | 0.30 | 0.57 | 0.72 | 0.87 |
| G10L | 0.66 | 0.95 | 0.04 | 0.49 | 0.41 | -- | 0.42 | 0.69 | 0.22 | 0.57 | 0.58 | 0.15 | 0.85 | 0.74 | 0.71 |
| G10M | 0.19 | 0.69 | 0.01 | 0.54 | 0.73 | 0.53 | -- | 0.85 | 0.44 | 0.19 | 0.22 | 0.33 | 0.90 | 1.00 | 0.65 |
| G10O | 0.06 | 0.29 | 0.33 | 0.18 | 0.00 | 0.60 | 0.16 | -- | 0.33 | 0.62 | 0.88 | 0.38 | 0.44 | 0.13 | 0.34 |
| G10P | 0.75 | 0.87 | 0.77 | 0.41 | 0.98 | 0.83 | 0.05 | 1.00 | -- | 0.20 | 0.68 | 0.57 | 0.41 | 0.20 | 0.16 |
| G10U | 0.02 | 0.48 | 0.52 | 0.00 | 0.47 | 0.57 | 0.28 | 0.03 | 0.46 | -- | 0.43 | 0.88 | 0.68 | 0.17 | 0.01 |
| G10X | 0.65 | 0.43 | 0.94 | 0.93 | 0.09 | 0.27 | 0.40 | 0.72 | 0.16 | 0.67 | -- | 0.42 | 0.37 | 0.13 | 0.55 |
| G1A | 0.24 | 0.08 | 0.36 | 0.08 | 0.30 | 0.32 | 0.08 | 0.58 | 0.70 | 0.12 | 0.36 | -- | 0.98 | 0.78 | 0.89 |
| G1D | 0.73 | 0.95 | 0.37 | 0.32 | 0.73 | 0.96 | 0.50 | 0.72 | 0.48 | 0.12 | 0.76 | 0.44 | -- | 0.37 | 0.86 |
| MU50 | 0.10 | 0.00 | 0.07 | 0.76 | 0.23 | 0.45 | 0.41 | 0.24 | 0.94 | 0.59 | 0.72 | 0.22 | 0.57 | -- | 0.14 |
| MU59 | 0.59 | 0.43 | 0.77 | 0.76 | 0.01 | 0.03 | 0.20 | 0.07 | 0.57 | 0.58 | 0.04 | 0.85 | 0.57 | 0.64 | -- |

Table D.5. Estimated linkage disequilibrium for 15 loci used to characterize the genetic structure for mountain range subpopulations identified by geneland using the correlated allele frequency model for American black bears (*Ursus americanus*) in the American Southwest and northern Mexico. Bolded values indicate a statistically significant test after applying a Bonferroni correction of α < 0.0005. Sandia and Manzano mountains (below diagonal) and Mogollon Rim (above diagonal).

| Locus | CXX20 | G10B | G10C | G10H | G10J | G10L | G10M | G10O | G10P | G10U | G10X | G1A | G1D | MU50 | MU59 |
| --- | --- | --- | --- | --- | --- | --- | --- | --- | --- | --- | --- | --- | --- | --- | --- |
| CXX20 | -- | 0.48 | 0.94 | 0.20 | 0.81 | 1.00 | 0.15 | 0.42 | 0.31 | 0.93 | 0.71 | 0.27 | 0.02 | 0.50 | 0.55 |
| G10B | 0.25 | -- | 0.92 | 0.45 | 0.19 | 0.62 | 0.23 | 0.87 | 0.47 | 0.75 | 0.34 | 0.04 | 0.11 | 0.65 | 0.84 |
| G10C | 0.62 | 0.26 | -- | 0.28 | 0.18 | 0.65 | 0.17 | 0.16 | 0.19 | 0.50 | 0.41 | 0.92 | 0.74 | 0.60 | 0.53 |
| G10H | 0.42 | 0.30 | 0.44 | -- | 0.61 | 0.05 | 0.71 | 0.36 | 0.85 | 0.85 | 0.86 | 0.10 | 0.53 | 0.22 | 0.84 |
| G10J | 0.02 | 0.34 | 0.65 | 0.72 | -- | 0.74 | 0.40 | 0.15 | 0.01 | 0.16 | 0.52 | 0.03 | 0.85 | 0.07 | 0.78 |
| G10L | 0.63 | 0.44 | 0.39 | 0.08 | 0.22 | -- | 0.05 | 0.68 | 0.42 | 0.36 | 0.37 | 0.81 | 0.58 | 0.83 | 0.48 |
| G10M | 0.37 | 0.66 | 0.10 | 0.60 | 0.35 | 0.42 | -- | 0.46 | 0.05 | 0.67 | 0.86 | 0.86 | 0.39 | 0.01 | 0.73 |
| G10O | 0.65 | 0.87 | 0.61 | 0.38 | 0.17 | 0.35 | 0.05 | -- | 0.82 | 0.29 | 0.65 | 0.17 | 0.80 | 0.25 | 0.02 |
| G10P | 0.85 | 0.75 | 0.84 | 0.31 | 0.99 | 0.58 | 0.05 | 0.84 | -- | 0.26 | 0.51 | 0.42 | 0.40 | 0.34 | 0.61 |
| G10U | 0.19 | 0.07 | 0.53 | 0.54 | 0.10 | 0.96 | 0.05 | 0.16 | 0.10 | -- | 0.25 | 0.08 | 0.99 | 0.33 | 0.51 |
| G10X | 0.35 | 0.73 | 0.27 | 0.71 | 0.37 | 0.46 | 0.33 | 0.35 | 0.19 | 0.42 | -- | 0.12 | 0.83 | 1.00 | 0.79 |
| G1A | 0.01 | 0.39 | 0.90 | 0.67 | 0.01 | 0.75 | 0.92 | 0.16 | 1.00 | 0.53 | 0.04 | -- | 0.40 | 0.06 | 0.69 |
| G1D | 0.89 | 0.23 | 0.85 | 0.06 | 0.75 | 0.69 | 0.36 | 0.93 | 0.11 | 0.03 | 0.35 | 0.93 | -- | 0.35 | 0.63 |
| MU50 | 0.05 | 0.20 | 0.62 | 0.52 | 0.50 | 0.21 | 0.80 | 0.55 | 0.41 | 0.81 | 0.78 | 0.19 | 0.28 | -- | 0.51 |
| MU59 | 0.96 | 0.77 | 0.35 | 0.02 | 0.22 | 0.63 | 0.16 | 0.45 | 0.16 | 0.28 | 0.58 | 0.78 | 0.33 | 0.29 | -- |

Table D.6. Estimated linkage disequilibrium for 15 loci used to characterize the genetic structure for mountain range subpopulations identified by geneland using the correlated allele frequency model for American black bears (*Ursus americanus*) in the American Southwest and northern Mexico. Bolded values indicate a statistically significant test after applying a Bonferroni correction of α < 0.0005. Gila complex (below diagonal) and Sacramento Mountains (above diagonal)

| Locus | CXX20 | G10B | G10C | G10H | G10J | G10L | G10M | G10O | G10P | G10U | G10X | G1A | G1D | MU50 | MU59 |
| --- | --- | --- | --- | --- | --- | --- | --- | --- | --- | --- | --- | --- | --- | --- | --- |
| CXX20 | -- | 0.56 | 0.45 | 0.14 | 0.96 | 0.37 | 0.98 | 0.01 | 0.43 | 0.99 | 0.18 | 0.46 | 0.10 | 0.10 | 0.01 |
| G10B | 0.90 | -- | 0.56 | 0.37 | 0.41 | 0.77 | 0.86 | 0.06 | 0.83 | 0.28 | 0.82 | 0.36 | 0.38 | 0.97 | 0.41 |
| G10C | 0.36 | 0.14 | -- | 0.13 | 0.95 | 0.89 | 0.43 | 0.88 | 0.36 | 0.80 | 0.53 | 0.37 | 0.84 | 1.00 | 0.60 |
| G10H | 0.51 | 0.41 | 0.28 | -- | 0.68 | 0.85 | 0.48 | 0.50 | 0.02 | 0.22 | 0.86 | 0.65 | 0.12 | 0.62 | 0.94 |
| G10J | 0.50 | 0.69 | 0.23 | 0.59 | -- | 0.41 | 0.99 | 0.67 | 0.30 | 0.99 | 0.80 | 0.44 | 0.34 | 0.53 | 0.88 |
| G10L | 0.96 | 0.23 | 0.64 | 0.58 | 0.10 | -- | 0.55 | 0.13 | 0.28 | 0.86 | 0.73 | 0.45 | 0.89 | 0.69 | 0.92 |
| G10M | 0.31 | 0.64 | 0.98 | 0.01 | 0.81 | 0.94 | -- | 0.30 | 0.85 | 0.09 | 0.05 | 0.55 | 0.81 | 0.19 | 0.68 |
| G10O | 0.23 | 0.48 | 0.02 | 1.00 | 0.55 | 0.73 | 1.00 | -- | 0.07 | 1.00 | 0.01 | 0.98 | 0.39 | 0.18 | 0.26 |
| G10P | 0.97 | 0.33 | 0.73 | 0.95 | 0.33 | 0.60 | 0.73 | 0.52 | -- | 1.00 | 0.12 | 0.79 | 0.26 | 0.75 | 0.23 |
| G10U | 0.24 | 0.57 | 0.67 | 0.76 | 0.92 | 0.33 | 0.57 | 0.20 | 0.23 | -- | 0.95 | 0.51 | 0.86 | 0.77 | 0.94 |
| G10X | 0.55 | 0.52 | 0.55 | 0.44 | 0.69 | 0.78 | 0.63 | 0.25 | 0.75 | 0.07 | -- | 0.84 | 0.95 | 0.30 | 0.06 |
| G1A | 0.31 | 0.41 | 0.66 | 0.82 | 0.78 | 0.00 | 0.17 | 1.00 | 0.70 | 0.10 | 0.44 | -- | 0.91 | 0.44 | 0.65 |
| G1D | 0.43 | 0.41 | 0.67 | 0.99 | 0.49 | 0.42 | 0.64 | 0.45 | 0.89 | 0.26 | 0.13 | 0.68 | -- | 0.18 | 0.06 |
| MU50 | 0.14 | 0.10 | 0.40 | 0.27 | 0.32 | 0.72 | 0.94 | 1.00 | 0.90 | 0.32 | 0.35 | 0.10 | 0.32 | -- | 0.62 |
| MU59 | 0.91 | 0.71 | 0.90 | 0.95 | 0.24 | 0.03 | 0.17 | 0.32 | 0.97 | 0.66 | 0.26 | 0.50 | 0.14 | 0.42 | -- |

Table D.7. Estimated linkage disequilibrium for 15 loci used to characterize the genetic structure for mountain range subpopulations identified by geneland using the correlated allele frequency model for American black bears (*Ursus americanus*) in the American Southwest and northern Mexico. Bolded values indicate a statistically significant test after applying a Bonferroni correction of α < 0.0005. Sky Islands north of Interstate 10 (below diagonal) and Huachuca and Santa Rita mountains (above diagonal)

| Locus | CXX20 | G10B | G10C | G10H | G10J | G10L | G10M | G10O | G10P | G10U | G10X | G1A | G1D | MU50 | MU59 |
| --- | --- | --- | --- | --- | --- | --- | --- | --- | --- | --- | --- | --- | --- | --- | --- |
| CXX20 | -- | 0.17 | 0.86 | 0.03 | 0.97 | 0.26 | 0.99 | 0.16 | 0.54 | 0.44 | 0.62 | 0.78 | 0.23 | NA | 0.63 |
| G10B | 0.93 | -- | 0.75 | 0.69 | 0.98 | 0.07 | 0.90 | 0.08 | 0.38 | 0.74 | 0.73 | 0.95 | 0.59 | NA | 0.94 |
| G10C | 0.36 | 0.02 | -- | 0.53 | 0.75 | 0.40 | 1.00 | 1.00 | 0.77 | 0.41 | 0.26 | 1.00 | 0.70 | NA | 1.00 |
| G10H | 0.91 | 0.69 | 0.11 | -- | 0.46 | 0.94 | 1.00 | 0.16 | 0.46 | 0.27 | 0.33 | 0.11 | 0.91 | NA | 0.06 |
| G10J | 0.26 | 0.23 | 0.87 | 0.84 | -- | 0.93 | 0.13 | 0.67 | 0.81 | 0.95 | 0.20 | 0.16 | 0.58 | NA | 0.70 |
| G10L | 0.14 | 0.24 | 0.28 | 0.38 | 0.47 | -- | 0.68 | 0.15 | 0.35 | 0.86 | 0.03 | 0.12 | 0.45 | NA | 0.98 |
| G10M | 0.07 | 0.38 | 0.24 | 0.90 | 0.02 | 0.85 | -- | 0.72 | 0.61 | 0.82 | 0.27 | 0.08 | 0.82 | NA | 0.86 |
| G10O | 0.39 | 0.44 | 1.00 | 0.52 | 0.39 | 0.24 | 0.08 | -- | 0.64 | 0.63 | 0.47 | 0.53 | 0.27 | NA | 0.92 |
| G10P | 0.30 | 0.73 | 0.61 | 0.90 | 0.44 | 0.80 | 0.99 | 0.96 | -- | 0.19 | 0.85 | 0.45 | 0.08 | NA | 0.01 |
| G10U | 0.36 | 0.86 | 0.59 | 0.59 | 0.07 | 0.77 | 0.46 | 0.04 | 0.59 | -- | 0.28 | 0.18 | 0.54 | NA | 0.06 |
| G10X | 0.97 | 0.67 | 0.36 | 0.17 | 0.45 | 0.93 | 0.81 | 0.15 | 0.07 | 0.42 | -- | 0.01 | 0.29 | NA | 0.39 |
| G1A | 0.58 | 0.88 | 0.13 | 0.72 | 0.11 | 0.36 | 0.82 | 0.67 | 0.04 | 0.18 | 0.54 | -- | 0.64 | NA | 0.52 |
| G1D | 0.06 | 0.77 | 0.59 | 0.97 | 0.98 | 0.57 | 0.62 | 0.01 | 0.46 | 0.17 | 0.25 | 0.74 | -- | NA | 0.26 |
| MU50 | 0.25 | 0.07 | 0.07 | 0.23 | 0.48 | 0.89 | 0.57 | 1.00 | 0.53 | 0.63 | 0.48 | 1.00 | 0.69 | -- | NA |
| MU59 | 0.22 | 0.53 | 0.51 | 0.39 | 0.15 | 0.33 | 0.20 | 0.11 | 0.05 | 0.63 | 0.68 | 0.68 | 0.87 | 0.91 | -- |

Table D.8. Estimated linkage disequilibrium for 15 loci used to characterize the genetic structure for mountain range subpopulations identified by geneland using the correlated allele frequency model for American black bears (*Ursus americanus*) in the American Southwest and northern Mexico. Bolded values indicate a statistically significant test after applying a Bonferroni correction of α < 0.0005. Chiricahua complex (below diagonal) and Trans-Pecos region (above diagonal).

| Locus | CXX20 | G10B | G10C | G10H | G10J | G10L | G10M | G10O | G10P | G10U | G10X | G1A | G1D | MU50 | MU59 |
| --- | --- | --- | --- | --- | --- | --- | --- | --- | --- | --- | --- | --- | --- | --- | --- |
| CXX20 | -- | 0.07 | 0.83 | 0.92 | 0.39 | 0.16 | 0.27 | 0.46 | 1.00 | 0.50 | 1.00 | 0.74 | 1.00 | 1.00 | 0.35 |
| G10B | 0.84 | -- | 0.11 | 0.74 | 0.56 | 0.61 | 0.30 | 0.80 | 0.77 | 0.72 | 0.33 | 0.40 | 0.65 | 0.26 | 0.09 |
| G10C | 1.00 | 0.31 | -- | 0.07 | 0.94 | 0.13 | 0.83 | 0.78 | 0.93 | 0.26 | 0.57 | 0.83 | 0.09 | 0.15 | 0.45 |
| G10H | 0.97 | 0.88 | 0.49 | -- | 0.90 | 0.01 | 0.96 | 0.71 | 0.11 | 0.93 | 0.90 | 0.51 | 0.13 | 1.00 | 0.98 |
| G10J | 0.68 | 0.87 | 1.00 | 0.81 | -- | 0.84 | 0.22 | 0.04 | 1.00 | 0.29 | 0.60 | 0.42 | 0.65 | 1.00 | 0.04 |
| G10L | 0.99 | 0.91 | 0.75 | 0.20 | 0.04 | -- | 0.97 | 0.08 | 1.00 | 0.05 | 0.46 | 0.71 | 0.10 | 1.00 | 0.24 |
| G10M | 0.93 | 0.07 | 0.19 | 0.24 | 0.20 | 0.22 | -- | 0.87 | 0.40 | 0.16 | 0.74 | 0.11 | 0.26 | 0.09 | 0.11 |
| G10O | 0.56 | 0.90 | 1.00 | 1.00 | 0.50 | 0.20 | 0.84 | -- | 0.32 | 0.01 | 0.25 | 0.30 | 0.77 | 1.00 | 0.18 |
| G10P | 0.04 | 0.99 | 0.63 | 0.75 | 0.95 | 0.99 | 1.00 | 1.00 | -- | 0.52 | 0.07 | 0.82 | 0.01 | 1.00 | 0.19 |
| G10U | 1.00 | 0.02 | 0.13 | 0.92 | 0.94 | 0.57 | 0.24 | 0.80 | 0.75 | -- | 0.16 | 0.15 | 0.06 | 0.74 | 0.00 |
| G10X | 0.99 | 0.22 | 0.06 | 0.12 | 0.32 | 0.02 | 0.43 | 0.84 | 0.57 | 0.05 | -- | 0.93 | 0.06 | 1.00 | 0.22 |
| G1A | 0.51 | 0.82 | 0.19 | 0.62 | 0.20 | 0.85 | 0.69 | 0.76 | 0.14 | 0.54 | 0.25 | -- | 0.32 | 0.28 | 0.04 |
| G1D | 0.14 | 0.77 | 1.00 | 0.03 | 0.32 | 0.61 | 0.82 | 0.62 | 0.57 | 1.00 | 0.51 | 0.98 | -- | 1.00 | 0.22 |
| MU50 | NA | NA | NA | NA | NA | NA | NA | NA | NA | NA | NA | NA | NA | -- | 0.57 |
| MU59 | 0.80 | 0.80 | 0.25 | 0.10 | 0.26 | 0.42 | 0.17 | 0.45 | 0.99 | 0.55 | 0.11 | 0.42 | 0.99 | NA | -- |

| Locus | BM^a^ | LSM^b^ | SJC^c^ | SCM^d^ | ZM^e^ | MT^f^ | SMM^g^ | MR^h^ | GC^i^ | SM^j^ | SIN^k^ | HSRM^l^ | CHC^m^ | TP^n^ |
| --- | --- | --- | --- | --- | --- | --- | --- | --- | --- | --- | --- | --- | --- | --- |
| CXX20 | 1.00 | 1.00 | 0.03 | 0.53 | 0.25 | 0.38 | 0.08 | 0.12 | 0.43 | 0.24 | 0.95 | 0.95 | 0.21 | 0.87 |
| G1A | 0.84 | 1.00 | 0.21 | 0.38 | 1.00 | 1.00 | 0.80 | 0.76 | 0.72 | 0.18 | 1.00 | 0.25 | 0.49 | 0.26 |
| G1D | 0.14 | 0.56 | 0.08 | 0.24 | 0.90 | 0.62 | 0.06 | 0.25 | 0.90 | 0.32 | 0.67 | 0.48 | 0.39 | 0.97 |
| G10B | 0.02 | 0.41 | 0.99 | 0.95 | 0.81 | 0.44 | 0.69 | 0.10 | 0.60 | 0.41 | 0.25 | 0.60 | 0.42 | 0.89 |
| G10C | 0.06 | 0.51 | 0.95 | 0.34 | 0.12 | 0.46 | 1.00 | 0.87 | 0.37 | 0.58 | 0.68 | 1.00 | 1.00 | 0.65 |
| G10H | 0.20 | 0.14 | 0.72 | 0.66 | 0.05 | 0.29 | 0.48 | 0.65 | 0.83 | 0.27 | 0.32 | 1.00 | 0.63 | 0.54 |
| G10J | 0.43 | 0.01 | 0.13 | 0.91 | 0.83 | 0.35 | 0.63 | 0.25 | 0.08 | 0.65 | 0.26 | 0.06 | 0.53 | 0.50 |
| G10L | 0.83 | 0.03 | 0.74 | 0.68 | 0.36 | 0.12 | 0.60 | 0.14 | 0.65 | 0.82 | 0.38 | 0.38 | 0.83 | 0.18 |
| G10M | 0.16 | 0.28 | 0.05 | 0.14 | 0.62 | 1.00 | 0.09 | 0.86 | 0.43 | 0.26 | 0.91 | 0.02 | 0.82 | 0.61 |
| G10O | 1.00 | 0.57 | 0.86 | 0.45 | 0.11 | 0.20 | 1.00 | 0.60 | 1.00 | 1.00 | 1.00 | 1.00 | 1.00 | 0.10 |
| G10P | 0.34 | 0.78 | 0.47 | 0.33 | 0.73 | 1.00 | 0.45 | 0.65 | 0.30 | 0.12 | 0.48 | 0.91 | 0.67 | 0.96 |
| G10U | 0.98 | 0.16 | 0.85 | 0.11 | 0.19 | 0.01 | 0.41 | 0.36 | 0.44 | 0.82 | 0.62 | 0.87 | 1.00 | 1.00 |
| G10X | 0.26 | 0.19 | 0.80 | 0.04 | 0.04 | 0.21 | 0.10 | 0.78 | 0.69 | 0.25 | 0.96 | 0.35 | 0.88 | 0.43 |
| MU50 | 1.00 | 0.56 | 1.00 | 1.00 | 1.00 | 0.31 | 0.31 | 0.54 | 1.00 | 1.00 | 1.00 | 1.00 | 1.00 | 0.94 |
| MU59 | 0.21 | 0.72 | 0.92 | 0.91 | 0.12 | 0.02 | 0.17 | 0.50 | 0.37 | 1.00 | 0.78 | 0.24 | 0.53 | 0.41 |

Table D.9. Hardy-Weinberg proportion tests for 15 loci used to characterize the genetic structure for mountain range subpopulations identified by GENELAND using the correlated allele frequency model American black bears (*Ursus americanus*) in the American Southwest and northern Mexico. Bolded values indicate a statistically significant test after applying a Bonferroni correction of α < 0.003.

^a^ Boulder Mountain (BM), ^b^ La Sal Mountains (LSM), ^c^ San Juan and Chuska mountains (SJC), ^d^ Sangre de Cristo Mountains (SCM), ^e^ Zuni Mountains (ZM), ^f^ Mt. Taylor (MT), ^g^ Sandia and Manzano mountains (SMM), ^h^ Mogollon Rim (MR), ^i^ Gila complex (GC), ^j^ Sacramento Mountains (SM), ^k^ Sky Islands north of Interstate 10 (SIN), ^l^ Huachuca and Santa Rita mountains (HSRM), ^m^ Chiricahua complex (CHC), ^n^ Trans-Pecos region (TP).

Table D.10. Allele frequencies for the 15 microsatellite loci used to characterize the genetic structure for mountain range subpopulations identified by GENELAND using the correlated allele frequency model for American black bears (*Ursus americanus*) in the American Southwest and northern Mexico.

| Locus/allele | BM^a^ | LSM^b^ | SJC^c^ | SCM^d^ | ZM^e^ | MT^f^ | SMM^g^ | MR^h^ | GC^i^ | SM^j^ | SIN^k^ | HSRM^l^ | CHC^m^ | TP^n^ |
| --- | --- | --- | --- | --- | --- | --- | --- | --- | --- | --- | --- | --- | --- | --- |
| CXX20 |  |  |  |  |  |  |  |  |  |  |  |  |  |  |
| 123 | 0.000 | 0.000 | 0.000 | 0.000 | 0.030 | 0.000 | 0.000 | 0.008 | 0.045 | 0.000 | 0.057 | 0.000 | 0.000 | 0.150 |
| 129 | 0.000 | 0.000 | 0.000 | 0.012 | 0.015 | 0.000 | 0.015 | 0.000 | 0.000 | 0.000 | 0.000 | 0.000 | 0.000 | 0.100 |
| 131 | 0.000 | 0.321 | 0.390 | 0.605 | 0.697 | 0.587 | 0.632 | 0.825 | 0.875 | 0.468 | 0.743 | 0.487 | 0.625 | 0.000 |
| 133 | 0.119 | 0.250 | 0.226 | 0.198 | 0.076 | 0.217 | 0.191 | 0.056 | 0.011 | 0.210 | 0.057 | 0.000 | 0.031 | 0.000 |
| 137 | 0.000 | 0.143 | 0.122 | 0.111 | 0.015 | 0.087 | 0.000 | 0.048 | 0.000 | 0.000 | 0.014 | 0.000 | 0.000 | 0.025 |
| 139 | 0.833 | 0.268 | 0.189 | 0.031 | 0.045 | 0.087 | 0.029 | 0.048 | 0.034 | 0.210 | 0.100 | 0.103 | 0.250 | 0.350 |
| 141 | 0.000 | 0.000 | 0.000 | 0.006 | 0.000 | 0.000 | 0.000 | 0.000 | 0.000 | 0.000 | 0.000 | 0.000 | 0.000 | 0.275 |
| 143 | 0.000 | 0.018 | 0.067 | 0.037 | 0.121 | 0.022 | 0.132 | 0.000 | 0.023 | 0.113 | 0.014 | 0.410 | 0.031 | 0.100 |
| 145 | 0.000 | 0.000 | 0.000 | 0.000 | 0.000 | 0.000 | 0.000 | 0.016 | 0.011 | 0.000 | 0.014 | 0.000 | 0.000 | 0.000 |
| 147 | 0.048 | 0.000 | 0.006 | 0.000 | 0.000 | 0.000 | 0.000 | 0.000 | 0.000 | 0.000 | 0.000 | 0.000 | 0.062 | 0.000 |
| G1A |  |  |  |  |  |  |  |  |  |  |  |  |  |  |
| 184 | 0.190 | 0.000 | 0.000 | 0.000 | 0.000 | 0.000 | 0.000 | 0.000 | 0.000 | 0.000 | 0.000 | 0.000 | 0.000 | 0.000 |
| 188 | 0.000 | 0.036 | 0.055 | 0.025 | 0.045 | 0.043 | 0.103 | 0.095 | 0.000 | 0.339 | 0.000 | 0.000 | 0.000 | 0.000 |
| 192 | 0.524 | 0.786 | 0.689 | 0.636 | 0.924 | 0.761 | 0.603 | 0.857 | 0.750 | 0.565 | 0.900 | 0.782 | 0.438 | 0.000 |
| 194 | 0.286 | 0.179 | 0.122 | 0.198 | 0.030 | 0.130 | 0.265 | 0.048 | 0.216 | 0.065 | 0.000 | 0.026 | 0.125 | 0.800 |
| 196 | 0.000 | 0.000 | 0.024 | 0.068 | 0.000 | 0.000 | 0.000 | 0.000 | 0.000 | 0.000 | 0.000 | 0.000 | 0.000 | 0.000 |
| 198 | 0.000 | 0.000 | 0.079 | 0.068 | 0.000 | 0.065 | 0.015 | 0.000 | 0.000 | 0.000 | 0.000 | 0.000 | 0.000 | 0.175 |
| 200 | 0.000 | 0.000 | 0.000 | 0.000 | 0.000 | 0.000 | 0.000 | 0.000 | 0.023 | 0.000 | 0.000 | 0.000 | 0.031 | 0.025 |
| 202 | 0.000 | 0.000 | 0.030 | 0.006 | 0.000 | 0.000 | 0.015 | 0.000 | 0.000 | 0.032 | 0.000 | 0.077 | 0.062 | 0.000 |
| 204 | 0.000 | 0.000 | 0.000 | 0.000 | 0.000 | 0.000 | 0.000 | 0.000 | 0.011 | 0.000 | 0.100 | 0.115 | 0.344 | 0.000 |
| G1D |  |  |  |  |  |  |  |  |  |  |  |  |  |  |
| 172 | 0.000 | 0.393 | 0.104 | 0.198 | 0.152 | 0.109 | 0.088 | 0.190 | 0.182 | 0.258 | 0.143 | 0.308 | 0.312 | 0.000 |
| 174 | 0.190 | 0.036 | 0.073 | 0.031 | 0.030 | 0.022 | 0.000 | 0.000 | 0.011 | 0.000 | 0.014 | 0.000 | 0.000 | 0.000 |
| 176 | 0.357 | 0.161 | 0.207 | 0.117 | 0.197 | 0.304 | 0.279 | 0.095 | 0.261 | 0.161 | 0.057 | 0.051 | 0.062 | 0.200 |
| 178 | 0.000 | 0.000 | 0.000 | 0.000 | 0.015 | 0.000 | 0.000 | 0.016 | 0.000 | 0.000 | 0.043 | 0.321 | 0.062 | 0.000 |
| 180 | 0.119 | 0.125 | 0.165 | 0.154 | 0.106 | 0.239 | 0.279 | 0.056 | 0.057 | 0.113 | 0.029 | 0.000 | 0.000 | 0.000 |
| 182 | 0.000 | 0.000 | 0.000 | 0.000 | 0.000 | 0.000 | 0.000 | 0.000 | 0.000 | 0.000 | 0.000 | 0.000 | 0.000 | 0.050 |
| 184 | 0.333 | 0.286 | 0.409 | 0.377 | 0.470 | 0.304 | 0.324 | 0.548 | 0.432 | 0.468 | 0.557 | 0.295 | 0.375 | 0.425 |
| 186 | 0.000 | 0.000 | 0.043 | 0.123 | 0.030 | 0.022 | 0.029 | 0.095 | 0.057 | 0.000 | 0.157 | 0.026 | 0.188 | 0.300 |
| 188 | 0.000 | 0.000 | 0.000 | 0.000 | 0.000 | 0.000 | 0.000 | 0.000 | 0.000 | 0.000 | 0.000 | 0.000 | 0.000 | 0.025 |
| G10B |  |  |  |  |  |  |  |  |  |  |  |  |  |  |
| 154 | 0.000 | 0.000 | 0.000 | 0.000 | 0.000 | 0.000 | 0.000 | 0.000 | 0.000 | 0.000 | 0.000 | 0.000 | 0.000 | 0.025 |
| 156 | 0.000 | 0.018 | 0.006 | 0.025 | 0.015 | 0.000 | 0.059 | 0.032 | 0.011 | 0.403 | 0.029 | 0.000 | 0.000 | 0.675 |
| 158 | 0.119 | 0.214 | 0.043 | 0.080 | 0.030 | 0.022 | 0.000 | 0.040 | 0.000 | 0.016 | 0.000 | 0.000 | 0.062 | 0.000 |
| 160 | 0.405 | 0.000 | 0.024 | 0.031 | 0.030 | 0.000 | 0.000 | 0.000 | 0.000 | 0.000 | 0.000 | 0.026 | 0.000 | 0.125 |
| 162 | 0.095 | 0.125 | 0.305 | 0.444 | 0.227 | 0.304 | 0.426 | 0.198 | 0.125 | 0.177 | 0.214 | 0.449 | 0.219 | 0.150 |
| 164 | 0.333 | 0.536 | 0.549 | 0.272 | 0.530 | 0.478 | 0.265 | 0.603 | 0.705 | 0.258 | 0.600 | 0.436 | 0.656 | 0.025 |
| 166 | 0.048 | 0.107 | 0.073 | 0.142 | 0.152 | 0.174 | 0.250 | 0.127 | 0.159 | 0.145 | 0.157 | 0.090 | 0.062 | 0.000 |
| 168 | 0.000 | 0.000 | 0.000 | 0.006 | 0.015 | 0.000 | 0.000 | 0.000 | 0.000 | 0.000 | 0.000 | 0.000 | 0.000 | 0.000 |
| 170 | 0.000 | 0.000 | 0.000 | 0.000 | 0.000 | 0.022 | 0.000 | 0.000 | 0.000 | 0.000 | 0.000 | 0.000 | 0.000 | 0.000 |
| G10C |  |  |  |  |  |  |  |  |  |  |  |  |  |  |
| 197 | 0.000 | 0.089 | 0.140 | 0.093 | 0.015 | 0.109 | 0.059 | 0.087 | 0.045 | 0.000 | 0.071 | 0.051 | 0.000 | 0.000 |
| 199 | 0.524 | 0.839 | 0.738 | 0.877 | 0.727 | 0.783 | 0.838 | 0.730 | 0.898 | 0.790 | 0.829 | 0.949 | 0.969 | 0.000 |
| 203 | 0.476 | 0.071 | 0.110 | 0.031 | 0.076 | 0.000 | 0.103 | 0.135 | 0.057 | 0.210 | 0.100 | 0.000 | 0.031 | 0.000 |
| 209 | 0.000 | 0.000 | 0.000 | 0.000 | 0.000 | 0.000 | 0.000 | 0.000 | 0.000 | 0.000 | 0.000 | 0.000 | 0.000 | 0.150 |
| 211 | 0.000 | 0.000 | 0.012 | 0.000 | 0.167 | 0.109 | 0.000 | 0.048 | 0.000 | 0.000 | 0.000 | 0.000 | 0.000 | 0.125 |
| 213 | 0.000 | 0.000 | 0.000 | 0.000 | 0.000 | 0.000 | 0.000 | 0.000 | 0.000 | 0.000 | 0.000 | 0.000 | 0.000 | 0.100 |
| 215 | 0.000 | 0.000 | 0.000 | 0.000 | 0.015 | 0.000 | 0.000 | 0.000 | 0.000 | 0.000 | 0.000 | 0.000 | 0.000 | 0.625 |
| G10H |  |  |  |  |  |  |  |  |  |  |  |  |  |  |
| 231 | 0.000 | 0.071 | 0.024 | 0.000 | 0.030 | 0.000 | 0.044 | 0.000 | 0.000 | 0.048 | 0.000 | 0.000 | 0.000 | 0.000 |
| 237 | 0.143 | 0.411 | 0.530 | 0.259 | 0.500 | 0.609 | 0.515 | 0.706 | 0.795 | 0.597 | 0.557 | 0.872 | 0.688 | 0.125 |
| 239 | 0.571 | 0.196 | 0.134 | 0.389 | 0.121 | 0.022 | 0.029 | 0.159 | 0.136 | 0.000 | 0.071 | 0.026 | 0.000 | 0.100 |
| 241 | 0.024 | 0.143 | 0.140 | 0.099 | 0.152 | 0.217 | 0.029 | 0.000 | 0.000 | 0.016 | 0.000 | 0.000 | 0.000 | 0.750 |
| 243 | 0.000 | 0.000 | 0.018 | 0.068 | 0.000 | 0.000 | 0.029 | 0.000 | 0.000 | 0.016 | 0.057 | 0.051 | 0.219 | 0.000 |
| 245 | 0.119 | 0.036 | 0.079 | 0.062 | 0.121 | 0.087 | 0.191 | 0.119 | 0.068 | 0.210 | 0.243 | 0.038 | 0.094 | 0.000 |
| 249 | 0.000 | 0.000 | 0.000 | 0.000 | 0.000 | 0.000 | 0.015 | 0.000 | 0.000 | 0.000 | 0.000 | 0.000 | 0.000 | 0.000 |
| 251 | 0.000 | 0.000 | 0.000 | 0.000 | 0.000 | 0.000 | 0.000 | 0.000 | 0.000 | 0.000 | 0.000 | 0.000 | 0.000 | 0.025 |
| 252 | 0.071 | 0.054 | 0.030 | 0.006 | 0.015 | 0.000 | 0.000 | 0.000 | 0.000 | 0.000 | 0.000 | 0.000 | 0.000 | 0.000 |
| 265 | 0.000 | 0.089 | 0.043 | 0.093 | 0.061 | 0.065 | 0.118 | 0.008 | 0.000 | 0.113 | 0.000 | 0.013 | 0.000 | 0.000 |
| 267 | 0.071 | 0.000 | 0.000 | 0.025 | 0.000 | 0.000 | 0.029 | 0.008 | 0.000 | 0.000 | 0.071 | 0.000 | 0.000 | 0.000 |
| G10J |  |  |  |  |  |  |  |  |  |  |  |  |  |  |
| 185 | 0.000 | 0.000 | 0.000 | 0.031 | 0.000 | 0.000 | 0.000 | 0.000 | 0.000 | 0.048 | 0.000 | 0.000 | 0.000 | 0.225 |
| 187 | 0.381 | 0.018 | 0.043 | 0.080 | 0.076 | 0.000 | 0.118 | 0.040 | 0.000 | 0.000 | 0.029 | 0.000 | 0.000 | 0.325 |
| 189 | 0.000 | 0.018 | 0.006 | 0.000 | 0.030 | 0.022 | 0.000 | 0.008 | 0.000 | 0.000 | 0.043 | 0.000 | 0.031 | 0.150 |
| 195 | 0.000 | 0.321 | 0.122 | 0.130 | 0.227 | 0.217 | 0.176 | 0.270 | 0.170 | 0.032 | 0.386 | 0.859 | 0.406 | 0.000 |
| 197 | 0.381 | 0.179 | 0.152 | 0.117 | 0.045 | 0.087 | 0.074 | 0.087 | 0.091 | 0.161 | 0.086 | 0.090 | 0.094 | 0.000 |
| 199 | 0.024 | 0.000 | 0.000 | 0.000 | 0.000 | 0.000 | 0.000 | 0.000 | 0.000 | 0.000 | 0.000 | 0.026 | 0.000 | 0.000 |
| 201 | 0.000 | 0.000 | 0.000 | 0.000 | 0.000 | 0.000 | 0.000 | 0.000 | 0.000 | 0.016 | 0.000 | 0.000 | 0.000 | 0.000 |
| 203 | 0.119 | 0.375 | 0.488 | 0.420 | 0.500 | 0.304 | 0.221 | 0.484 | 0.545 | 0.194 | 0.357 | 0.026 | 0.438 | 0.225 |
| 205 | 0.095 | 0.018 | 0.122 | 0.210 | 0.091 | 0.261 | 0.059 | 0.103 | 0.102 | 0.032 | 0.071 | 0.000 | 0.031 | 0.075 |
| 207 | 0.000 | 0.071 | 0.067 | 0.012 | 0.030 | 0.109 | 0.353 | 0.008 | 0.091 | 0.516 | 0.029 | 0.000 | 0.000 | 0.000 |
| G10L |  |  |  |  |  |  |  |  |  |  |  |  |  |  |
| 135 | 0.000 | 0.000 | 0.000 | 0.019 | 0.000 | 0.000 | 0.015 | 0.000 | 0.000 | 0.000 | 0.000 | 0.013 | 0.000 | 0.150 |
| 137 | 0.071 | 0.107 | 0.159 | 0.117 | 0.212 | 0.130 | 0.059 | 0.183 | 0.182 | 0.081 | 0.043 | 0.026 | 0.312 | 0.000 |
| 139 | 0.000 | 0.000 | 0.000 | 0.000 | 0.030 | 0.000 | 0.000 | 0.048 | 0.011 | 0.000 | 0.000 | 0.077 | 0.125 | 0.575 |
| 141 | 0.048 | 0.500 | 0.262 | 0.204 | 0.242 | 0.087 | 0.044 | 0.095 | 0.193 | 0.000 | 0.171 | 0.128 | 0.031 | 0.000 |
| 145 | 0.048 | 0.071 | 0.280 | 0.185 | 0.182 | 0.174 | 0.309 | 0.111 | 0.114 | 0.210 | 0.029 | 0.103 | 0.000 | 0.000 |
| 149 | 0.000 | 0.018 | 0.018 | 0.000 | 0.000 | 0.000 | 0.000 | 0.000 | 0.000 | 0.000 | 0.000 | 0.000 | 0.000 | 0.100 |
| 157 | 0.000 | 0.000 | 0.000 | 0.000 | 0.000 | 0.000 | 0.000 | 0.000 | 0.000 | 0.000 | 0.000 | 0.000 | 0.000 | 0.050 |
| 159 | 0.738 | 0.143 | 0.140 | 0.253 | 0.227 | 0.391 | 0.309 | 0.294 | 0.227 | 0.355 | 0.386 | 0.385 | 0.094 | 0.125 |
| 161 | 0.000 | 0.054 | 0.000 | 0.019 | 0.000 | 0.109 | 0.000 | 0.040 | 0.011 | 0.016 | 0.043 | 0.000 | 0.000 | 0.000 |
| 167 | 0.048 | 0.036 | 0.012 | 0.000 | 0.015 | 0.043 | 0.015 | 0.111 | 0.057 | 0.161 | 0.029 | 0.000 | 0.000 | 0.000 |
| 169 | 0.048 | 0.071 | 0.128 | 0.204 | 0.091 | 0.065 | 0.250 | 0.119 | 0.205 | 0.177 | 0.300 | 0.269 | 0.438 | 0.000 |
| G10M |  |  |  |  |  |  |  |  |  |  |  |  |  |  |
| 206 | 0.143 | 0.000 | 0.000 | 0.000 | 0.000 | 0.000 | 0.000 | 0.008 | 0.000 | 0.000 | 0.071 | 0.000 | 0.031 | 0.000 |
| 210 | 0.310 | 0.196 | 0.146 | 0.173 | 0.076 | 0.000 | 0.000 | 0.079 | 0.080 | 0.129 | 0.171 | 0.179 | 0.219 | 0.275 |
| 211 | 0.000 | 0.000 | 0.000 | 0.006 | 0.000 | 0.000 | 0.000 | 0.000 | 0.000 | 0.000 | 0.000 | 0.000 | 0.000 | 0.000 |
| 212 | 0.357 | 0.482 | 0.299 | 0.438 | 0.394 | 0.261 | 0.412 | 0.373 | 0.295 | 0.323 | 0.357 | 0.808 | 0.625 | 0.325 |
| 213 | 0.095 | 0.250 | 0.366 | 0.204 | 0.167 | 0.630 | 0.206 | 0.333 | 0.318 | 0.387 | 0.343 | 0.000 | 0.062 | 0.000 |
| 214 | 0.071 | 0.071 | 0.171 | 0.173 | 0.348 | 0.109 | 0.235 | 0.206 | 0.307 | 0.113 | 0.057 | 0.013 | 0.062 | 0.025 |
| 216 | 0.024 | 0.000 | 0.006 | 0.006 | 0.015 | 0.000 | 0.147 | 0.000 | 0.000 | 0.048 | 0.000 | 0.000 | 0.000 | 0.375 |
| 217 | 0.000 | 0.000 | 0.012 | 0.000 | 0.000 | 0.000 | 0.000 | 0.000 | 0.000 | 0.000 | 0.000 | 0.000 | 0.000 | 0.000 |
| G10O |  |  |  |  |  |  |  |  |  |  |  |  |  |  |
| 150 | 0.000 | 0.000 | 0.000 | 0.000 | 0.000 | 0.000 | 0.000 | 0.000 | 0.000 | 0.000 | 0.000 | 0.000 | 0.000 | 0.100 |
| 154 | 0.024 | 0.054 | 0.067 | 0.086 | 0.000 | 0.000 | 0.000 | 0.008 | 0.000 | 0.000 | 0.014 | 0.026 | 0.062 | 0.000 |
| 156 | 0.000 | 0.018 | 0.000 | 0.000 | 0.000 | 0.000 | 0.000 | 0.000 | 0.000 | 0.000 | 0.000 | 0.000 | 0.000 | 0.000 |
| 180 | 0.000 | 0.000 | 0.000 | 0.000 | 0.000 | 0.000 | 0.000 | 0.000 | 0.000 | 0.000 | 0.000 | 0.000 | 0.000 | 0.050 |
| 192 | 0.000 | 0.000 | 0.000 | 0.000 | 0.000 | 0.000 | 0.000 | 0.000 | 0.000 | 0.000 | 0.000 | 0.000 | 0.000 | 0.475 |
| 194 | 0.000 | 0.000 | 0.006 | 0.093 | 0.000 | 0.000 | 0.000 | 0.000 | 0.000 | 0.065 | 0.000 | 0.000 | 0.000 | 0.000 |
| 196 | 0.571 | 0.696 | 0.854 | 0.747 | 0.909 | 0.891 | 0.941 | 0.873 | 0.989 | 0.887 | 0.943 | 0.949 | 0.906 | 0.000 |
| 198 | 0.405 | 0.214 | 0.061 | 0.056 | 0.061 | 0.000 | 0.044 | 0.000 | 0.000 | 0.000 | 0.000 | 0.000 | 0.000 | 0.050 |
| 204 | 0.000 | 0.000 | 0.000 | 0.000 | 0.000 | 0.000 | 0.000 | 0.000 | 0.000 | 0.000 | 0.000 | 0.000 | 0.000 | 0.025 |
| 208 | 0.000 | 0.018 | 0.012 | 0.019 | 0.030 | 0.087 | 0.015 | 0.000 | 0.000 | 0.048 | 0.000 | 0.000 | 0.000 | 0.125 |
| 210 | 0.000 | 0.000 | 0.000 | 0.000 | 0.000 | 0.022 | 0.000 | 0.119 | 0.011 | 0.000 | 0.043 | 0.026 | 0.031 | 0.175 |
| G10P |  |  |  |  |  |  |  |  |  |  |  |  |  |  |
| 147 | 0.000 | 0.000 | 0.006 | 0.025 | 0.000 | 0.000 | 0.000 | 0.000 | 0.000 | 0.000 | 0.000 | 0.000 | 0.000 | 0.200 |
| 151 | 0.000 | 0.000 | 0.000 | 0.000 | 0.000 | 0.000 | 0.000 | 0.000 | 0.000 | 0.000 | 0.000 | 0.000 | 0.000 | 0.175 |
| 153 | 0.571 | 0.571 | 0.817 | 0.710 | 0.818 | 0.891 | 0.735 | 0.667 | 0.705 | 0.661 | 0.714 | 0.590 | 0.656 | 0.000 |
| 155 | 0.000 | 0.179 | 0.006 | 0.000 | 0.030 | 0.022 | 0.000 | 0.000 | 0.034 | 0.000 | 0.000 | 0.000 | 0.000 | 0.000 |
| 157 | 0.000 | 0.000 | 0.091 | 0.012 | 0.030 | 0.022 | 0.000 | 0.000 | 0.023 | 0.016 | 0.014 | 0.000 | 0.000 | 0.075 |
| 159 | 0.262 | 0.196 | 0.043 | 0.148 | 0.045 | 0.000 | 0.221 | 0.063 | 0.045 | 0.323 | 0.157 | 0.077 | 0.062 | 0.250 |
| 161 | 0.167 | 0.036 | 0.024 | 0.000 | 0.061 | 0.043 | 0.000 | 0.167 | 0.193 | 0.000 | 0.100 | 0.077 | 0.000 | 0.000 |
| 163 | 0.000 | 0.018 | 0.000 | 0.043 | 0.000 | 0.022 | 0.015 | 0.103 | 0.000 | 0.000 | 0.000 | 0.192 | 0.094 | 0.300 |
| 165 | 0.000 | 0.000 | 0.006 | 0.031 | 0.000 | 0.000 | 0.029 | 0.000 | 0.000 | 0.000 | 0.014 | 0.064 | 0.188 | 0.000 |
| 167 | 0.000 | 0.000 | 0.006 | 0.031 | 0.015 | 0.000 | 0.000 | 0.000 | 0.000 | 0.000 | 0.000 | 0.000 | 0.000 | 0.000 |
| G10U |  |  |  |  |  |  |  |  |  |  |  |  |  |  |
| 161 | 0.000 | 0.018 | 0.110 | 0.000 | 0.030 | 0.217 | 0.000 | 0.000 | 0.011 | 0.000 | 0.029 | 0.013 | 0.000 | 0.000 |
| 163 | 0.048 | 0.054 | 0.055 | 0.019 | 0.030 | 0.000 | 0.000 | 0.143 | 0.034 | 0.000 | 0.086 | 0.038 | 0.062 | 0.000 |
| 165 | 0.167 | 0.571 | 0.451 | 0.414 | 0.273 | 0.174 | 0.368 | 0.254 | 0.386 | 0.065 | 0.300 | 0.128 | 0.094 | 0.000 |
| 167 | 0.357 | 0.125 | 0.146 | 0.340 | 0.455 | 0.087 | 0.162 | 0.341 | 0.330 | 0.145 | 0.471 | 0.692 | 0.844 | 0.000 |
| 169 | 0.000 | 0.054 | 0.049 | 0.006 | 0.015 | 0.326 | 0.176 | 0.079 | 0.045 | 0.290 | 0.000 | 0.128 | 0.000 | 0.000 |
| 171 | 0.000 | 0.000 | 0.000 | 0.000 | 0.000 | 0.000 | 0.000 | 0.000 | 0.023 | 0.000 | 0.000 | 0.000 | 0.000 | 0.000 |
| 173 | 0.000 | 0.036 | 0.018 | 0.000 | 0.076 | 0.130 | 0.074 | 0.000 | 0.000 | 0.065 | 0.000 | 0.000 | 0.000 | 0.000 |
| 175 | 0.000 | 0.000 | 0.006 | 0.000 | 0.030 | 0.000 | 0.088 | 0.111 | 0.148 | 0.016 | 0.057 | 0.000 | 0.000 | 0.300 |
| 177 | 0.429 | 0.089 | 0.159 | 0.222 | 0.091 | 0.065 | 0.132 | 0.071 | 0.023 | 0.403 | 0.057 | 0.000 | 0.000 | 0.625 |
| 179 | 0.000 | 0.054 | 0.006 | 0.000 | 0.000 | 0.000 | 0.000 | 0.000 | 0.000 | 0.016 | 0.000 | 0.000 | 0.000 | 0.000 |
| 181 | 0.000 | 0.000 | 0.000 | 0.000 | 0.000 | 0.000 | 0.000 | 0.000 | 0.000 | 0.000 | 0.000 | 0.000 | 0.000 | 0.075 |
| G10X |  |  |  |  |  |  |  |  |  |  |  |  |  |  |
| 161 | 0.000 | 0.018 | 0.110 | 0.000 | 0.030 | 0.217 | 0.000 | 0.000 | 0.011 | 0.000 | 0.029 | 0.013 | 0.000 | 0.000 |
| 163 | 0.048 | 0.054 | 0.055 | 0.019 | 0.030 | 0.000 | 0.000 | 0.143 | 0.034 | 0.000 | 0.086 | 0.038 | 0.062 | 0.000 |
| 165 | 0.167 | 0.571 | 0.451 | 0.414 | 0.273 | 0.174 | 0.368 | 0.254 | 0.386 | 0.065 | 0.300 | 0.128 | 0.094 | 0.000 |
| 167 | 0.357 | 0.125 | 0.146 | 0.340 | 0.455 | 0.087 | 0.162 | 0.341 | 0.330 | 0.145 | 0.471 | 0.692 | 0.844 | 0.000 |
| 169 | 0.000 | 0.054 | 0.049 | 0.006 | 0.015 | 0.326 | 0.176 | 0.079 | 0.045 | 0.290 | 0.000 | 0.128 | 0.000 | 0.000 |
| 171 | 0.000 | 0.000 | 0.000 | 0.000 | 0.000 | 0.000 | 0.000 | 0.000 | 0.023 | 0.000 | 0.000 | 0.000 | 0.000 | 0.000 |
| 173 | 0.000 | 0.036 | 0.018 | 0.000 | 0.076 | 0.130 | 0.074 | 0.000 | 0.000 | 0.065 | 0.000 | 0.000 | 0.000 | 0.000 |
| 175 | 0.000 | 0.000 | 0.006 | 0.000 | 0.030 | 0.000 | 0.088 | 0.111 | 0.148 | 0.016 | 0.057 | 0.000 | 0.000 | 0.300 |
| 177 | 0.429 | 0.089 | 0.159 | 0.222 | 0.091 | 0.065 | 0.132 | 0.071 | 0.023 | 0.403 | 0.057 | 0.000 | 0.000 | 0.625 |
| 179 | 0.000 | 0.054 | 0.006 | 0.000 | 0.000 | 0.000 | 0.000 | 0.000 | 0.000 | 0.016 | 0.000 | 0.000 | 0.000 | 0.000 |
| 181 | 0.000 | 0.000 | 0.000 | 0.000 | 0.000 | 0.000 | 0.000 | 0.000 | 0.000 | 0.000 | 0.000 | 0.000 | 0.000 | 0.075 |
| MU50 |  |  |  |  |  |  |  |  |  |  |  |  |  |  |
| 120 | 0.000 | 0.000 | 0.000 | 0.000 | 0.000 | 0.000 | 0.000 | 0.000 | 0.000 | 0.000 | 0.000 | 0.000 | 0.000 | 0.250 |
| 122 | 0.976 | 0.804 | 0.988 | 0.951 | 0.879 | 0.870 | 0.897 | 0.929 | 0.909 | 0.952 | 0.943 | 1.000 | 1.000 | 0.075 |
| 124 | 0.000 | 0.000 | 0.000 | 0.006 | 0.000 | 0.000 | 0.000 | 0.000 | 0.000 | 0.016 | 0.000 | 0.000 | 0.000 | 0.100 |
| 126 | 0.000 | 0.000 | 0.000 | 0.000 | 0.030 | 0.000 | 0.000 | 0.000 | 0.057 | 0.000 | 0.000 | 0.000 | 0.000 | 0.200 |
| 132 | 0.000 | 0.000 | 0.006 | 0.019 | 0.015 | 0.000 | 0.015 | 0.000 | 0.000 | 0.000 | 0.000 | 0.000 | 0.000 | 0.025 |
| 134 | 0.024 | 0.196 | 0.000 | 0.006 | 0.076 | 0.130 | 0.088 | 0.071 | 0.034 | 0.032 | 0.057 | 0.000 | 0.000 | 0.000 |
| 136 | 0.000 | 0.000 | 0.000 | 0.000 | 0.000 | 0.000 | 0.000 | 0.000 | 0.000 | 0.000 | 0.000 | 0.000 | 0.000 | 0.025 |
| 138 | 0.000 | 0.000 | 0.000 | 0.000 | 0.000 | 0.000 | 0.000 | 0.000 | 0.000 | 0.000 | 0.000 | 0.000 | 0.000 | 0.100 |
| 140 | 0.000 | 0.000 | 0.006 | 0.019 | 0.000 | 0.000 | 0.000 | 0.000 | 0.000 | 0.000 | 0.000 | 0.000 | 0.000 | 0.200 |
| 144 | 0.000 | 0.000 | 0.000 | 0.000 | 0.000 | 0.000 | 0.000 | 0.000 | 0.000 | 0.000 | 0.000 | 0.000 | 0.000 | 0.025 |
| MU59 |  |  |  |  |  |  |  |  |  |  |  |  |  |  |
| 231 | 0.333 | 0.804 | 0.518 | 0.506 | 0.545 | 0.522 | 0.515 | 0.429 | 0.523 | 0.742 | 0.643 | 0.756 | 0.750 | 0.000 |
| 235 | 0.095 | 0.018 | 0.006 | 0.000 | 0.121 | 0.000 | 0.206 | 0.071 | 0.000 | 0.000 | 0.114 | 0.051 | 0.094 | 0.000 |
| 237 | 0.095 | 0.054 | 0.110 | 0.019 | 0.076 | 0.217 | 0.103 | 0.000 | 0.011 | 0.000 | 0.000 | 0.000 | 0.000 | 0.000 |
| 239 | 0.000 | 0.000 | 0.085 | 0.093 | 0.015 | 0.022 | 0.000 | 0.000 | 0.000 | 0.048 | 0.000 | 0.000 | 0.000 | 0.775 |
| 241 | 0.167 | 0.000 | 0.140 | 0.062 | 0.030 | 0.109 | 0.059 | 0.032 | 0.057 | 0.000 | 0.014 | 0.026 | 0.094 | 0.050 |
| 243 | 0.048 | 0.125 | 0.104 | 0.136 | 0.152 | 0.109 | 0.118 | 0.349 | 0.273 | 0.081 | 0.171 | 0.013 | 0.000 | 0.175 |
| 245 | 0.024 | 0.000 | 0.037 | 0.185 | 0.061 | 0.022 | 0.000 | 0.119 | 0.136 | 0.129 | 0.057 | 0.154 | 0.062 | 0.000 |
| 247 | 0.238 | 0.000 | 0.000 | 0.000 | 0.000 | 0.000 | 0.000 | 0.000 | 0.000 | 0.000 | 0.000 | 0.000 | 0.000 | 0.000 |

^a^ Boulder Mountain (BM), ^b^ La Sal Mountains (LSM), ^c^ San Juan and Chuska mountains (SJC), ^d^ Sangre de Cristo Mountains (SCM), ^e^ Zuni Mountains (ZM), ^f^ Mt. Taylor (MT), ^g^ Sandia and Manzano mountains (SMM), ^h^ Mogollon Rim (MR), ^I^ Gila complex (GC), ^j^ Sacramento Mountains (SM), ^k^ Sky Islands north of Interstate-10 (SIN), ^l^ Huachuca and Santa Rita mountains (HSRM), ^m^ Chiricahua complex (CHC), ^n^ Trans-Pecos region (TP).

Table D.11. Estimated pairwise-relative migration rates for regional subpopulations identified by GENELAND using the uncorrelated allele frequency model for American black bears (*Ursus americanus*) in the American Southwest and northern Mexico. Bolded values indicate significant asymmetric migration based on 1,000 bootstrap iterations. Table is interpreted as migration occurring from the cluster in the left-hand column to the cluster in the top row.

| Cluster | BM^a^ | LSM^b^ | SJC^c^ | SCM^d^ | ZM^e^ | MT^f^ | SMM^g^ | MR^h^ | GC^i^ | SM^j^ | SIN^k^ | HSRM^l^ | CHC^m^ | TP^n^ |
| --- | --- | --- | --- | --- | --- | --- | --- | --- | --- | --- | --- | --- | --- | --- |
| BM | -- | 0.09 | 0.11 | 0.10 | 0.09 | 0.04 | 0.07 | 0.07 | 0.06 | 0.05 | 0.06 | 0.04 | 0.04 | 0.02 |
| LSM | 0.09 | -- | 0.35 | 0.33 | 0.28 | 0.17 | 0.17 | 0.16 | 0.13 | 0.11 | 0.13 | 0.07 | 0.08 | 0.01 |
| SJC | 0.12 | 0.43 | -- | 0.64 | 0.52 | 0.47 | 0.37 | 0.29 | **0.23** | 0.22 | 0.19 | 0.08 | 0.10 | 0.01 |
| SCM | 0.10 | 0.28 | 0.63 | -- | 0.32 | 0.22 | 0.29 | 0.25 | 0.17 | 0.17 | **0.14** | 0.08 | 0.09 | 0.02 |
| ZM | 0.07 | 0.30 | 0.66 | 0.45 | -- | 0.42 | 0.41 | 0.55 | 0.37 | 0.23 | 0.31 | 0.09 | 0.13 | **0.01** |
| MT | 0.07 | 0.25 | 0.48 | 0.22 | 0.31 | -- | 0.33 | 0.21 | 0.17 | 0.22 | 0.12 | 0.05 | 0.06 | 0.01 |
| SMM | 0.08 | 0.27 | 0.40 | 0.33 | 0.33 | 0.25 | -- | 0.21 | 0.18 | 0.27 | **0.13** | **0.07** | 0.08 | 0.02 |
| MR | 0.06 | 0.25 | 0.39 | 0.29 | 0.65 | 0.29 | 0.30 | -- | 0.75 | 0.22 | 0.58 | 0.09 | 0.13 | 0.01 |
| GC | 0.05 | 0.27 | **0.59** | 0.30 | 0.78 | 0.37 | 0.31 | 1.00 | -- | 0.22 | 0.35 | 0.08 | 0.13 | 0.01 |
| SM | 0.06 | 0.20 | 0.21 | 0.16 | 0.15 | 0.12 | 0.35 | 0.14 | 0.10 | -- | **0.10** | **0.05** | **0.05** | 0.01 |
| SIN | 0.06 | 0.23 | 0.37 | **0.37** | 0.61 | 0.23 | **0.34** | 0.82 | 0.50 | **0.24** | -- | 0.12 | 0.22 | 0.01 |
| HSRM | 0.03 | 0.11 | 0.13 | 0.15 | 0.19 | 0.11 | **0.15** | 0.13 | 0.13 | **0.12** | 0.17 | -- | 0.22 | 0.01 |
| CHC | 0.05 | 0.11 | 0.14 | 0.18 | 0.12 | 0.10 | 0.14 | 0.14 | 0.14 | **0.13** | 0.22 | 0.19 | -- | 0.01 |
| TP | 0.01 | 0.02 | 0.02 | 0.03 | **0.03** | 0.01 | 0.02 | 0.02 | 0.02 | 0.02 | 0.01 | 0.01 | 0.01 | -- |

^a^ Boulder Mountain (BM), ^b^ La Sal Mountains (LSM), ^c^ San Juan and Chuska mountains (SJC), ^d^ Sangre de Cristo Mountains (SCM), ^e^ Zuni Mountains (ZM), ^f^ Mt. Taylor (MT), ^g^ Sandia and Manzano mountains (SMM), ^h^ Mogollon Rim (MR), ^i^ Gila complex (GC), ^j^ Sacramento Mountains (SM), ^k^ Sky Islands north of Interstate 10 (SIN), ^l^ Huachuca and Santa Rita mountains (HSRM), ^m^ Chiricahua complex (CHC), ^n^ Trans-Pecos region (TP).

## Appendix S5. Resistance surface optimization results

Table S1. Model selection results for optimization run 1 derived using Akaike’s Information Criterion corrected for small sample size (AICc) comparing resistance surfaces optimized using linear-mixed models with maximum likelihood population effects parameterization. Models were ranked by the difference in AICc (ΔAICc) between the top model and competing models. The number of parameters fit in each model is represented by K.

| Surface | K | AIC_C_ | ΔAIC_C_ | *w_i_* |
| --- | --- | --- | --- | --- |
| canopy + precip + TRI | 10 | -387083.77 | 0.00 | 1.00 |
| canopy + precip + streams | 9 | -387036.59 | 47.18 | 0.00 |
| canopy + precip | 7 | -386985.04 | 98.73 | 0.00 |
| canopy + precip + rd.major | 11 | -386895.84 | 187.94 | 0.00 |
| canopy + precip + water bodies | 9 | -386891.03 | 192.74 | 0.00 |
| canopy + rd.density | 7 | -386015.11 | 1068.66 | 0.00 |
| canopy + rd.density + TRI | 10 | -385960.90 | 1122.88 | 0.00 |
| canopy + rd.major + rd.density | 11 | -385907.57 | 1176.21 | 0.00 |
| canopy + precip + rd.density | 10 | -385853.13 | 1230.64 | 0.00 |
| canopy + rd.density + streams | 9 | -385831.51 | 1252.27 | 0.00 |
| canopy | 4 | -385331.52 | 1752.25 | 0.00 |
| canopy + rd.density + water bodies | 9 | -385330.54 | 1753.23 | 0.00 |
| canopy + TRI | 7 | -385321.23 | 1762.55 | 0.00 |
| canopy + streams | 6 | -385317.48 | 1766.29 | 0.00 |
| canopy + water bodies | 6 | -385315.24 | 1768.53 | 0.00 |
| canopy + rd.major | 8 | -385301.98 | 1781.80 | 0.00 |
| canopy + streams + TRI | 9 | -385290.97 | 1792.81 | 0.00 |
| canopy + TRI + water bodies | 9 | -385279.78 | 1803.99 | 0.00 |
| canopy + rd.major + streams | 10 | -385223.35 | 1860.43 | 0.00 |
| canopy + streams + water bodies | 8 | -385197.60 | 1886.17 | 0.00 |
| canopy + rd.major + water bodies | 10 | -385195.15 | 1888.62 | 0.00 |
| canopy + rd.major + TRI | 11 | -385185.59 | 1898.19 | 0.00 |
| precip + rd.density | 7 | -384594.20 | 2489.58 | 0.00 |
| precip + rd.density + streams | 9 | -384536.41 | 2547.36 | 0.00 |
| water bodies | 3 | -384370.56 | 2713.22 | 0.00 |
| precip + water bodies | 6 | -384323.56 | 2760.21 | 0.00 |
| rd.major + water bodies | 7 | -384281.53 | 2802.24 | 0.00 |
| rd.major + streams + water bodies | 9 | -384258.60 | 2825.17 | 0.00 |
| precip + streams | 6 | -384246.96 | 2836.81 | 0.00 |
| precip + TRI | 7 | -384226.84 | 2856.93 | 0.00 |
| precip | 4 | -384220.62 | 2863.15 | 0.00 |
| precip + streams + TRI | 9 | -384201.10 | 2882.67 | 0.00 |
| streams + water bodies | 5 | -384177.91 | 2905.86 | 0.00 |
| precip + rd.density + water bodies | 9 | -384149.89 | 2933.88 | 0.00 |
| precip + rd.density + TRI | 10 | -384140.50 | 2943.27 | 0.00 |
| precip + rd.major + TRI | 11 | -384072.69 | 3011.09 | 0.00 |
| precip + rd.major + rd.density | 11 | -383995.40 | 3088.37 | 0.00 |
| precip + TRI + water bodies | 9 | -383968.64 | 3115.13 | 0.00 |
| precip + streams + water bodies | 8 | -383932.86 | 3150.92 | 0.00 |
| precip + rd.major | 8 | -383817.89 | 3265.89 | 0.00 |
| precip + rd.major + water bodies | 10 | -383722.29 | 3361.48 | 0.00 |
| precip + rd.major + streams | 10 | -383440.57 | 3643.20 | 0.00 |
| rd.density + streams + water bodies | 8 | -383424.75 | 3659.02 | 0.00 |
| streams + TRI + water bodies | 8 | -383264.02 | 3819.75 | 0.00 |
| rd.major + rd.density + water bodies | 10 | -383190.57 | 3893.20 | 0.00 |
| TRI + water bodies | 6 | -383110.30 | 3973.47 | 0.00 |
| rd.density + water bodies | 6 | -382820.35 | 4263.42 | 0.00 |
| rd.density | 4 | -382652.78 | 4430.99 | 0.00 |
| rd.density + streams | 6 | -382644.08 | 4439.69 | 0.00 |
| rd.density + TRI | 7 | -382627.91 | 4455.87 | 0.00 |
| rd.density + TRI + water bodies | 9 | -382521.03 | 4562.74 | 0.00 |
| rd.density + streams + TRI | 9 | -382501.60 | 4582.18 | 0.00 |
| rd.major + rd.density + TRI | 11 | -382465.22 | 4618.55 | 0.00 |
| rd.major + TRI + water bodies | 10 | -382450.31 | 4633.46 | 0.00 |
| rd.major + rd.density | 8 | -382405.35 | 4678.43 | 0.00 |
| rd.major + rd.density + streams | 10 | -382397.56 | 4686.21 | 0.00 |
| rd.major + streams | 7 | -382216.61 | 4867.17 | 0.00 |
| rd.major + streams + TRI | 10 | -382124.71 | 4959.07 | 0.00 |
| streams | 3 | -382076.94 | 5006.84 | 0.00 |
| rd.major + TRI | 8 | -381969.30 | 5114.47 | 0.00 |
| streams + TRI | 6 | -381959.97 | 5123.80 | 0.00 |
| TRI | 4 | -381955.78 | 5127.99 | 0.00 |
| rd.major | 5 | -379702.36 | 7381.41 | 0.00 |

Table S2. Model selection results for optimization run 2 derived using Akaike’s Information Criterion corrected for small sample size (AICc) comparing resistance surfaces optimized using linear-mixed models with maximum likelihood population effects parameterization. Models were ranked by the difference in AICc (ΔAICc) between the top model and competing models. The number of parameters fit in each model is represented by K.

| Surface | K | AIC_C_ | ΔAIC_C_ | *w_i_* |
| --- | --- | --- | --- | --- |
| canopy + precip + TRI | 10 | -387083.77 | 0.00 | 1.00 |
| canopy + precip + streams | 9 | -387036.59 | 47.18 | 0.00 |
| canopy + precip | 7 | -386985.04 | 98.73 | 0.00 |
| canopy + precip + rd.major | 11 | -386895.84 | 187.94 | 0.00 |
| canopy + precip + water bodies | 9 | -386891.03 | 192.74 | 0.00 |
| canopy + rd.density | 7 | -386015.11 | 1068.66 | 0.00 |
| canopy + rd.density + TRI | 10 | -385960.90 | 1122.88 | 0.00 |
| canopy + rd.major + rd.density | 11 | -385907.57 | 1176.21 | 0.00 |
| canopy + precip + rd.density | 10 | -385853.13 | 1230.64 | 0.00 |
| canopy + rd.density + streams | 9 | -385831.51 | 1252.27 | 0.00 |
| canopy | 4 | -385333.12 | 1750.65 | 0.00 |
| canopy + rd.density + water bodies | 9 | -385330.54 | 1753.23 | 0.00 |
| canopy + TRI | 7 | -385321.23 | 1762.55 | 0.00 |
| canopy + streams | 6 | -385317.48 | 1766.29 | 0.00 |
| canopy + water bodies | 6 | -385315.24 | 1768.53 | 0.00 |
| canopy + rd.major | 8 | -385301.98 | 1781.80 | 0.00 |
| canopy + streams + TRI | 9 | -385290.97 | 1792.81 | 0.00 |
| canopy + TRI + water bodies | 9 | -385279.78 | 1803.99 | 0.00 |
| canopy + rd.major + streams | 10 | -385223.35 | 1860.43 | 0.00 |
| canopy + streams + water bodies | 8 | -385197.60 | 1886.17 | 0.00 |
| canopy + rd.major + water bodies | 10 | -385195.15 | 1888.62 | 0.00 |
| canopy + rd.major + TRI | 11 | -385185.59 | 1898.19 | 0.00 |
| precip + rd.density | 7 | -384594.20 | 2489.58 | 0.00 |
| precip + rd.density + streams | 9 | -384536.41 | 2547.36 | 0.00 |
| streams + water bodies | 5 | -384364.71 | 2719.06 | 0.00 |
| water bodies | 3 | -384351.17 | 2732.60 | 0.00 |
| rd.barrier + streams + water bodies | 9 | -384340.65 | 2743.13 | 0.00 |
| rd.barrier + water bodies | 7 | -384327.52 | 2756.25 | 0.00 |
| precip + water bodies | 6 | -384301.97 | 2781.81 | 0.00 |
| precip + streams | 6 | -384246.96 | 2836.81 | 0.00 |
| precip | 4 | -384230.68 | 2853.09 | 0.00 |
| precip + TRI | 7 | -384226.84 | 2856.93 | 0.00 |
| precip + streams + TRI | 9 | -384201.10 | 2882.67 | 0.00 |
| precip + rd.density + water bodies | 9 | -384149.89 | 2933.88 | 0.00 |
| precip + rd.density + TRI | 10 | -384140.50 | 2943.27 | 0.00 |
| TRI + water bodies | 6 | -384135.03 | 2948.74 | 0.00 |
| precip + rd.major + TRI | 11 | -384072.69 | 3011.09 | 0.00 |
| rd.density + water bodies | 6 | -384002.72 | 3081.05 | 0.00 |
| precip + rd.major + rd.density | 11 | -383995.40 | 3088.37 | 0.00 |
| precip + TRI + water bodies | 9 | -383968.64 | 3115.13 | 0.00 |
| precip + streams + water bodies | 8 | -383932.86 | 3150.92 | 0.00 |
| precip + rd.major | 8 | -383817.89 | 3265.89 | 0.00 |
| precip + rd.major + water bodies | 10 | -383722.29 | 3361.48 | 0.00 |
| precip + rd.major + streams | 10 | -383440.57 | 3643.20 | 0.00 |
| rd.density + streams + water bodies | 8 | -383424.75 | 3659.02 | 0.00 |
| streams + TRI + water bodies | 8 | -383264.02 | 3819.75 | 0.00 |
| rd.major + rd.density + water bodies | 10 | -383190.57 | 3893.20 | 0.00 |
| rd.density + TRI | 7 | -382645.98 | 4437.80 | 0.00 |
| rd.density | 4 | -382640.30 | 4443.48 | 0.00 |
| rd.density + streams | 6 | -382599.32 | 4484.45 | 0.00 |
| rd.density + TRI + water bodies | 9 | -382521.03 | 4562.74 | 0.00 |
| rd.density + streams + TRI | 9 | -382501.60 | 4582.18 | 0.00 |
| rd.major + rd.density + TRI | 11 | -382465.22 | 4618.55 | 0.00 |
| rd.barrier + rd.density | 8 | -382459.35 | 4624.42 | 0.00 |
| rd.major + TRI + water bodies | 10 | -382450.31 | 4633.46 | 0.00 |
| rd.major + rd.density + streams | 10 | -382397.56 | 4686.21 | 0.00 |
| streams + TRI | 6 | -382396.82 | 4686.95 | 0.00 |
| rd.major + streams | 7 | -382181.33 | 4902.45 | 0.00 |
| rd.major + streams + TRI | 10 | -382124.71 | 4959.07 | 0.00 |
| streams | 3 | -382089.36 | 4994.41 | 0.00 |
| rd.major + TRI | 8 | -381958.89 | 5124.88 | 0.00 |
| TRI | 4 | -381900.95 | 5182.82 | 0.00 |
| rd.major | 5 | -381772.80 | 5310.97 | 0.00 |
